# Destabilized adaptive influenza variants critical for innate immune system escape are potentiated by host chaperones

**DOI:** 10.1371/journal.pbio.3000008

**Published:** 2018-09-17

**Authors:** Angela M. Phillips, Anna I. Ponomarenko, Kenny Chen, Orr Ashenberg, Jiayuan Miao, Sean M. McHugh, Vincent L. Butty, Charles A. Whittaker, Christopher L. Moore, Jesse D. Bloom, Yu-Shan Lin, Matthew D. Shoulders

**Affiliations:** 1 Department of Chemistry, Massachusetts Institute of Technology, Cambridge, Massachusetts, United States of America; 2 Fred Hutchinson Cancer Research Center, Seattle, Washington, United States of America; 3 Department of Chemistry, Tufts University, Medford, Massachusetts, United States of America; 4 BioMicro Center, Massachusetts Institute of Technology, Cambridge, Massachusetts, United States of America; ETH Zurich, SWITZERLAND

## Abstract

The threat of viral pandemics demands a comprehensive understanding of evolution at the host–pathogen interface. Here, we show that the accessibility of adaptive mutations in influenza nucleoprotein at fever-like temperatures is mediated by host chaperones. Particularly noteworthy, we observe that the Pro283 nucleoprotein variant, which (1) is conserved across human influenza strains, (2) confers resistance to the Myxovirus resistance protein A (MxA) restriction factor, and (3) critically contributed to adaptation to humans in the 1918 pandemic influenza strain, is rendered unfit by heat shock factor 1 inhibition–mediated host chaperone depletion at febrile temperatures. This fitness loss is due to biophysical defects that chaperones are unavailable to address when heat shock factor 1 is inhibited. Thus, influenza subverts host chaperones to uncouple the biophysically deleterious consequences of viral protein variants from the benefits of immune escape. In summary, host proteostasis plays a central role in shaping influenza adaptation, with implications for the evolution of other viruses, for viral host switching, and for antiviral drug development.

## Abstract 2

Host chaperones enable the influenza virus to evade the host’s innate immune response by ameliorating the biophysically deleterious consequences of adaptive mutations (such as the nucleoprotein Pro283 variant that strengthened the 1918 pandemic strain).

## Author summary

Viruses, such as influenza, evade the host immune response by mutating frequently. However, these adaptive amino acid substitutions are often biophysically deleterious and can thus increase the propensity for viral proteins to misfold and hamper viral replication. Host protein folding factors called chaperones interact extensively with viral proteins, like influenza nucleoprotein, and are thus poised to potentiate the fitness of biophysically defective, adaptive variants. Here, we directly test this hypothesis by quantitatively profiling the mutational tolerance of influenza nucleoprotein in host cells with reduced chaperone levels. We find that chaperones indeed increase the accessibility of destabilized adaptive nucleoprotein variants, with an especially strong effect at fever-like temperatures. We observe that the destabilized Pro283 nucleoprotein variant, which is universally conserved across human influenza strains and enables evasion of the Myxovirus resistance protein A (MxA) innate immunity restriction factor, is rendered unfit in a chaperone-depleted host environment. Together, these data show that host chaperones critically impact viral adaptation and may serve as targets for antiviral therapeutic adjuvants.

## Introduction

RNA viruses are exceptionally efficient pathogens that leverage host machineries to replicate their genetic material, synthesize their proteins, and assemble new virions. Perhaps their most remarkable feature, however, is the capacity to rapidly evolve in the face of environmental and immune system pressures. Such rapid evolution is largely mediated by a high mutation rate [1].

Despite its adaptive benefits, rapid genetic change comes at a significant cost for evolving proteins. The majority of amino acid substitutions (especially functionally relevant substitutions that alter or create protein activities) are biophysically deleterious, negatively affecting either protein folding or stability [2–6].

A striking illustration of this phenomenon in RNA viruses is influenza nucleoprotein (NP). NP is a globular protein that oligomerizes to encapsulate influenza genomic material and mediate its import into the host nucleus, a process that is required for transcription and replication of the viral genome [7]. NP is strongly conserved relative to the highly variable influenza surface proteins targeted by antibodies [8]. However, NP does experience significant selection pressure from the host immune system, including from innate immune restriction factors [9, 10]. In particular, the human restriction factor Myxovirus resistance protein A (MxA) can prevent influenza ribonucleoprotein import [11–13], cutting short the viral replication cycle.

Adaptive mutations in NP that allow escape from human MxA are critical for the efficient replication of new influenza strains in humans following zoonotic transmission. In just the last century, nonhuman influenza NP was introduced into circulating human influenza strains in 2009 and probably in 1918 [14, 15], leading to the acquisition of MxA resistance and ultimately to global pandemics [16, 17]. While NP evolution is driven by immune escape, it is nonetheless clear that a delicate balance exists between immune system resistance and NP stability and folding. Several NP substitutions known to engender immune escape are destabilizing [9, 10], impairing viral growth in the absence of immune pressure [16]. Apparently, the evolving virus must balance the costs of a NP folding defect with the benefits of escaping host immunity.

In theory, any mechanism that allows influenza (or other viruses) to uncouple protein folding versus immune escape selection pressures would have tremendous benefits for the pathogen. Recent work by us and others suggests that the host’s heat shock protein 90 (Hsp90) chaperone can modulate the evolutionary paths traversed by viruses [18, 19]. Neither the mechanism of Hsp90’s effect on viral evolution nor its relevance to actual viral strains is clear. A provocative possibility, not yet experimentally explored, is that hijacked host chaperones, whether Hsp90 or any of the other dozens of chaperones beyond Hsp90 that interact extensively with influenza [20, 21], potentiate viral evolution directly by assisting the folding of biophysically defective NP variants that would otherwise be insufficiently fit to persist in the population. If this hypothesis is correct, it would suggest that subversion of host protein folding chaperones by viruses can make otherwise inaccessible mutational trajectories leading to immune system escape possible, specifically by promoting the folding of escape variants. Such a mechanism for host adaptation would have broad implications for the evolution of not only influenza NP but also other influenza proteins and other viruses.

Rigorously testing this hypothesis requires a method to systematically and quantitatively evaluate whether and how host proteostasis modulates viral protein mutational tolerance. Here, we achieve that objective using deep mutational scanning [22] of influenza NP. We apply deep mutational scanning in the context of chemical genetic inhibition of the host’s heat shock factor 1 (HSF1 [23]) to create biophysically challenging, chaperone-depleted cellular protein folding environments. This high-throughput approach revealed a number of amino acid positions in influenza NP whose mutational tolerance is strongly reduced in chaperone-depleted host cells. We confirmed the strong effects of host chaperones on NP mutational tolerance at a number of these sites using head-to-head competition experiments. Most strikingly, the strongly conserved proline (Pro) residue at site 283 in NP is rendered highly unfit by HSF1 inhibition at febrile temperatures. Pro283 in NP is known to facilitate escape from the human innate immune system restriction factor MxA [9, 16], a feature that critically enhanced the pathogenicity and fitness of the 1918 pandemic influenza strain. We further show that Pro283 disrupts a key structural element in NP, rendering it unstable and aggregation-prone. Host chaperones resolve this folding problem, allowing Pro283 to persist in the viral population and thereby promoting MxA escape.

Collectively, our data demonstrate that viral hijacking of host chaperones addresses critical biophysical defects that would otherwise sensitize the virus to host restriction factors. This phenomenon thus has tremendous impact on the capacity of viruses to adapt to their environments, emphasizing the central importance of a hitherto underappreciated element of the host–pathogen interaction and potentially providing new targets for antiviral intervention.

## Results

### Quantifying NP variant fitness in distinctive host cell folding environments

We used a deep mutational scanning strategy to systematically and experimentally quantify the fitness of nearly all viable single amino acid substitutions in influenza NP in both basal and biophysically challenging host cell environments. To this end, we employed previously reported duplicate NP mutant libraries based on the human-adapted A/Aichi/2/1968 (H3N2) influenza strain [24] and competed each viral library in Madin Darby canine kidney (MDCK) cells. The MxA orthologs in MDCK cells are inactive against all influenza strains tested to date [25] and permit robust influenza growth. To create a chaperone-depleted host cell environment for viral propagation, we employed a highly specific, chemically inducible dominant negative form of HSF1 [23], which is the master regulator of cytosolic and nuclear chaperone levels [26]. At both permissive (37°C) and biophysically restrictive, fever-like (39°C) temperatures, chemical induction of the HSF1 inhibitor reduces cytosolic chaperone transcript and protein levels in both the absence (S1 and S2A Figs; S1 and S2 Data) and presence (S2A and S2B Fig; S2 Data) of influenza. Notably, the condition in which HSF1 was inhibited (HSF1i) did not significantly alter the replication of wild-type influenza or host cell metabolic fitness over the course of our experiment (S2C and S2D Fig; S2 Data). We anticipated that the fever-like temperature would prove moderately more challenging for NP folding, potentially restricting the accessible mutational landscape, while the depletion of cytosolic chaperones at each temperature would assess the potential function of host chaperones in regulating NP variant fitness.

**Fig 1:**
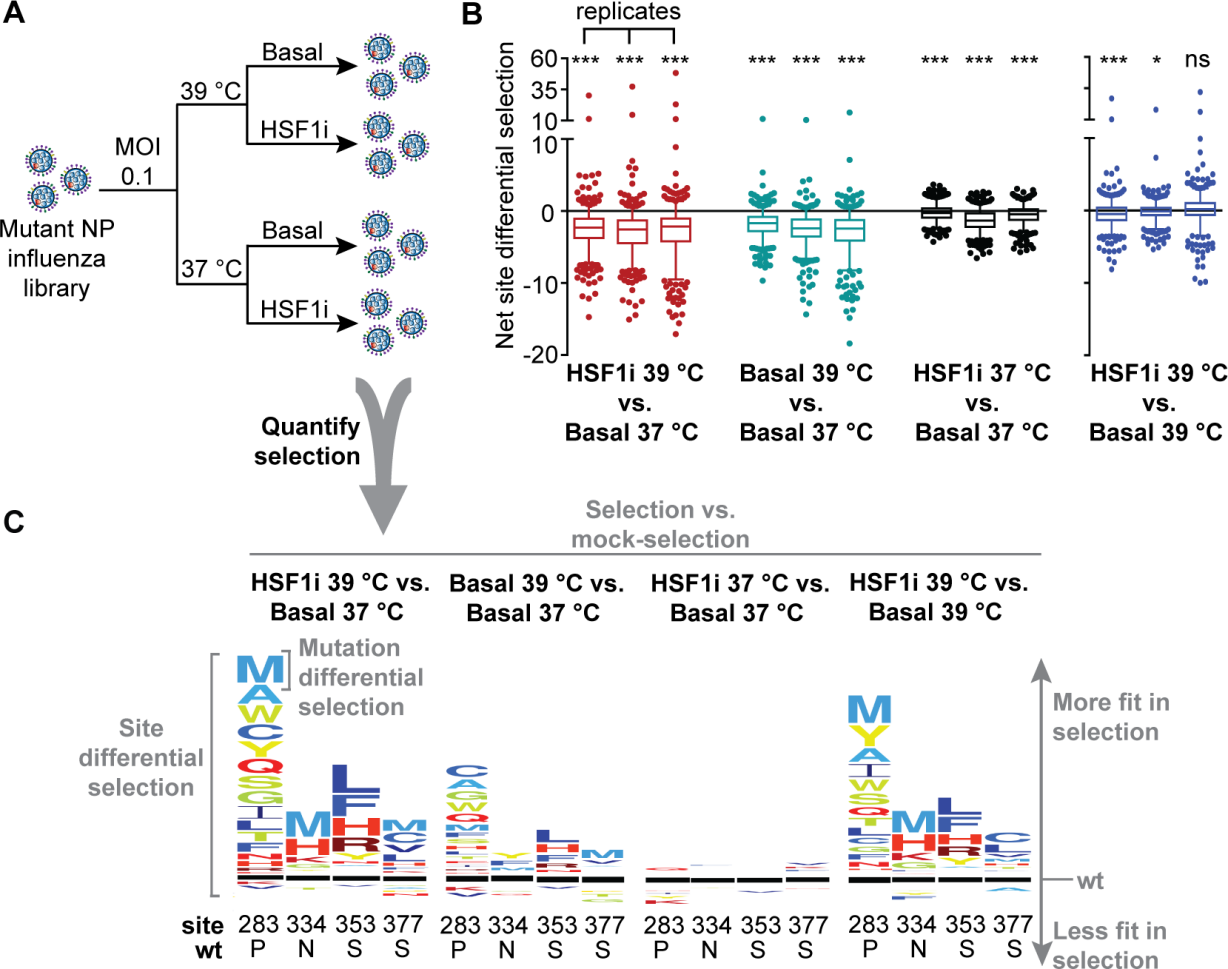
Deep mutational scanning reveals positively selected sites upon chaperone depletion at a biophysically restrictive temperature. (A) Deep mutational scanning selection scheme: each mutant NP influenza library was subjected to selection at 37°C and 39°C in both basal and chaperone-depleted (HSF1i) cells at an MOI of 0.1 virions/cell. Libraries were deep sequenced following replication to quantify the selection on each mutant in the library. Mutation differential selection corresponds to the differential selection of a specific NP mutant, relative to wt NP. Site differential selection corresponds to the sum of the mutation differential selection values for all variants at a given NP site. (B) Box plots showing the distributions of net site differential selection in both basal and chaperone-depleted (HSF1i) cells at 37°C and 39°C. Each selection was performed in biological triplicate; box plots are shown for each replicate. The whiskers show 5th and 95th percentiles, boxes show 25th and 75th percentiles, line shows median value, and points show outliers. The significance of deviation of the mean from zero (no selection) was evaluated by a one-sample *t* test; * and *** indicate FDRs < 0.05 and < 0.0001, respectively. Nested ANOVA accounting for replicates and treatment conditions was performed for all selection conditions normalized to Basal 37°C (*p* = 0.0031). For pairwise statistical comparisons, *p*-values are provided in the statistics section of the Methods. Site differential selection values are provided in S3 Data. (C) Representative sequence logo plots showing amino acid variants at select sites differentially selected upon relative to wt. The size of the amino acid letters corresponds to the magnitude of the mutational differential selection, which is on the same scale for each selection. Amino acids above the black line are more fit in the selection condition compared to the mock-selection condition, amino acids below are less fit, and the black line represents behavior of the wt amino acid in the selection condition. ANOVA, analysis of variance; FDR, false discovery rate; HSF1i, condition in which heat shock factor 1 was inhibited; MOI, multiplicity of infection; NP, nucleoprotein; ns, not significant; wt, wild type.

To maintain library diversity (approximately 10,000 single amino acid substitutions) whilst minimizing coinfection, we infected 10^7^ cells in each host environment (Fig 1A) with 10^6^ infectious virions from our biological duplicate viral NP libraries. In addition to performing deep mutational scanning in biological duplicate, we also performed technical duplicates with one of the replicate libraries (S3A Fig). Following a 48-hour infection, we utilized a previously reported barcoded subamplicon sequencing strategy [27] to accurately quantify the abundance of NP variants after replication at both 37°C and 39°C with and without host chaperone depletion (S3B–S3D Fig). The change in variant frequency upon selection was then normalized to that of the wild-type residue, such that the resulting differential selection [28] value provides a quantitative measure of the relative fitness of each NP variant in the conditions tested. The results of this analysis can be visualized on sequence logo plots (see S4–S7 Figs for complete NP logo plots and S3 and S4 Data for complete differential selection data).

As expected, we found that the fever-like temperature is restrictive, generally reducing the fitness of NP variants relative to the wild-type sequence (see net site differential selection plots in Fig 1B and also the logo plot in S6 Fig—Basal 39°C versus Basal 37°C). Host chaperone depletion caused by inhibition of HSF1 modestly reduces NP variant fitness on average (Fig 1B and S5 and S7 Figs—HSF1i 39°C versus Basal 39°C and HSF1i 37°C versus Basal 37°C), whereas chaperone depletion at the restrictive, fever-like temperature substantially reduces the fitness of NP variants (Fig 1B and S4 Fig—HSF1i 39°C versus Basal 37°C; statistical analyses in Materials and methods). Most strikingly, chaperone depletion at an elevated temperature results in very high levels of differential selection at several specific sites in NP, including sites 283, 334, 353, and 377 (Fig 1C and S4 Fig—HSF1i 39°C versus Basal 37°C). At each of these sites, multiple amino acids confer strongly enhanced fitness relative to the wild-type residue. This phenotype is specifically revealed upon host chaperone depletion at 39°C, as we do not observe significant positive differential selection at these sites upon depleting chaperones at 37°C (Fig 1C and S7 Fig—HSF1i 37°C versus Basal 37°C) and observe only modest positive differential selection upon increasing the temperature in an environment with basal levels of chaperones (Fig 1C and S6 Fig—Basal 39°C versus Basal 37°C).

**Fig 2:**
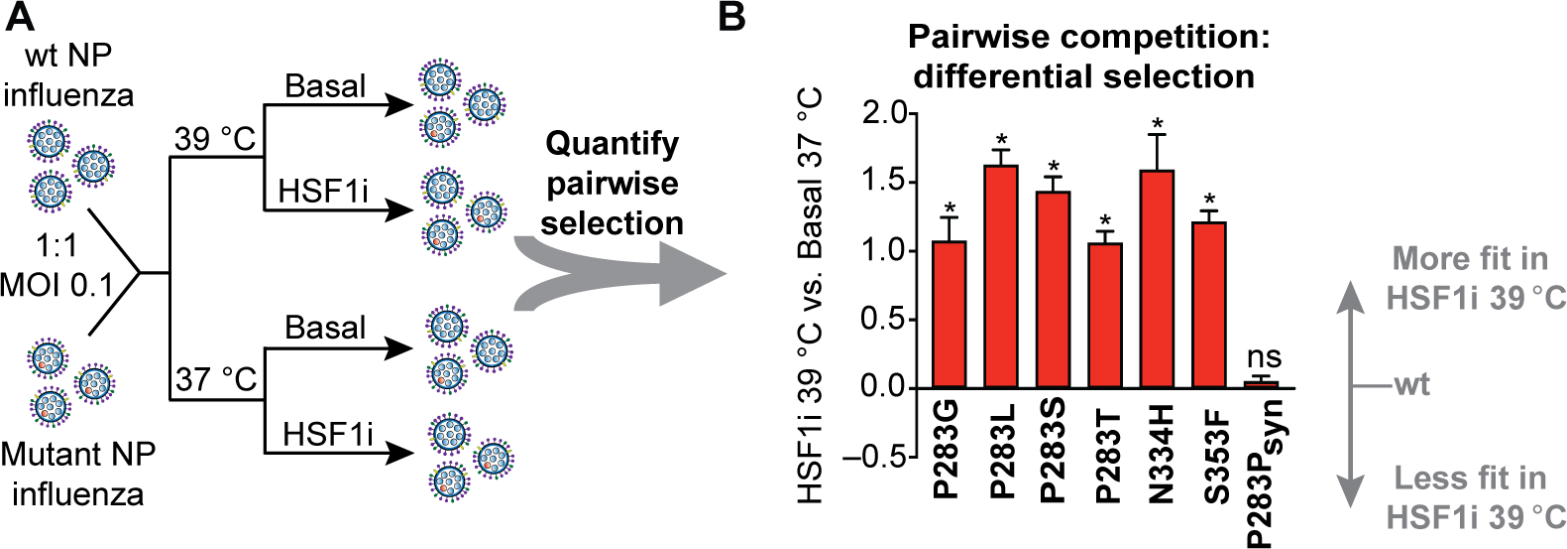
Pairwise competitions between highly selected variants and wt NP recapitulate the bulk competition results. (A) Pairwise viral competition scheme: wt NP influenza was mixed with mutant NP influenza in an approximately 1:1 ratio and was then subjected to selection during replication at 37 **°**C and 39 **°**C in both basal and chaperone-depleted (HSF1i) cells at an MOI of 0.1 virions/cell. The differential selection for each NP mutant was assessed by deep sequencing the viral competition mixture following selection. Mutation differential selection was evaluated as the logarithm of the enrichment of the corresponding variant. (B) Differential selection of NP variants upon chaperone depletion at 39 **°**C relative to basal chaperone levels at 37 **°**C during pairwise competitions. Error bars indicate the standard error from biological replicates (*N* = 3). The significance of deviation from zero (no selection) was evaluated by a one-sample *t* test followed by FDR adjustment of corresponding *p*-values; * indicates an FDR < 0.05. Differential selection values for pairwise competitions are provided in S5 Data. FDR, false discovery rate; HSF1i, condition in which heat shock factor 1 was inhibited; MOI, multiplicity of infection; NP, nucleoprotein; ns, not significant; syn, synonymous; wt, wild type.

Competitions between thousands of variants are often inherently noisy, largely owing to differences in variant composition between replicate libraries [27]. However, we observed strong correlation for selection on NP sites between biological (*R*^2^ = 0.71) and technical (*R*^2^ = 0.79) replicates of our deep mutational scanning experiments (S3E Fig and S3 and S4 Data). Thus, the selection strength imparted by host chaperone depletion at a restrictive, fever-like temperature substantially exceeds the experimental noise. Moreover, these deep mutational scanning results were recapitulated in all pairwise competitions we performed between individual highly selected variants and virus carrying the wild-type NP (Fig 2 and S8 Fig), and no significant fitness change was observed for a synonymous variant used as a control (Fig 2B, false discovery rate [FDR] = 0.4). Cumulatively, these findings confirm the validity of the deep mutational scanning data and indicate that depleting chaperones at a moderately elevated temperature creates a stringent host environment that strongly selects against wild-type residues in NP at certain sequence positions.

### Biophysical costs of individual NP variants

The strong differential selection we observed in chaperone-depleted host cells at a restrictive temperature suggested to us that specific wild-type NP residues in the Aichi influenza strain, the strain our libraries are based on, may entail a biophysical cost that is nonetheless tolerated under permissive conditions, perhaps owing to competing selection forces. This hypothesis is particularly compelling for the previously characterized stabilizing NP variants [10] N334H (Fig 1C and S4 Fig) and M136I (S4 Fig), which were positively selected in biophysically challenging conditions. These variants exhibited modestly enhanced fitness upon increased temperature and significantly enhanced fitness upon chaperone depletion at increased temperature. The unmasking of the deleterious fitness effects of the wild-type sequence at these positions upon host chaperone depletion supports the hypothesis that chaperones can indeed rescue biophysically deleterious NP variants.

We observed a similar and even more striking trend at site 283, where numerous amino acid substitutions—including serine (Ser), leucine (Leu), threonine, and glycine (Gly)—were validated in our pairwise competition experiments to be significantly more fit than the wild-type Pro residue when host chaperones were depleted at a restrictive temperature (Fig 1C and Fig 2B—HSF1i 39°C versus Basal 37°C). The identity of the amino acid at NP site 283 is known to critically modulate influenza sensitivity to the human antiviral restriction factor MxA, with Pro at that position contributing greatly to MxA escape [9, 16]. Although a structure of the MxA:NP complex is not currently available, site 283 is likely located at the MxA–NP interface [29]. Moreover, Pro283 is nearly universally conserved in human influenza NP but rarely occurs in avian influenza NP. This characteristic difference between human and avian influenza strains is attributed to the necessity of Pro283 to escape human MxA, whereas the avian MxA ortholog lacks known antiviral activity [30]. Indeed, the Leu283Pro substitution enabled the 1918 pandemic influenza strain to escape MxA following zoonotic transmission [16]. Pro283 in NP has thus greatly impacted the fitness of modern human influenza strains.

The observation that an adaptive amino acid substitution as important as Pro283 can be rendered unfit by biophysically challenging, host chaperone–depleted conditions motivated us to elucidate the underlying molecular basis of this phenomenon. Our hypothesis was that the depletion of host chaperones exacerbates a biophysical defect in NP folding that is caused by installation of a Pro at position 283. Prior work has shown that NP is engaged by numerous cytosolic chaperones, including chaperones like heat shock protein 40 (Hsp40) and heat shock protein 70 (Hsp70) that are depleted in our HSF1-inhibited host cell environment [31–35] (S1 and S2 Figs). For example, Hsp40 (*DNAJB1*) directly interacts with NP and facilitates its nuclear import [34], while Hsp70 is implicated in modulating NP nuclear export [35]. The heat shock proteins can also regulate antiviral responses indirectly through their interactions with NP [31–33]. Thus, the disruption of critical NP–host chaperone interactions by HSF1 inhibition-mediated chaperone depletion may indeed be the source of significant differential selection at site 283, especially at a biophysically restrictive temperature.

**Fig 3:**
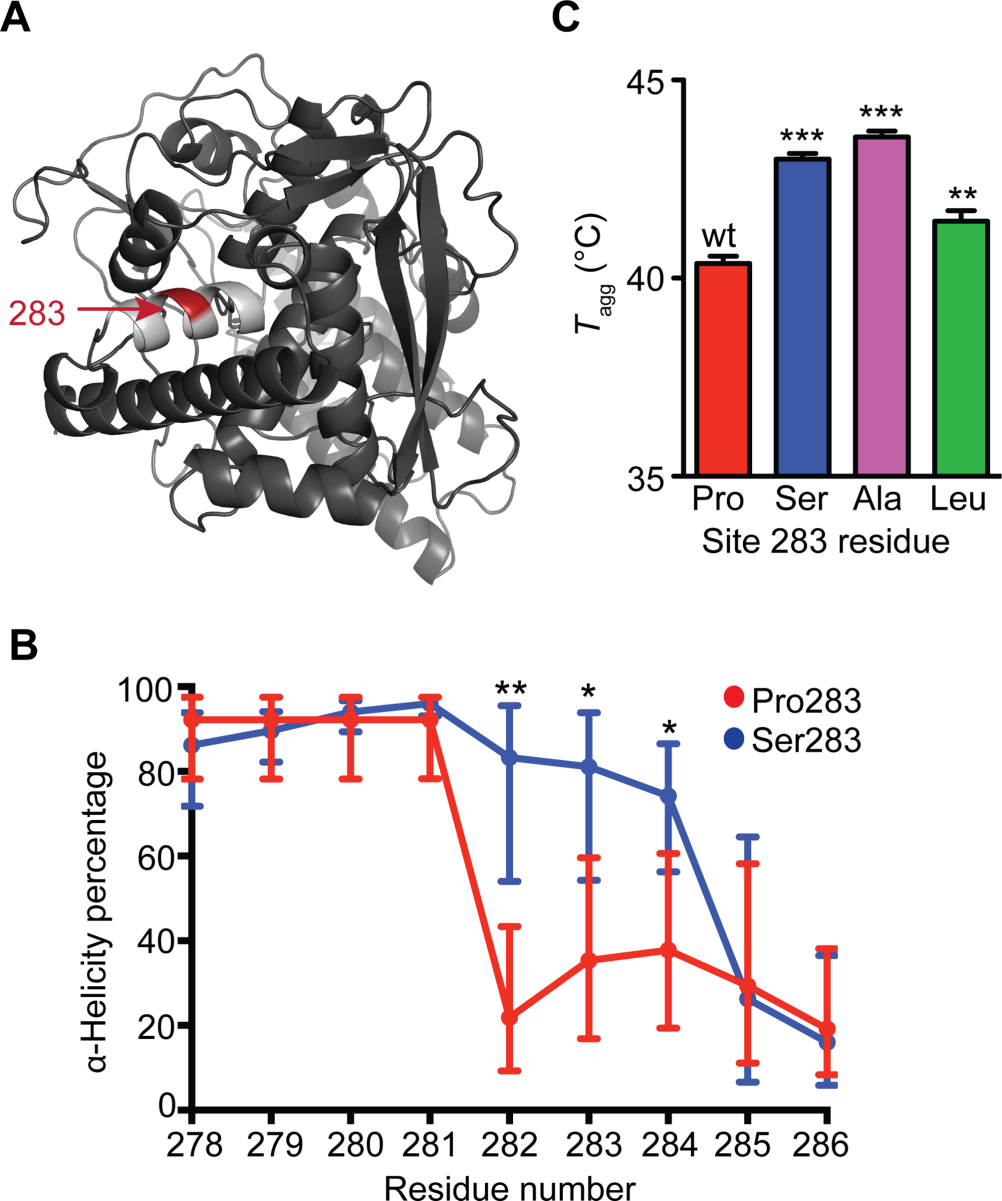
Pro283 disrupts nucleoprotein α-helical content and is destabilizing relative to other variants at site 283. (A) Nucleoprotein crystal structure reveals site 283 is in the center of an α-helix (PDB ID: 2IQH [36]). (B) Molecular dynamics simulations show that Pro283 disrupts nucleoprotein α-helical content compared to Ser283. A Student *t* test indicates significant differences at sites 282–284. Replicate α-helicity percentages are provided in S6 Data. (C) Apparent (irreversible) melting temperatures (*T*_agg_) of wild-type and site 283 nucleoprotein variants evaluated by circular dichroism. Student *t* test indicates the significance of deviation from wild-type apparent melting temperature. Replicate apparent melting temperatures are provided in S7 Data. Ala, alanine; Leu, leucine; PDB, Protein Data Bank; Pro, proline; Ser, serine.

This possibility raises the question of whether the Pro283 NP variant is, in fact, biophysically defective relative to other variants at site 283. Although there is currently no high-resolution structure of a NP variant containing Pro283, crystal structures of avian influenza NP variants with Ser or Leu at position 283 are available [36, 37]. In these structures, site 283 is in the middle of an α-helix (Fig 3A). Pro is classically regarded as a “helix-breaker,” owing in part to its inability to form an *i* + 4 hydrogen bond important for α-helix stability [38]. Therefore, it seemed reasonable to anticipate that the replacement of Ser (or Leu) with a Pro at position 283, as is observed in human influenza strains, would indeed be biophysically problematic.

To assess this hypothesis, we first performed molecular dynamics (MD) simulations in explicit water to evaluate whether Pro283 affects either the overall structure of NP or the structure of the α-helix centered at position 283. Although the overall NP structure was not grossly perturbed by Pro283, as would be expected given that this variant is still capable of supporting influenza replication, our simulations revealed that a Pro283 NP variant is significantly less α-helical at residues 282–284 than is a Ser283-containing variant (Fig 3B, S1 and S2 Videos and S6 Data).

The significant structural consequences of Pro283 observed in these simulations prompted us to experimentally investigate whether Pro283 affects NP stability. Following a previously reported protocol [10], we recombinantly expressed and purified a monomeric form of NP with either Pro, Ser, Leu, or alanine (Ala) at position 283 (S9A–S9C Fig). Circular dichroism spectra indicated that all four variants had grossly similar secondary structures (S9B Fig), consistent with our simulations. Thermal denaturation of NP is irreversible, leading to rapid aggregation and precipitation of the protein. Nonetheless, fitting these irreversible thermal melts to a two-state model revealed that Pro283 NP does indeed precipitate at a significantly lower temperature than all three of the non-Pro variants studied (Fig 3C, S9C Fig and S7 Data) and is therefore less stable and more aggregation prone.

The observation that Pro283 NP has a higher propensity to aggregate than other variants is consistent with either a kinetic or thermodynamic defect caused by substitution with Pro283. Given that Gly is, like Pro, known as an α-helix breaker and that Gly283 is positively selected relative to Pro283 (see Fig 1C—HSF1i 39°C versus Basal 37°C) upon chaperone depletion at febrile temperatures, we favor a substantial contribution from a kinetic defect that may be associated with the propensity of Pro to form both *cis*- and *trans*-amide bonds [38]. The substantive biophysical defect endowed by Pro283 on NP likely explains the enhanced dependence of this variant on host chaperones and explains why other variants are significantly more fit in the absence of those key chaperones. Moreover, these results may help to explain why Pro283 is not observed in avian influenza strains [16]. Birds typically have body temperatures ranging from 39–43°C [39], the upper end of which may be too extreme to permit chaperone-mediated rescue of the biophysically defective Pro283 NP variant.

### Evaluating competing selection pressures of MxA escape and host chaperone depletion

**Fig 4:**
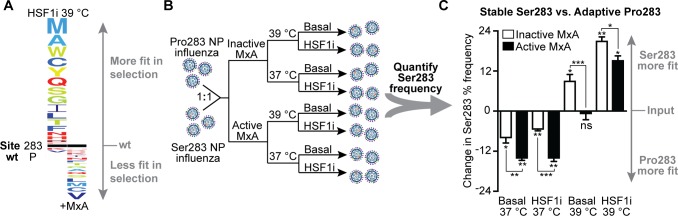
Host chaperones define the immune escape capacity of the Pro283 NP variant. (A) Deep mutational scanning reveals opposing selection forces from chaperone depletion versus MxA-mediated immune selection. Left: chaperone depletion at 39°C shown relative to the basal environment at 37°C, as in Fig 1C. Right: MxA selection from previously published deep mutational scanning data [9]. The size of the amino acid letters corresponds to the magnitude of the mutational differential selection, which is on the same scale for each selection. Amino acids above the black line are more fit than wt Pro283 in the selection condition, and amino acids below are less fit. (B) Pairwise viral competition scheme: wt NP influenza was mixed with mutant NP influenza in a 1:1 ratio. The mixture was then subjected to selection at 37°C and 39°C in both basal and chaperone-depleted (HSF1i) cells at an MOI of 0.1 virions/cell in the presence of either active or inactive MxA. (C) Change in mutant frequency upon selection is plotted on the y-axis, with the origin representing the mutant frequency in the input (inoculum). Error bars indicate the standard error from biological replicates (*N* = 3). The significance of deviation from input (no selection) was evaluated for each selection condition by a one-sample *t* test; * and ** above individual bars indicate FDR < 0.05 and < 0.01, respectively. Nested ANOVA accounting for replicates and treatment conditions was performed for all selections (*p* = 1.16 × 10^−9^). The significance of the difference in mutant frequency in the presence of active versus inactive MxA was evaluated for each selection condition by post hoc pairwise comparisons; *, **, and *** between the bars indicate *p*-values < 0.05, < 0.01, and < 0.001, respectively. All *p*-values from post hoc pairwise comparisons are provided in S9 Data. Replicate mutant frequencies are reported in S8 Data. ANOVA, analysis of variance; FDR, false discovery rate; HSF1i, condition in which heat shock factor 1 was inhibited; MOI, multiplicity of infection; MxA, Myxovirus resistance protein A; NP, nucleoprotein; ns, not significant; wt, wild type.

Cumulatively, these results suggest that NP variants critical for innate immune system escape, most especially Pro283, can be folding-defective and display compromised fitness in biophysically challenging host environments (Fig 4A, left). This finding motivated us to evaluate whether Pro283 fitness remains compromised under biophysically challenging conditions even upon the addition of an MxA selection pressure that normally selects strongly in favor of the Pro283 variant (Fig 4A, right). To this end, we performed pairwise viral competitions between the biophysically stable Ser283 variant and the MxA-resistant Pro283 variant in each of our host environments in the presence of either active or inactive MxA (Fig 4B and S2E Fig). In permissive folding environments (Basal and HSF1i at 37°C), Pro283 was enriched compared to Ser283, thereby enabling MxA escape (Fig 4C). In contrast, in biophysically challenging environments (Basal and HSF1i at 39°C), the stability defects of Pro283 were exacerbated, and Ser283 was enriched compared to Pro283. Ser283 was enriched even in the presence of MxA selection pressure when chaperones are depleted at 39°C, thereby hindering immune escape.

**Fig 5:**
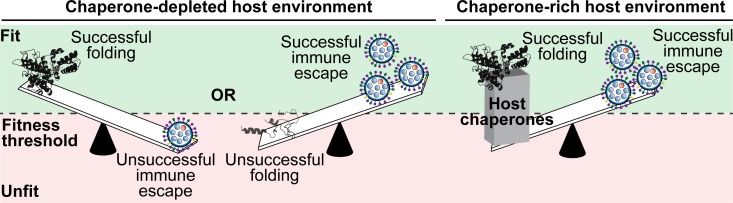
Host chaperones mediate the accessibility of biophysically destabilized adaptive mutations. (Left) Model for a chaperone-depleted environment, in which immune escape and acceptable protein biophysical properties present strongly competing selection pressures. (Right) Model for a chaperone-rich host environment, in which, at least for some viral protein variants, these pressures can be uncoupled as chaperones enable viral proteins to maintain acceptable folding and structural properties while still endowing escape from the immune system.

Altogether, these data reveal that HSF1-regulated chaperones can define the fitness of biophysically destabilized immune escape variants in influenza. This observation suggests a model in which enhanced fitness conferred by immune escape is often counterbalanced over the course of influenza evolution by biophysical defects that have a substantive fitness cost (Fig 5, left). Remarkably, at least in the case of influenza NP, our data show that the virus is able to hijack host chaperones to resolve these biophysical defects (Fig 5, right). By this mechanism, the virus manages to uncouple protein folding fitness costs from the advantageous consequences of immune escape, expanding the accessible mutational landscape to access essential viral protein variants capable of both folding and immune escape.

## Discussion

This work provides, to the best of our knowledge, the first direct experimental evidence that host chaperones mediate the accessibility of biophysically deleterious, adaptive viral protein variants. This feature of the host–pathogen interaction is apparent in multiple sites across the NP gene. Particularly noteworthy, we find that the destabilized Pro283 NP variant is not tolerated in a chaperone-depleted host environment at a restrictive temperature, as NP is unable to engage host chaperones to address the Pro283-induced biophysical defect. Remarkably, we observe that even in the presence of selection pressure imposed by MxA that strongly favors Pro283 [9], the fitness of Pro283 NP is still contingent on the host’s chaperone levels and biophysical environment.

Based on these results, we expect that host chaperones can impact the accessibility of adaptive viral protein variants far beyond NP and influenza, as amino acid substitutions are largely destabilizing [6], and many viral proteins are known to engage host chaperones [34, 40, 41]. Moreover, previous work has revealed (1) that viral evolution is fundamentally constrained by protein stability [4, 10] and (2) the role of the Hsp90 chaperone in viral replication across numerous viral families [41–44]. Thus, the evolutionary trajectories of diverse viral proteins are likely to be influenced by numerous host chaperones [18, 19].

Since our work suggests that host chaperones preferentially rescue biophysically defective viral protein variants, as more data accumulate in this field, we may eventually be able to predict how chaperones will impact fitness in a rational manner based on protein variant biophysical properties. Further, our infections in the presence of the MxA restriction factor demonstrate that host chaperones can mediate the accessibility of escape variants irrespective of competing selection pressures. These data raise the possibility of antiviral therapeutic adjuvants targeting host chaperones that inhibit the development of antiviral resistance by constraining the accessible mutational landscape. We further observe that temperature critically influences the fitness of viral variants, with most variants suffering fitness costs at elevated temperatures that mimic fever conditions and/or the body temperatures of birds and small mammals [39]. Thus, fever and host-switching events may impose selection on viral variants that hampers adaptation. Based on our findings here, the nature of such selection is likely to be strongly influenced by host-specific differences in chaperone network compositions.

We anticipate that these phenomena extend far beyond the host–pathogen interface and apply to protein evolution more broadly. Previous work by Lindquist and others suggested that the Hsp90 chaperone can potentiate and buffer genetic variation in endogenous proteins [45–49]. Here, the impact of the host chaperone environment on NP variant fitness is driven predominantly not by Hsp90 (see S10 Fig), even though NP does engage this chaperone [50], but instead by inhibition of HSF1 modulating the composition of a complex network of multiple protein folding and quality control factors. Moreover, our work shows experimentally that chaperones have the largest effect on the fitness of biophysically defective protein variants, a result that may help to explain extensive prior work with Hsp90 in which the potential biophysical mechanism of effects on protein evolution have not been experimentally evaluated.

HSF1-regulated host proteostasis network components may modulate NP evolution directly—for example, by impacting NP–chaperone interactions—or indirectly, perhaps by perturbing levels of endogenous chaperone client proteins with antiviral properties. Our data support the former case, as we find that destabilized NP variants are particularly sensitive to chaperone depletion. Nonetheless, we would not rule out possible contributions from secondary effects. Deciphering between primary and secondary effects will first require identifying the individual components of the intricate protein folding network that are primarily responsible for modulating NP fitness, followed by systematic elimination of potential downstream effectors. Whether primary or secondary, the evolutionary implications of the protein folding network clearly extend well beyond Hsp90 and also play critical roles in evolution at the host–pathogen interface.

Finally, this work provides experimental evidence for the longstanding hypothesis that chaperones buffer the fitness cost of biophysically destabilized protein variants [45, 51, 52]. Experimental validation of this concept in metazoan cells for the first time, to the best of our knowledge, has significant consequences for understanding the constraints on protein evolution, which have so far focused on inherent biophysical properties of proteins [2, 10]. For specific destabilized adaptive variants, fitness has been attributed to compensatory stabilizing mutations elsewhere in the protein structure [10, 53]. Cases of this idiosyncratic epistasis mediating pathogen adaptation have motivated efforts to determine the pervasiveness of compensatory mutations [10]. Our data reveal that the constraints on protein evolution are still more complex, establishing that protein variant fitness is constrained not just by inherent stability but also by the cellular environment in which the protein folds.

## Materials and methods

### Plasmids

The following plasmids were used to generate the A/Aichi/2/1968 influenza virus: pHWAichi68-NP [54], pHWNan95-PB2 [54], pHWNan95-PB1 [54], pHWNan95-PA [54], pHW184-HA [55], pHW186-NA [55], pHW187-M [55], and pHW188-NS [55]. For NP recombinant expression and biophysical studies, a pET28b(+) expression vector encoding monomeric A/Aichi/2/1968 NP with an R416A mutation (which prevents RNA binding), deletion of residues 2–7, and a C-terminal 6×-His tag was used [10]. A lentiviral vector containing a FLAG-tagged human MxA or inactive MxA(T103A) sequence under a CMV promoter with a GSG linker (DYKDDDDKGSG) at the C-terminus was used for generation of the MxA-expressing MDCK cell line [9]. Downstream of MxA, the plasmid contained an internal ribosome entry site (IRES) followed by an mCherry reporter gene to assist the selection of stable single-colony cell lines.

### Antibodies

Antibodies used were as follows: rat monoclonal anti-FLAG (Agilent; 200474), mouse monoclonal anti-β-actin (Sigma; A1978), rabbit polyclonal anti-Hsp90, rat monoclonal anti-Hsp70, and rabbit monoclonal anti-Hsp40 from Cell Signaling (4877, 4873, and 4871, respectively). IRDyes 800CW goat anti-rat, 680LT goat anti-mouse, and 800CW goat anti-rabbit secondary antibodies were obtained from LI-COR (926–32219, 926–68020, and 926–32211, respectively).

### Cell lines

For the MDCK^dn-cHSF1^ cell line construction, MDCK cells were originally purchased from the American Type Culture Collection (Manassas, VA, United States) and validated as MDCKs by STR profiling (Science Exchange). Cells were cultured at 37°C in a 5% CO_2_(g) atmosphere in DMEM (CellGro) supplemented with 10% fetal bovine serum (FBS; CellGro) and 1% penicillin/streptomycin/glutamine (CellGro). The parental MDCK cells were transduced first with lentivirus encoding a blasticidin-resistant tetracycline repressor and then with lentivirus encoding a zeocin-resistant, tetracycline-inducible dn-cHSF1 construct [23]. Heterostable cells expressing the tetracycline repressor and the dn-cHSF1 construct were then selected using 8 μg/mL zeocin and 4 μg/mL blasticidin. Single colonies were generated by serial dilution in 96-well plates, expanded, and then selected based on functional testing of HSF1 inhibition [23] using qPCR (S2B Fig), as previously described.

For MDCK^dn-cHSF1-MxA^ and MDCK^dn-cHSF1-MxA(T103A)^ cell line construction, MDCK^dn-cHSF1^ cells were engineered to constitutively express human MxA or the inactive MxA-T103A mutant [9] by transducing with lentivirus encoding FLAG-tagged MxA variants. At 72 h post transduction, single colonies were generated by serial dilution in 96-well plates. Wells with clonal transduced cells were identified as single clusters of cells expressing mCherry, expanded, and then characterized as described below.

### Characterization of cellular environments for influenza competitions

#### Quantitative RT-PCR

MDCK^dn-cHSF1^ cells were plated in 12-well plates at a density of 100,000 cells/well and pretreated with 0.1% DMSO or doxycycline at a final concentration of 1 μg/mL upon seeding. After 18 h, the cells were infected with wild-type influenza A/Aichi/2/1968 at an MOI of 1 virion/cell. Infectious media were replaced with fresh WSN media (OptiMEM-I from Thermo Fisher Scientific supplemented with 0.5% heat-inactivated FBS, 0.3% BSA from Invitrogen, 1% of penicillin/streptomycin from Bio Whittaker, and 100 μg/mL of CaCl_2_ from Sigma) supplemented with 0.1% DMSO or 1 μg/mL doxycycline 2 h post infection. Infected cells were treated with 100 μM sodium arsenite (Alfa Aesar) for 90 min prior to harvesting for a heat shock activation control and harvested 8 h post infection. Cellular RNA was extracted using Total RNA Kit I with Homogenizer columns (Omega). RNA (1 μg) was reverse transcribed into cDNA using the Applied Biosystems High-Capacity Reverse Transcription kit. The reverse transcription reaction (20 μL) was diluted to 80 μL with molecular biology–grade water, and 2 μL of each sample was used for qPCR with the 2× Sybr Green Reaction Mix (Roche) and primers for *canis RPLP2* (housekeeping gene), *HSP90AA1* (Hsp90), *HSPA1A* (Hsp70), *DNAJB1* (Hsp40), and influenza matrix protein (primer sequences are provided in S1 Table). Transcript levels of heat shock proteins were normalized to *RPLP2*, and normalized transcript levels for each treated condition were quantified relative to the basal environment at 37°C (S2B Fig). For a positive control of productive infection, matrix protein transcript levels were compared with a prepared standard curve using the pHW187-M plasmid.

#### RNA extraction and sequencing

MDCK^dn-cHSF1^ cells were seeded at 100,000 cells/well in a 12-well plate and treated with 0.1% DMSO or 1 μg/mL doxycycline for 48 h. Each treatment was performed in biological triplicate. Cellular RNA was harvested using the RNeasy Plus Mini Kit with QIAshredder homogenization columns (Qiagen). RNA-seq libraries were prepared using the Kapa mRNA HyperPrep RNA-seq library construction kit (Kapa/Roche), with fragmentation times of 7 and 6 min at 94°C for the 37°C and 39°C samples, respectively, and final amplifications of 15 and 12 PCR cycles, respectively. The resulting libraries were sequenced on an Illumina HiSeq using 40-bp single-end reads. RNA-seq quality control and differential expression analysis was performed as previously described [18]. Briefly, reads were aligned against the *Canis familiaris* genome assembly canFam3 with an ensembl annotation using STAR v. 2.5.3a. Gene expression was quantified using RSEM v. 1.3.0. Differential expression analysis to compare conditions (S1 Fig and S1 Data) was performed using DESeq2 version 1.10.1 running under R version 3.2.3.

#### Resazurin cell growth assays

MDCK^dn-cHSF1^ cells were seeded at 100,000 cells/well in 12-well plates and pretreated with 0.1% DMSO or 1 μg/mL doxycycline. To mimic the infection conditions, we replaced the cellular growth media with WSN media and supplemented with 0.1% DMSO or 1 μg/mL doxycycline 18 h after seeding. At 48 h after the mock infection, the media were replaced with WSN media containing 50 μM resazurin sodium salt (Sigma). After 4 h of incubation, 100 μL of media was used to quantify resorufin fluorescence (excitation 530 nm; emission 590 nm) using a Take-3 plate reader (BioTeK) (S2D Fig).

#### Immunoblot analyses

MDCK^dn-cHSF1^ cells were seeded at 200,000 cells/well in 6-well plates and treated with 1 μg/mL doxycycline or left untreated for 48 h. At 8 h prior to harvesting, cells were treated with 100 μM sodium arsenite for 2 h and then allowed to recover in fresh media for 6 h. For protein levels examined during influenza infection, MDCK^dn-cHSF1^ cells were seeded at 200,000 cells/well in 6-well plates, pretreated with 0.1% DMSO or 1 μg/mL doxycycline for 24 h, and then infected with wild-type influenza A/Aichi/2/1968 at an MOI of 1 for 8 h. Cells were then harvested, washed 3× with PBS, and lysed (1% Triton, 200 mM NaCl, 50 mM Tris at pH 7.5, 1 mM EDTA, 1.5 mM MgCl_2_, and protease inhibitor tablet from Thermo Fisher Scientific). After clearing the lysate by centrifugation at 21,100 × g for 15 min at 4°C, 100 μg of total protein lysate was separated on a 12% SDS-PAGE gel and transferred to a nitrocellulose membrane using the Trans-Blot Turbo system (Bio-Rad). Membranes were then probed for Hsp90, Hsp70, Hsp40, and β-actin, as indicated, and imaged using an Odyssey Infrared Imaging System (Li-Cor) (S2A Fig). To verify that MDCK^dn-cHSF1-MxA^ and MDCK^dn-cHSF1-MxA(T103A)^ cell lines expressed FLAG-MxA and FLAG-MxA(T103A), respectively, lysate from biological duplicate samples was separated on a 12% SDS-PAGE gel as described above and immunoblotted for the FLAG epitope and β-actin (S2E Fig; representative blot shown).

### Deep mutational scanning

Two independent plasmid mutant libraries previously generated [24] from the pHWAichi68-NP template plasmid were used to create mutant viral libraries by transfecting a coculture of 25,000 MDCK-SIAT1 and 300,000 HEK 293T cells, as previously described [24]. For each library, cocultures in eight 6-well plates were transfected to maintain library diversity, and transfection supernatants were combined to generate the input mutant viral libraries. Viruses were generated and grown in WSN media. Infectious titers of viral libraries were determined by a tissue culture infectious dose (TCID_50_) assay. Briefly, 10-fold serial dilutions of viruses (in technical triplicates) were prepared in 96-well plates and incubated with 5 × 10^3^ MDCK-SIAT1 cells/well for 72 h at 37°C. The wells were then scored for cytopathic effects, and viral titers were calculated using a Reed-Muench Calculator, available at https://github.com/jbloomlab/reedmuenchcalculator.

Two plasmid libraries were used to generate two biological replicate viral libraries, one of which was used twice to perform 2 technical replicates of the deep mutational scanning (S3A Fig). MDCK^dn-cHSF1^ cells were plated in 15-cm plates at a density of 6 × 10^6^ cells/dish and treated with 0.1% DMSO or 1 μg/ml doxycycline for 24 h at either 37°C or 39°C. Deep mutational scanning was also performed in cells treated with an Hsp90 inhibitor (100 nM 17-AAG; 90 min pretreatment). Then, 5 × 10^6^ infectious virions (as determined by a TCID_50_ assay) from each viral library were used to infect four 15-cm plates from each condition at an MOI of 0.1 virions/cell (the cells expanded to approximately 12.5 × 10^6^ cells per plate by the time of infection). In addition, one 15-cm plate at both 37°C and 39°C was either mock-infected (negative control) or infected with wild-type virus. For infection, the cellular growth media were replaced with WSN media containing a mutant virus library, wild-type virus, or no virus for mock infection. After 2 h, the inoculum was replaced with fresh WSN media containing 0.1% DMSO, 1 μg/mL doxycycline, or 100 nM 17-AAG. At 48 h post infection, the viral supernatant was harvested, centrifuged at 1,000 × g for 5 min to remove cell debris, and stored at −80°C. Viral RNA was extracted from the infectious supernatant using a Viral RNA Mini Kit (Qiagen) and reverse transcribed using the AccuScript High Fidelity 1st Strand cDNA Synthesis Kit (Agilent) using 5′-BsmBI-Aichi68-NP and 3′-BsmBI-Aichi68-NP primers (primer sequences in S1 Table). At least 10^6^ NP molecules were PCR-amplified for preparation of the sequencing libraries, as previously described [27] (S3B Fig). The resulting amplicons were sequenced on an Illumina HiSeq 2500 in rapid run mode with 250-bp paired-end reads (S3C and S3D Fig). dms_tools (http://jbloomlab.github.io/dms_tools/) [56] was used to align reads to the Aichi NP reference sequence, count amino acid variants across NP, and calculate the differential selection for each variant between 2 selection conditions, as previously described [9, 28] (S3 and S4 Data).

### Pairwise viral competitions

NP variants that reproducibly exhibited the most positive or negative differential selection in the deep mutational scan were selected for pairwise competitions (P283M, P283A, P283G, P283L, P283S, P283T, S353L, S353F, N334H, D34N, H82N, and a synonymous control P283P). The NP mutants were generated by introducing point mutations into the pHWAichi68-NP plasmid using the QuikChange II XL Site-Directed Mutagenesis Kit (Agilent). Technical difficulties with specific site-directed mutagenesis reactions prevented generation of the P283M, P283A, and S353L mutant plasmids. The remaining 9 mutant plasmids were generated successfully and used to produce the corresponding mutant viruses by transfecting a coculture of 25,000 MDCK-SIAT1 and 300,000 HEK 293T cells, as previously described [55]. The resultant viruses were titered using a TCID_50_ assay. For each competition, 100,000 cells/well of MDCK^dn-cHSF1^, MDCK^dn-cHSF1-FLAG-MxA^, or MDCK^dn-cHSF1-FLAG-MxA(T103A)^ cells were plated in 12-well dishes and treated with 0.1% DMSO or 1 μg/mL doxycycline for 18 h at either 37°C or 39°C. Cells were infected with a 1:1 mixture of wild-type and mutant viruses at an MOI of 0.1 virions/cell in triplicate under conditions identical to that of the deep mutational scanning experiment. After 2 h, the inoculum was replaced with fresh WSN media containing either 0.1% DMSO or 1 μg/mL doxycycline. At 48 h post infection, infectious supernatants were harvested, centrifuged at 1000 × g for 5 min to remove cell debris, and stored at −80°C. Viral RNA was extracted from the infectious supernatant using the QIAamp Viral RNA Mini Kit, and at least 10^6^ NP molecules were reverse transcribed using the SuperScript III Reverse Transcriptase (Thermo Fisher Scientific) with 5′-BsmBI-Aichi68-NP and 3′-BsmBI-Aichi68-NP primers (S1 Table). The amplicons were visualized on a 1% analytical agarose gel to verify amplification of the NP gene (1.5 kb). The dsDNA was purified using 1.5× AMPure XP beads (Beckman Coulter) and quantified using a Quant-iT PicoGreen Assay (Life Technologies). Illumina NexteraXT sequencing libraries were prepared using a Mosquito HTS Liquid Handler (TTP Labtech) and sequenced on an Illumina MiSeqv2 in 2 runs of either 40-bp single-end or 150-bp paired-end reads. To call sequence variants, reads were aligned to the Aichi NP reference sequence using bwa mem (v. 0.7.12-r1039) (arXiv:1303.3997v2) with flag–t 16, and sorted and indexed bam files were generated using samtools (v 1.3) [57]. These bam files were processed using samtools mpileup with flags–excl-flags 2052, -d 30000000, and the same Aichi NP reference sequence used for mapping [58]. For pairwise competitions in the absence of MxA, mutant allele frequencies were normalized to wild-type allele frequencies for each sample, and the resulting values were used to calculate the differential selection [28] (Fig 2, S8 Fig, and S5 Data). For pairwise competitions in the presence of wild-type or inactive MxA, the change in mutant allele frequencies is reported (Fig 4C and S8 Data).

### MD simulations

Two sets of simulations were performed for NPs with the sequence of the human H3N2 variant (Fig 3A and 3B). In one set, NP residue 283 was Pro (this system is termed Pro283 hereafter). In the other set, residue 283 was Ser (this system is termed Ser283 hereafter). The initial structures of both systems were prepared using the comparative modeling software RosettaCM [59], with the structures of H1N1 influenza A virus NP (PDB ID: 2IQH [36]) and H5N1 NP (PDB ID: 2Q06 [37]) used as templates with equal weights. The first 20 amino acids in the N-terminal region, whose 3D coordinates are missing in the template structures, were considered flexible and also of minimal impact to the region near residue 283. Therefore, they were removed in the following simulation.

In the threading procedure, the target NP sequence was aligned with the templates [60] and assigned coordinates from the template PDB structures (2IQH and 2Q06). The helix formed by residues 278–286 of 2IQH and 2Q06 was removed to allow RosettaCM to construct this region without influence from the templates. This region (residues 278–286), along with all the other regions missing in the template PDBs, was patched in the hybridization step. During hybridization, RosettaCM generated hybridized structures that contained pieces from each of the threaded structures, providing more accurate comparative models that were energetically favorable. Additionally, RosettaCM used fragments and minor ab initio folding to fill in residues not previously aligned with any template sequences during the threading process. A total of 1,000 models for each NP system were created, and the best-scoring model without a disulfide bond was used as the initial structure for further MD simulations.

Five runs of MD simulations for each NP system were carried out using GROMACS with the oplsaa/tip4p force field [61, 62]. The N-terminus of the initial structure of the MD simulation was capped with an acetyl group, while the C-terminus was free (ending with COO^–^). This structure was energy minimized in a vacuum and then immersed in the center of a cubic box containing preequilibrated water molecules with an edge of 12 nm. The system was electrostatically neutralized by adding 11 Cl^−^ions. The solvated system was further energy minimized to remove any bad contacts. The solvated NP then underwent 2 stages of equilibrations. The first stage of equilibration consisted of a 50-ps isochoric–isothermal (NVT) simulation at 300 K and a subsequent 50-ps isobaric–isothermal (NPT) simulation at 300 K and 1 bar. During the first stage of equilibration, the NP-heavy atoms were restrained by a harmonic potential with a force constant of 1,000 kJ mol^–1^ nm^–2^ to equilibrate the solvent molecules and adjust the density. The second stage of equilibration consisted of an additional 100-ps NVT simulation at 300 K without any restraints to equilibrate the whole system, followed by a 100-ns NPT production simulation at 300 K and 1 bar. The V-rescale thermostat was coupled to both the NP and solvent separately, with coupling time constants of 0.1 ps. The pressure was maintained using the Parrinello-Rahman barostat with a coupling time constant of 2.0 ps and isothermal compressibility of 4.5 × 10^−5^ bar^-1^. The leapfrog algorithm with a time step of 2 fs was used for dynamics evolution. All bonds involving hydrogen were constrained using the LINCS algorithm. All neighbor searching, electrostatic interactions, and van der Waals interactions were truncated at 1.0 nm. Electrostatics were treated using the particle mesh Ewald (PME) summation with a Fourier spacing of 0.12 nm and an order of 4. A long-range dispersion correction for energy and pressure was applied to account for the 1.0-nm cutoff of Lennard-Jones interactions. Five 100-ns trajectories were produced for the Pro283 and Ser283 systems, respectively. The trajectories between 50 ns and 100 ns were used for analysis (S6 Data).

### Recombinant expression and biophysical characterization of NP variants

The P283S, P283A, and P283L amino acid substitutions were introduced into the wild-type influenza A/Aichi/2/1968 NP in a pET28b(+) expression vector using the QuikChange II XL Site-Directed Mutagenesis Kit (Agilent). This NP construct contained an R416A mutation and deletion of residues 2–7 to obtain nonaggregated, RNA-free NP in a CD-compatible buffer, as previously described [10]. Mutagenized plasmid DNA was isolated using the E.Z.N.A. Plasmid Mini Kit I (Omega). For bacterial expression, BL21(DE3) chemically competent cells were transformed with 1 μL of purified plasmid and incubated overnight on LB-kanamycin agar plates. Colonies were used to inoculate 50-mL LB-kanamycin cultures overnight. Then, 10 mL of starter cultures were used to inoculate 1 L LB-kanamycin cultures, which were shaken at 37°C until reaching an OD_600_ of 0.3–0.6. Cultures were then chilled on ice and induced with 500 μM IPTG (Sigma) overnight at 20°C. Cells were then pelleted, Dounce homogenized, and lysed by sonication in 50 mL of lysis buffer (50 mM sodium phosphate at pH 8.0, 500 mM NaCl, 0.5% Triton X-100, 10 mM imidazole, 1 mM PMSF, and 0.1 mg/mL MgCl_2_). Cells were sonicated for 2 min (30% amplitude, 10 s on, 10 s off; Branson Digital Sonifier). Lysates were then clarified for 30 min at 10,000 × g at 4°C. Clarified lysates were passed through 0.45-μm filters. His-tagged NP variants were then incubated on Ni-NTA (Millipore) columns for 60 min at 4 **°**C and washed with Ni-NTA Wash Buffer (50 mM sodium phosphate at pH 8.0, 300 mM NaCl, and 20 mM imidazole). Proteins were eluted using Ni-NTA Elution Buffer (50 mM sodium phosphate at pH 8.0, 300 mM NaCl, and 250 mM imidazole). Eluates were dialyzed overnight into analysis buffer (20 mM sodium phosphate at pH 7.0 with 300 mM NaF) using 3.5-kDa molecular mass cutoff SnakeSkin dialysis tubing (Fisher Thermo Scientific). Dialyzed proteins were concentrated using Amicon Ultra 3K MWCO filters (Millipore) and further purified over a size exclusion column (Bio-Rad ENrich SEC 650) (S9A Fig).

For circular dichroism analysis (S9B Fig), proteins were diluted to 5 μM in analysis buffer (quantified by A280 with a BioTek-Take3 micro-volume plate using a molar extinction coefficient of 56,600 M^–1^ cm^–1^). Thermal melts (S9C Fig) were performed at a scan rate of 2 **°**C per min, maintaining each temperature for 5 min before measurement of ellipticity at 209 nm. *T*_agg_ values were obtained, as the thermal denaturation of NP was irreversible and resulted in aggregation and precipitation (Fig 3C and S9C Fig; S7 Data). All circular dichroism analyses were performed on a Jasco J-1500 Circular Dichroism Spectrophotometer with a 1-mm QS quartz cuvette (Hellma).

### Statistical analyses

Deep mutational scanning was performed in biological duplicate with 2 technical replicates of one of the biological replicates (S3A Fig). MxA and MxA(T103A) protein expression in MDCK^dn-cHSF1-FLAG-MxA^ and MDCK^dn-cHSF1-FLAG-MxA(T103A)^ cells, respectively, were evaluated in biological duplicate (S2E Fig). All other experiments were performed in biological triplicate, with replicates being independent experimental entireties (i.e., from plating the cells to acquiring the data). Correlation between deep mutational scanning replicates was determined by linear regression using GraphPad Prism software, reporting *R*^*2*^ (S3E Fig). Site differential selection values from deep mutational scanning (Fig 1B) were tested for significance of deviation from zero (wild-type behavior) using a one-sample *t* test in GraphPad Prism. The raw *p*-values were adjusted for multiple comparison using the Benjamini-Hochberg procedure [63], setting an acceptable FDR at 0.05 (p.adjust function in R). Analysis of variance (ANOVA) was performed on all differential selection values across selections normalized to the Basal 37 **°**C condition using a nested ANOVA framework (accounting for replicates), modeling treatment/temperature as a fixed effect and the replicate as a random effect (lme function/RMLE in the R statistical environment, followed by ANOVA computation anova.lme with sequential adjustment). Post hoc pairwise comparisons were performed by general linear hypotheses testing using Tukey's method (as implemented in glht, in the multcomp R package) comparing all the means with single-step adjustment for multiple comparison. The *p*-values for pairwise comparisons against HSF1i 37°C versus Basal 37°C were 6.148 × 10^−6^ for Basal 39°C versus Basal 37°C and 9.196 × 10^−8^ for HSF1i 39°C versus Basal 37°C and 0.71 for comparison between HSF1i 39°C versus Basal 37°C and Basal 39°C versus Basal 37°C (Fig 1B). Differential selection values from pairwise competitions (Fig 2B and S8 Fig) were tested for significance of deviation from zero (wild-type behavior) using a one-sample *t* test in GraphPad Prism with FDR correction (S8 Fig). For pairwise competitions in the presence of MxA, the significance of deviation from the input Ser283 frequency was determined using a one-sample *t* test in GraphPad Prism followed by FDR correction; Basal 37 **°**C Inactive MxA (*t* = 5.916, *df* = 2); Basal 37°C Active MxA (*t* = 29.01, *df* = 2); HSF1i 37°C Inactive MxA (*t* = 18.24, *df* = 2); HSF1i 37 **°**C Active MxA (*t* = 17.91, *df* = 2); Basal 39°C Inactive MxA (*t* = 4.924, *df* = 2); Basal 39 **°**C Active MxA (*t* = 0.5877, *df* = 2); HSF1i 39°C Inactive MxA (*t* = 18.98, *df* = 2); HSF1i 39 **°**C Active MxA (*t* = 12.29, *df* = 2). ANOVA was performed for competitions in presence of MxA as described above; individual *p*-values for all pairwise comparisons using Tukey all-pair comparisons method are provided in S9 Data. For RNA-seq, log2 fold changes, *p*-values, and Benjamini-Hochberg-adjusted *p*-values (ADP) are reported for all expressed protein-coding genes in S1 Data. For MD simulations, each of the five 100-ns simulations was considered an independent replicate, and the %-time spent in an α-helical conformation was transformed using ln(*P*/(1–*P*)) before *t* tests were conducted to satisfy the prerequisite assumptions of normality (Fig 3B); 278 (*t* = 0.95655, *df* = 7); 279 (*t* = 0.52331, *df* = 6); 280 (*t* = −0.46542, *df* = 6); 281 (*t* = −1.21825, *df* = 6); 282 (*t* = −3.64906, *df* = 7); 283 (*t* = −2.83812, *df* = 8); 284 (*t* = −2.83606, *df* = 8); 285 (*t* = 0.17240, *df* = 7); 286 (*t* = 0.34125, *df* = 8). For apparent melting temperatures determined by circular dichroism, melt curves were performed in biological triplicate, the average and SEM are reported, and the significance of deviation from wild type was evaluated by a Student *t* test; Ser (*t* = 19.59, *df* = 4); Ala (*t* = 23.94, *df* = 4); Leu (*t* = 5.644, *df* = 4).

## Supporting information

S1 Fig Transcriptional profiles of modulated host environments. (A) Volcano plot of RNA-seq data for HSF1 inhibition at 37 **°**C. (B) Volcano plot of RNA-seq data for HSF1 inhibition at 39 **°**C. (C) Volcano plot of RNA-seq data for 39 **°**C relative to 37 **°**C in basal environment. (D) Volcano plot of RNA-seq data for HSF1-inhibited environment at 39 **°**C relative to a basal environment at 37 **°**C. For A–D, transcripts with >2-fold change and *p*-values < 10^−5^ are shown in red, with outliers labeled. The complete RNA-seq differential expression analysis is provided in S1 Data. HSF1, heat shock factor 1; RNA-seq, RNA sequencing.
(TIF)

S2 Fig HSF1 inhibition is effective during influenza infection and does not significantly perturb influenza propagation or host cell metabolic activity. (A) Protein levels of heat shock protein chaperones in basal and chaperone-depleted host cells at 37°C and 39°C, in the absence or presence of influenza. Representative western blots on the left; quantitation of biological triplicates on the right. Arsenite (As(III)) is a chemical stressor that induces the heat shock response. (B) Transcript expression in selection conditions during influenza infection. (C) Infectious titers determined by TCID_50_ for wild-type A/Aichi/2/1968 (H3N2) in selection conditions. (D) Metabolic activity of MDCK^dn-cHSF1^ cells in each selection condition, as characterized by resazurin assays at 48 hours post treatment. (E) Protein levels of FLAG-MxA and FLAG-MxA(T103A) in MDCK^dn-cHSF1-MxA^ and MDCK^dn-cHSF1-MxA(T103A)^ cells, respectively. MDCK^dn-cHSF1^ is shown as a negative control. Representative blot is shown (*N* = 2). For A–D, replicate data are provided in S2 Data. HSF1, heat shock factor 1; MDCK, Madin Darby canine kidney; TCID_50_, tissue culture infectious dose.
(TIF)

S3 Fig Subamplicon sequencing enables quantification of variant frequency. (A) Schematic of replicate structure. (B) Subamplicon sequencing strategy workflow [27]. (C) Number of reads per barcode. (D) Number of barcodes per subamplicon. (E) Correlation plots of the absolute site differential selection [28] between biological and technical replicates for HSF1 inhibition at 39°C compared to a basal environment at 37°C. Best-fit line is plotted, with correlation coefficient and the *p*-value for significance of the slope deviating from zero shown on each plot. Complete sequencing data analysis is provided in S1 File. HSF1, heat shock factor 1.
(TIF)

S4 Fig Representative full sequence logo plot for nucleoprotein: HSF1-inhibited environment at 39°C relative to a basal environment at 37°C. Wild-type influenza A/Aichi/2/1968 nucleoprotein sequence and residue numbers are shown below logo plot. The size of the amino acid letters corresponds to the magnitude of the mutational differential selection, which is on the same scale for S4–S7 Figs and S10 Fig. Amino acids above the black line are more fit in the HSF1-inhibited environment at 39°C compared to a basal environment at 37°C, amino acids below are less fit, and the black line represents the behavior of the wild-type amino acid in the selection condition. Differential selection values are provided in S3 and S4 Data. HSF1, heat shock factor 1.
(TIF)

S5 Fig Representative full sequence logo plot for nucleoprotein: HSF1-inhibited environment at 39°C relative to a basal environment at 39°C. Wild-type influenza A/Aichi/2/1968 nucleoprotein sequence and residue numbers are shown below the logo plot. The size of the amino acid letters corresponds to the magnitude of the mutational differential selection, which is on the same scale for S4–S7 Figs and S10 Fig. Amino acids above the black line are more fit in an HSF1-inhibited environment at 39°C compared to a basal environment at 39°C, amino acids below are less fit, and the black line represents behavior of the wild-type amino acid in the selection condition. Differential selection values are provided in S3 and S4 Data. HSF1, heat shock factor 1.
(TIF)

S6 Fig Representative full sequence logo plot for nucleoprotein: 39°C relative to 37°C in basal environment. Wild-type influenza A/Aichi/2/1968 nucleoprotein sequence and residue numbers are shown below the logo plot. The size of the amino acid letters corresponds to the magnitude of the mutational differential selection, which is on the same scale for S4–S7 Figs and S10 Fig. Amino acids above the black line are more fit at 39°C compared to 37°C, amino acids below are less fit, and the black line represents the behavior of the wild-type amino acid in the selection condition. Differential selection values are provided in S3 and S4 Data.
(TIF)

S7 Fig Representative full sequence logo plot for nucleoprotein: HSF1-inhibited environment at 37°C relative to a basal environment at 37°C. Wild-type influenza A/Aichi/2/1968 nucleoprotein sequence and residue numbers are shown below the logo plot. The size of the amino acid letters corresponds to the magnitude of the mutational differential selection, which is on the same scale for S4–S7 Figs and S10 Fig. Amino acids above the black line are more fit in an HSF1-inhibited environment at 37°C compared to a basal environment at 37°C, amino acids below are less fit, and the black line represents the behavior of the wild-type amino acid in the selection condition. Differential selection values are provided in S3 and S4 Data. HSF1, heat shock factor 1.
(TIF)

S8 Fig Pairwise competitions recapitulate deep mutational scanning batch competition. (A) Error bars indicate the standard error from biological replicates (*N* = 3). The significance of the deviation from zero (no selection) was evaluated by a one-sample *t* test followed by Benjamini-Hochberg adjustment for multiple comparison; * and ** indicate FDR < 0.05 and < 0.01, respectively. Replicate differential selection values are provided in S5 Data. FDR, false discovery rate.
(TIF)

S9 Fig Purification and thermal denaturation of recombinant nucleoprotein variants. (A) Purified nucleoprotein (56 kDa) by Ni-NTA column chromatography and size exclusion chromatography. (B) Circular dichroism wavelength scans at 20°C for nucleoprotein variants. All variants exhibited similar circular dichroism spectra characteristic of an α-helical protein. Scans for each variant were performed in triplicate. (C) Thermal denaturation curves for nucleoprotein. Apparent (irreversible) melting temperatures (*T*_agg_) were obtained from sigmoidal fits over 20–60°C. Thermal denaturation of each variant was performed in triplicate (see S7 Data).
(TIF)

S10 Fig Representative full sequence logo plot for nucleoprotein: Hsp90-inhibited environment at 39°C relative to basal environment at 39°C. Wild-type influenza A/Aichi/2/1968 nucleoprotein sequence and residue numbers are shown below the logo plot. Deep mutational scanning was also performed in an Hsp90-inhibited environment to determine whether Hsp90 caused the fitness effects observed in the HSF1i environment; see Discussion. The size of the amino acid letters corresponds to the magnitude of the mutational differential selection, which is on the same scale for S4–S7 Figs and S10 Fig. Amino acids above the black line are more fit upon Hsp90 inhibition at 39°C compared to a basal environment at 39°C, amino acids below are less fit, and the black line represents the behavior of the wild-type amino acid in the selection condition. Differential selection values are provided in S3 and S4 Data. HSF1i, condition in which heat shock factor 1 was inhibited; Hsp90, heat shock protein 90.
(TIF)

S1 Data RNA-seq differential expression analysis. RNA-seq, RNA sequencing.
(XLSX)

S2 Data Characterization of HSF1 inhibition. HSF1, heat shock factor 1.
(XLSX)

S3 Data Site differential selection values for each site in nucleoprotein. (XLSX)

S4 Data Mutation differential selection values for each mutation in nucleoprotein. (XLSX)

S5 Data Pairwise competition differential selection values. (XLSX)

S6 Data Molecular dynamics α-helicity percentages. (XLSX)

S7 Data Aggregation temperatures of recombinant nucleoprotein variants. (XLSX)

S8 Data Pairwise competition in the presence of MxA: Ser283 frequencies. MxA, Myxovirus resistance protein A.
(XLSX)

S9 Data Post hoc comparisons of Ser283 frequency changes in pairwise competitions in the presence of MxA. MxA, Myxovirus resistance protein A.
(XLSX)

S1 Table Primer sequences for qPCR and sequencing. qPCR, quantitative PCR.
(XLSX)

S1 Video Molecular dynamics simulation of the Pro283 nucleoprotein variant. For clarity, structural evolution of only the region from amino acids 279–288 in a single representative simulation trajectory is shown. Purple = α-helical conformation; blue = 3,10-helical conformation.
(AVI)

S2 Video Molecular dynamics simulation of the Ser283 nucleoprotein variant. For clarity, structural evolution of only the region from amino acids 279–288 in a single representative simulation trajectory is shown. Purple = α-helical conformation; blue = 3,10-helical conformation.
(AVI)

S1 File Computer code and deep mutational scanning analysis data. This zip file contains the computer code and required input files for the deep mutational scanning data analysis reported in this manuscript.
(ZIP)

## Abbreviations

Ala: alanine
ANOVA: analysis of variance
FBS: fetal bovine serum
FDR: false discovery rate
Gly: glycine
HSF1: heat shock factor 1
HSF1i: condition in which HSF1 was inhibited
Hsp40: heat shock protein 40
Hsp70: heat shock protein 70
Hsp90: heat shock protein 90
IRES: internal ribosome entry site
Leu: leucine
MD: molecular dynamics
MDCK: Madin Darby canine kidney
MxA: Myxovirus resistance protein A
NP: nucleoprotein
ns: not significant
PME: particle mesh Ewald
Pro: proline
Ser: serine
TCID_50_: tissue culture infectious dose
