# Supplementary figures and images for: Destabilized adaptive influenza variants critical for innate immune system escape are potentiated by host chaperones
Source: PLoS Biol. 2018 Sep 17;16(9):e3000008. doi: 10.1371/journal.pbio.3000008 (PMC6160216; doi:10.1371/journal.pbio.3000008)

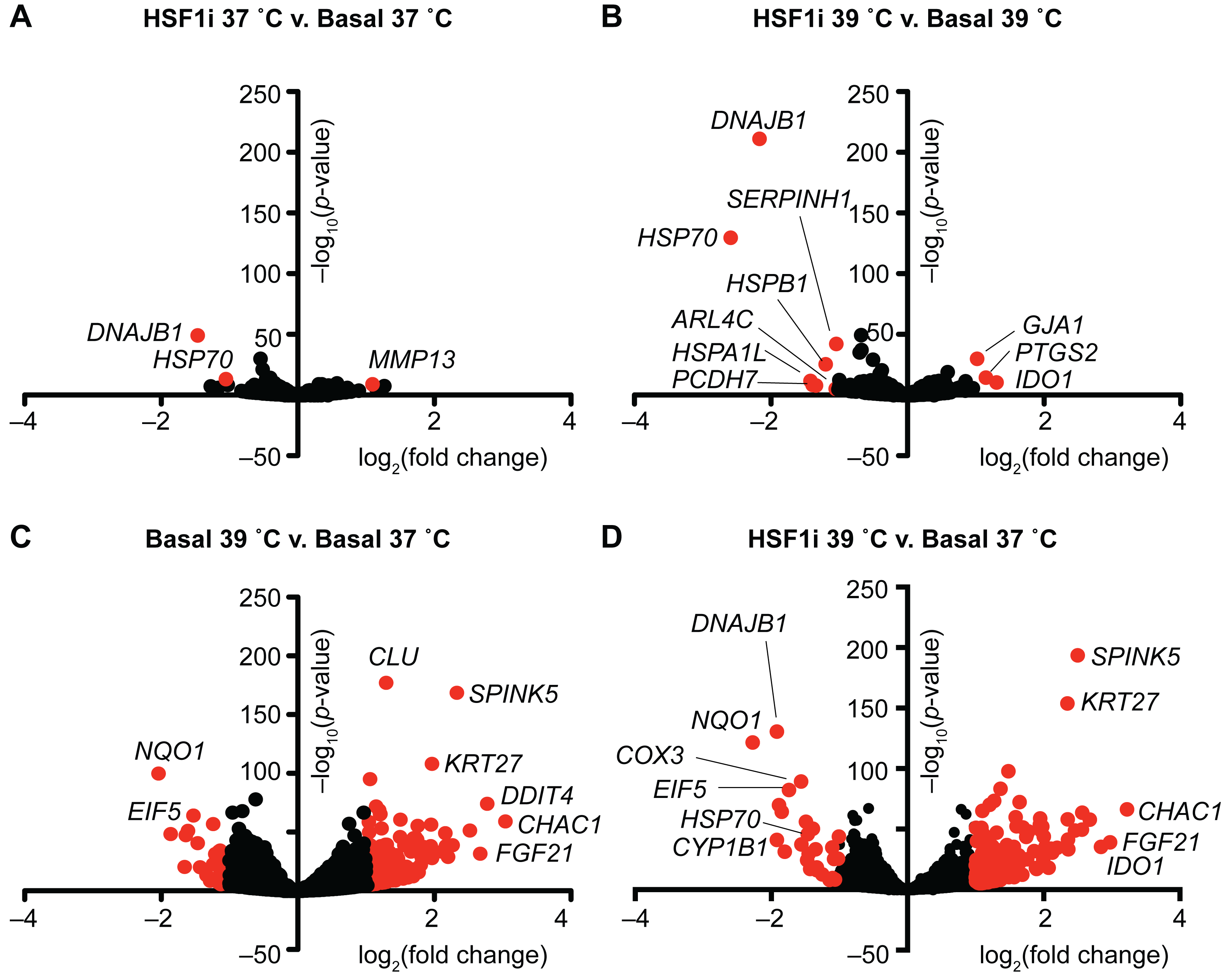

Supplement: S1 Fig — (A) Volcano plot of RNA-seq data for HSF1 inhibition at 37 °C. (B) Volcano plot of RNA-seq data for HSF1 inhibition at 39 °C. (C) Volcano plot of RNA-seq data for 39 °C relative to 37 °C in basal environment. (D) Volcano plot of RNA-seq data for HSF1-inhibited environment at 39 °C relative to a basal environment at 37 °C. For A–D, transcripts with >2-fold change and p-values < 10−5 are shown in red, with outliers labeled. The complete RNA-seq differential expression analysis is provided in S1 Data. HSF1, heat shock factor 1; RNA-seq, RNA sequencing. (TIF) [file pbio.3000008.s001.tif]

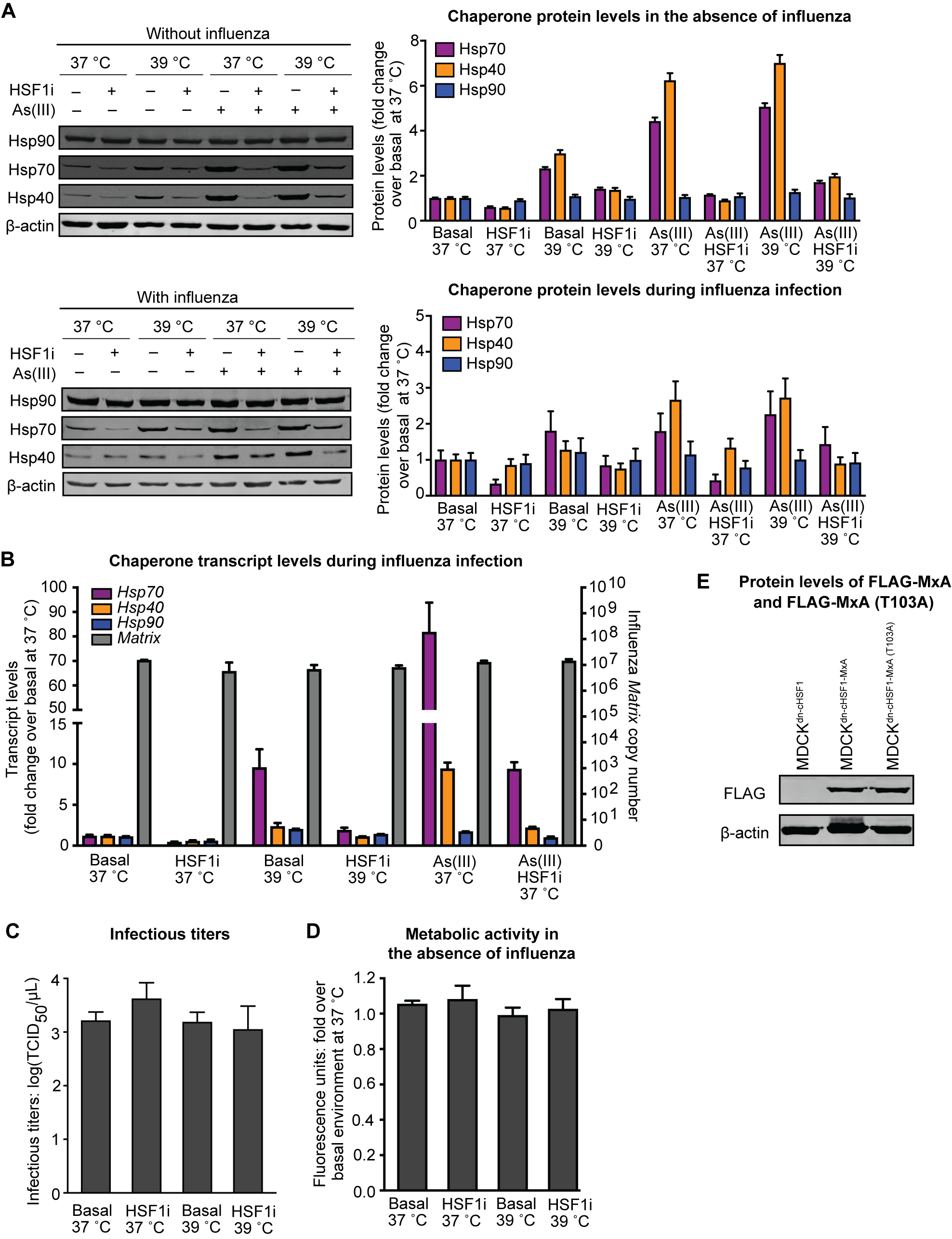

Supplement: S2 Fig — (A) Protein levels of heat shock protein chaperones in basal and chaperone-depleted host cells at 37°C and 39°C, in the absence or presence of influenza. Representative western blots on the left; quantitation of biological triplicates on the right. Arsenite (As(III)) is a chemical stressor that induces the heat shock response. (B) Transcript expression in selection conditions during influenza infection. (C) Infectious titers determined by TCID50 for wild-type A/Aichi/2/1968 (H3N2) in selection conditions. (D) Metabolic activity of MDCKdn-cHSF1 cells in each selection condition, as characterized by resazurin assays at 48 hours post treatment. (E) Protein levels of FLAG-MxA and FLAG-MxA(T103A) in MDCKdn-cHSF1-MxA and MDCKdn-cHSF1-MxA(T103A) cells, respectively. MDCKdn-cHSF1 is shown as a negative control. Representative blot is shown (N = 2). For A–D, replicate data are provided in S2 Data. HSF1, heat shock factor 1; MDCK, Madin Darby canine kidney; TCID50, tissue culture infectious dose. (TIF) [file pbio.3000008.s002.tif]

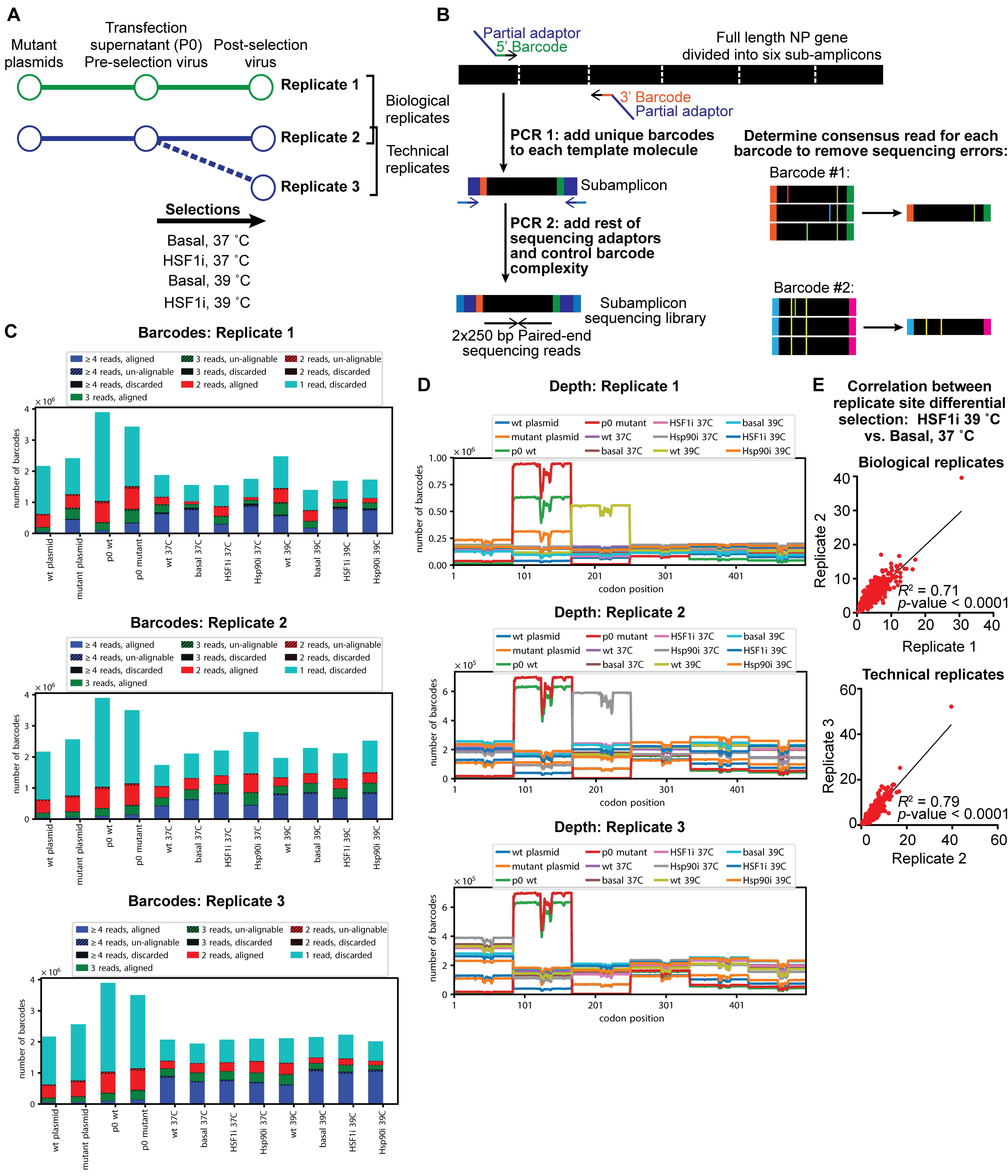

Supplement: S3 Fig — (A) Schematic of replicate structure. (B) Subamplicon sequencing strategy workflow [27]. (C) Number of reads per barcode. (D) Number of barcodes per subamplicon. (E) Correlation plots of the absolute site differential selection [28] between biological and technical replicates for HSF1 inhibition at 39°C compared to a basal environment at 37°C. Best-fit line is plotted, with correlation coefficient and the p-value for significance of the slope deviating from zero shown on each plot. Complete sequencing data analysis is provided in S1 File. HSF1, heat shock factor 1. (TIF) [file pbio.3000008.s003.tif]

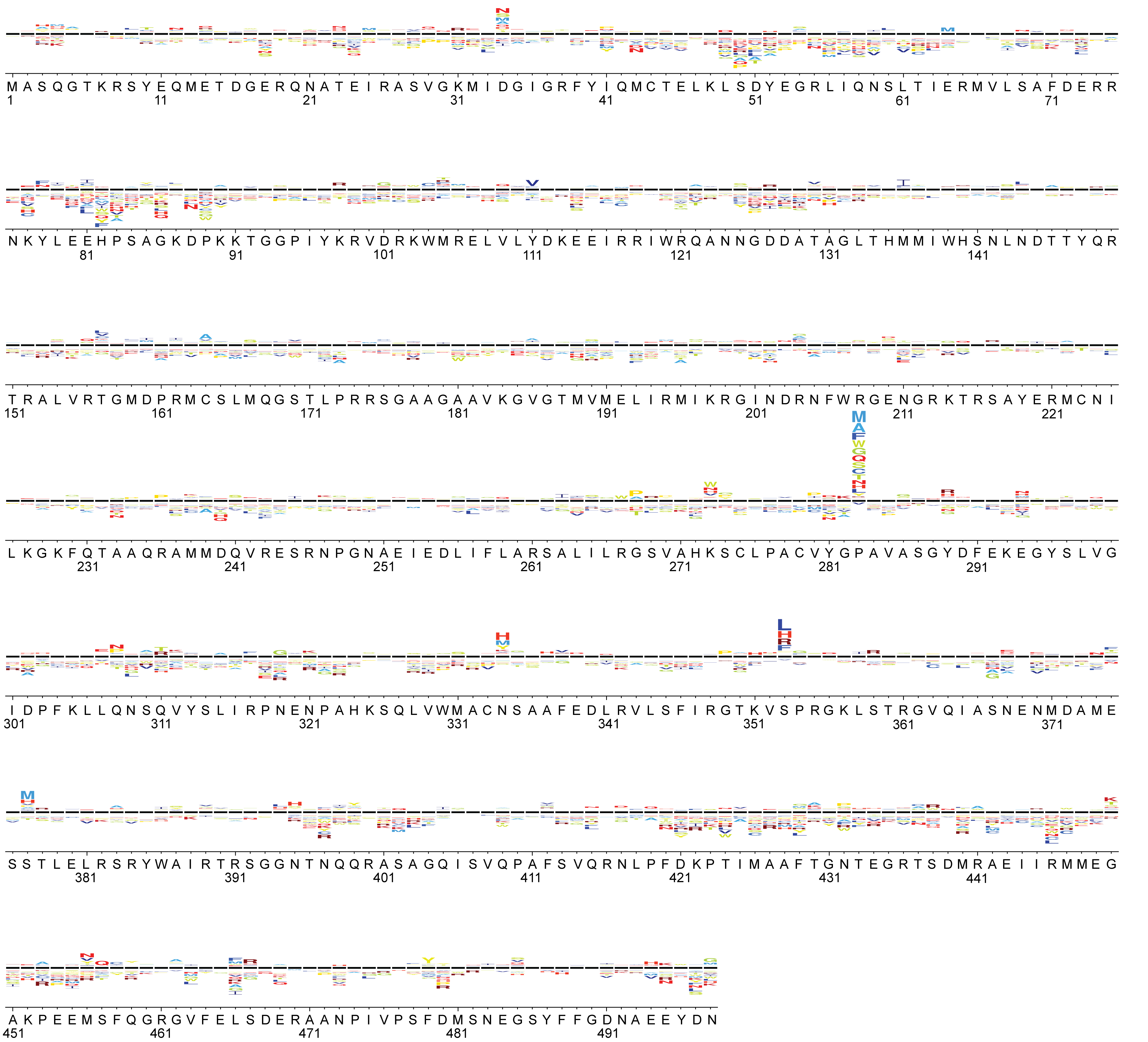

Supplement: S4 Fig — Wild-type influenza A/Aichi/2/1968 nucleoprotein sequence and residue numbers are shown below logo plot. The size of the amino acid letters corresponds to the magnitude of the mutational differential selection, which is on the same scale for S4–S7 Figs and S10 Fig. Amino acids above the black line are more fit in the HSF1-inhibited environment at 39°C compared to a basal environment at 37°C, amino acids below are less fit, and the black line represents the behavior of the wild-type amino acid in the selection condition. Differential selection values are provided in S3 and S4 Data. HSF1, heat shock factor 1. (TIF) [file pbio.3000008.s004.tif]

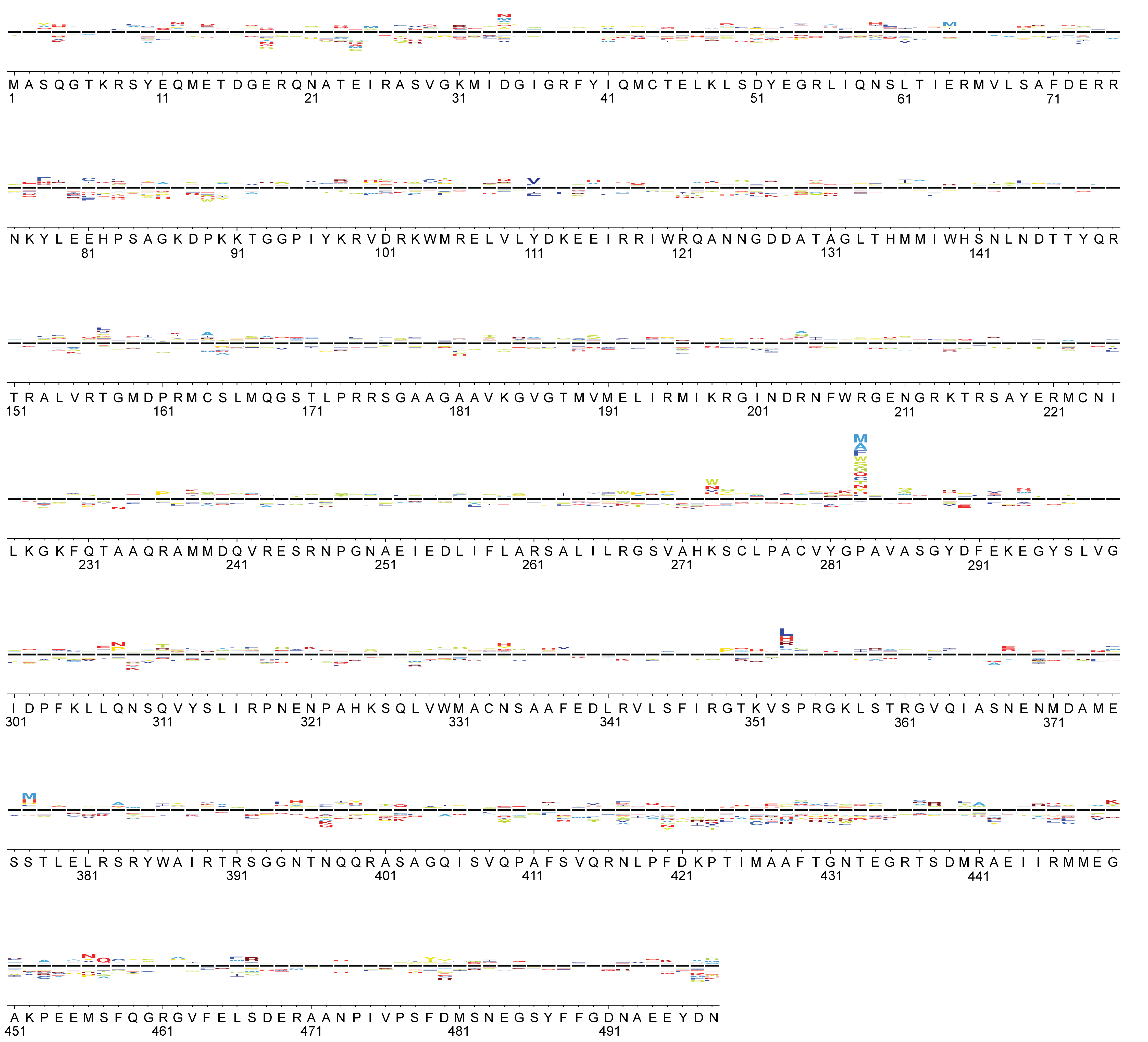

Supplement: S5 Fig — Wild-type influenza A/Aichi/2/1968 nucleoprotein sequence and residue numbers are shown below the logo plot. The size of the amino acid letters corresponds to the magnitude of the mutational differential selection, which is on the same scale for S4–S7 Figs and S10 Fig. Amino acids above the black line are more fit in an HSF1-inhibited environment at 39°C compared to a basal environment at 39°C, amino acids below are less fit, and the black line represents behavior of the wild-type amino acid in the selection condition. Differential selection values are provided in S3 and S4 Data. HSF1, heat shock factor 1. (TIF) [file pbio.3000008.s005.tif]

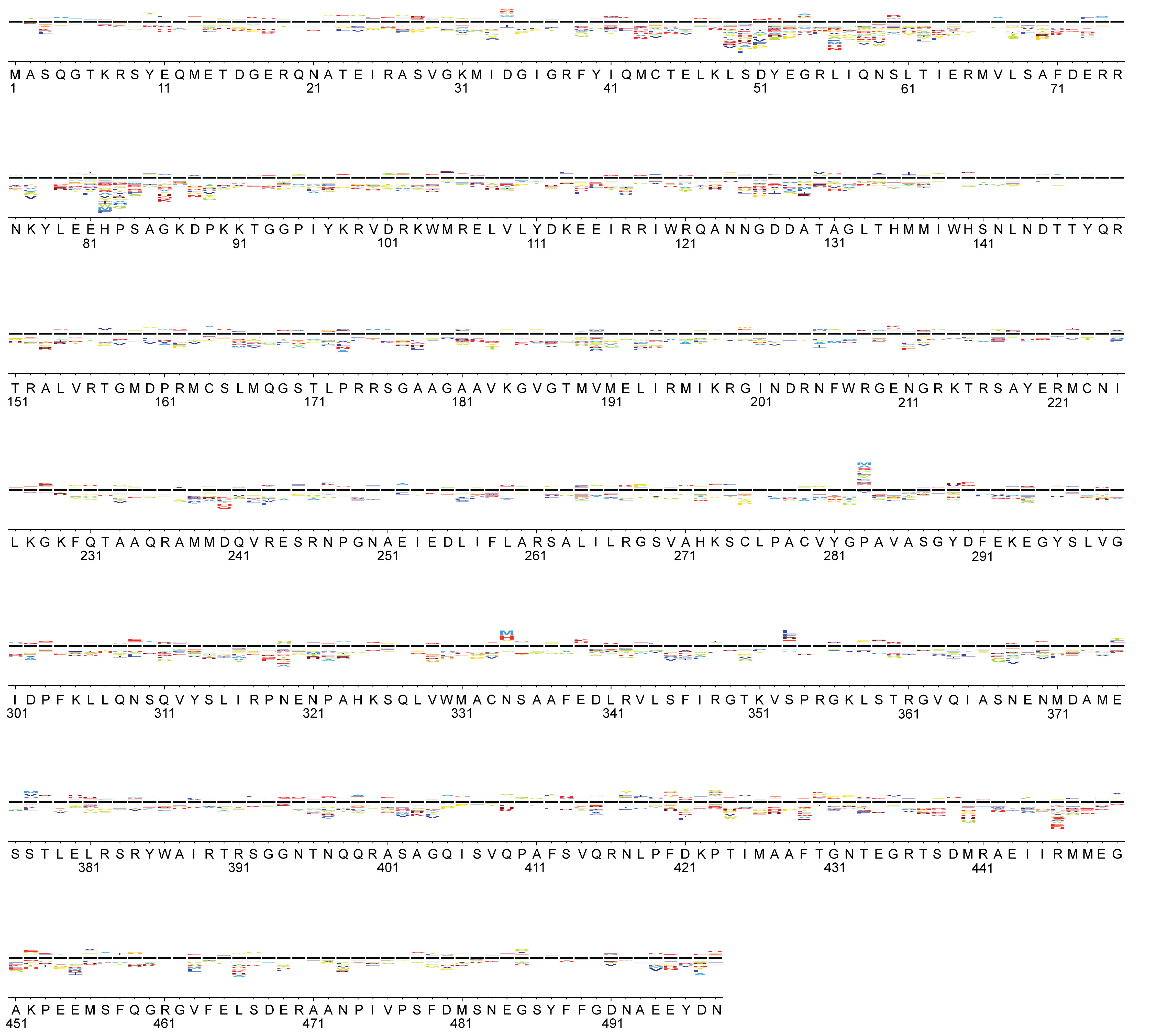

Supplement: S6 Fig — Wild-type influenza A/Aichi/2/1968 nucleoprotein sequence and residue numbers are shown below the logo plot. The size of the amino acid letters corresponds to the magnitude of the mutational differential selection, which is on the same scale for S4–S7 Figs and S10 Fig. Amino acids above the black line are more fit at 39°C compared to 37°C, amino acids below are less fit, and the black line represents the behavior of the wild-type amino acid in the selection condition. Differential selection values are provided in S3 and S4 Data. (TIF) [file pbio.3000008.s006.tif]

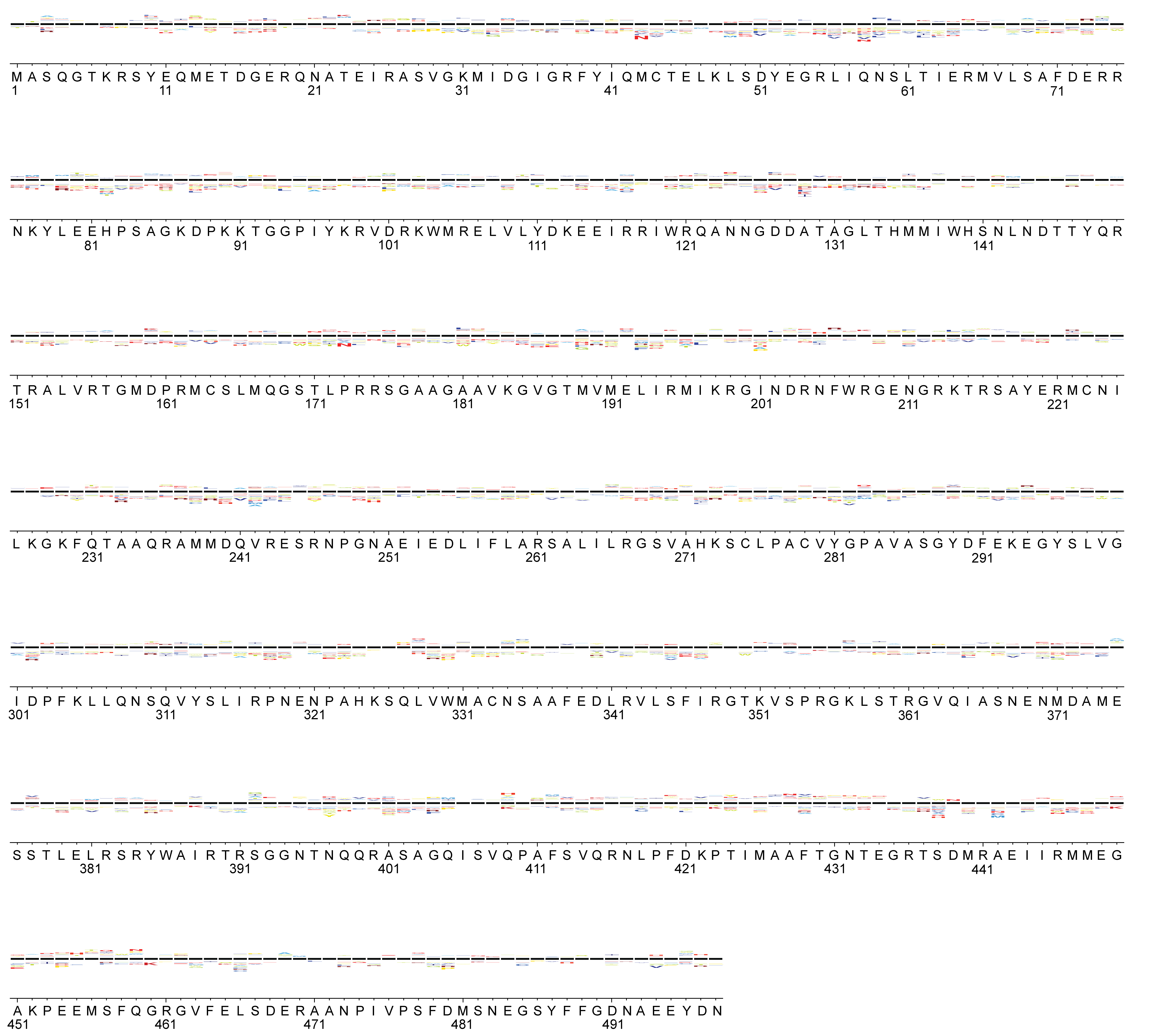

Supplement: S7 Fig — Wild-type influenza A/Aichi/2/1968 nucleoprotein sequence and residue numbers are shown below the logo plot. The size of the amino acid letters corresponds to the magnitude of the mutational differential selection, which is on the same scale for S4–S7 Figs and S10 Fig. Amino acids above the black line are more fit in an HSF1-inhibited environment at 37°C compared to a basal environment at 37°C, amino acids below are less fit, and the black line represents the behavior of the wild-type amino acid in the selection condition. Differential selection values are provided in S3 and S4 Data. HSF1, heat shock factor 1. (TIF) [file pbio.3000008.s007.tif]

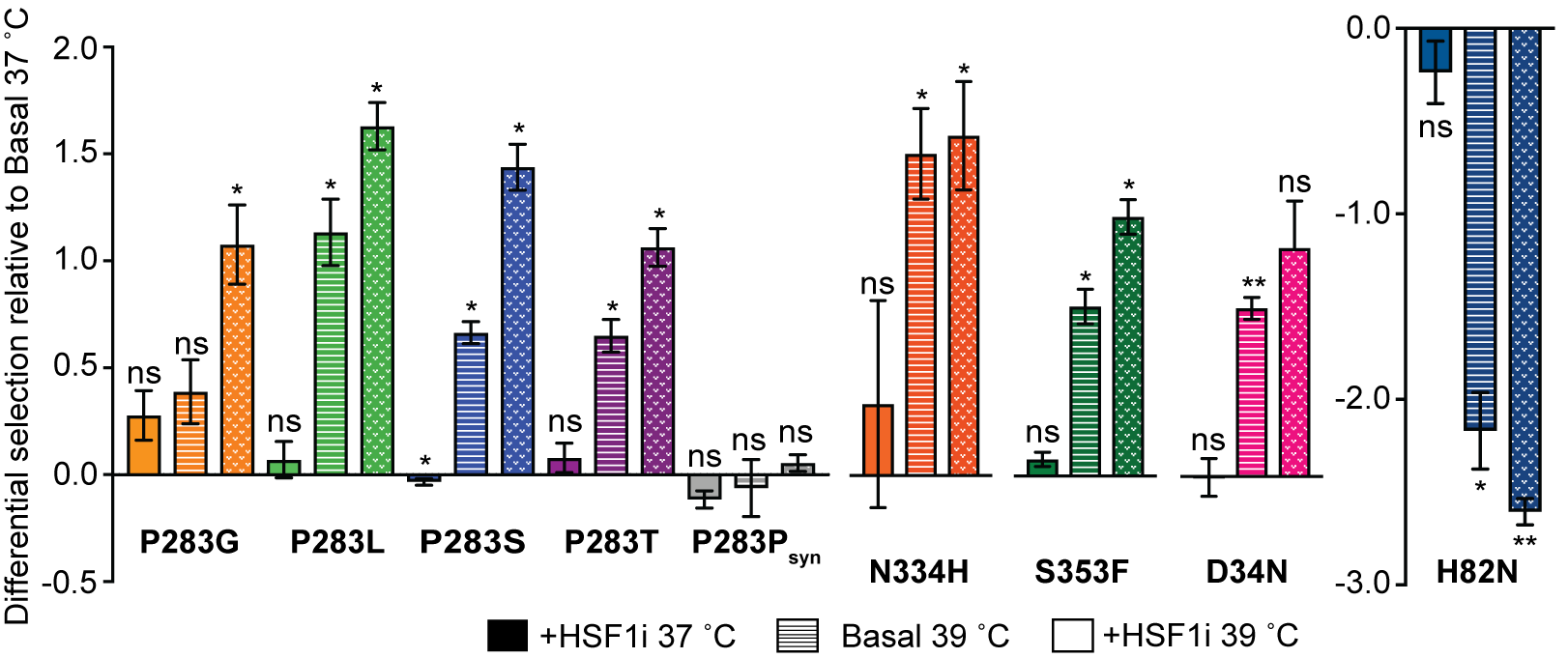

Supplement: S8 Fig — (A) Error bars indicate the standard error from biological replicates (N = 3). The significance of the deviation from zero (no selection) was evaluated by a one-sample t test followed by Benjamini-Hochberg adjustment for multiple comparison; * and ** indicate FDR < 0.05 and < 0.01, respectively. Replicate differential selection values are provided in S5 Data. FDR, false discovery rate. (TIF) [file pbio.3000008.s008.tif]

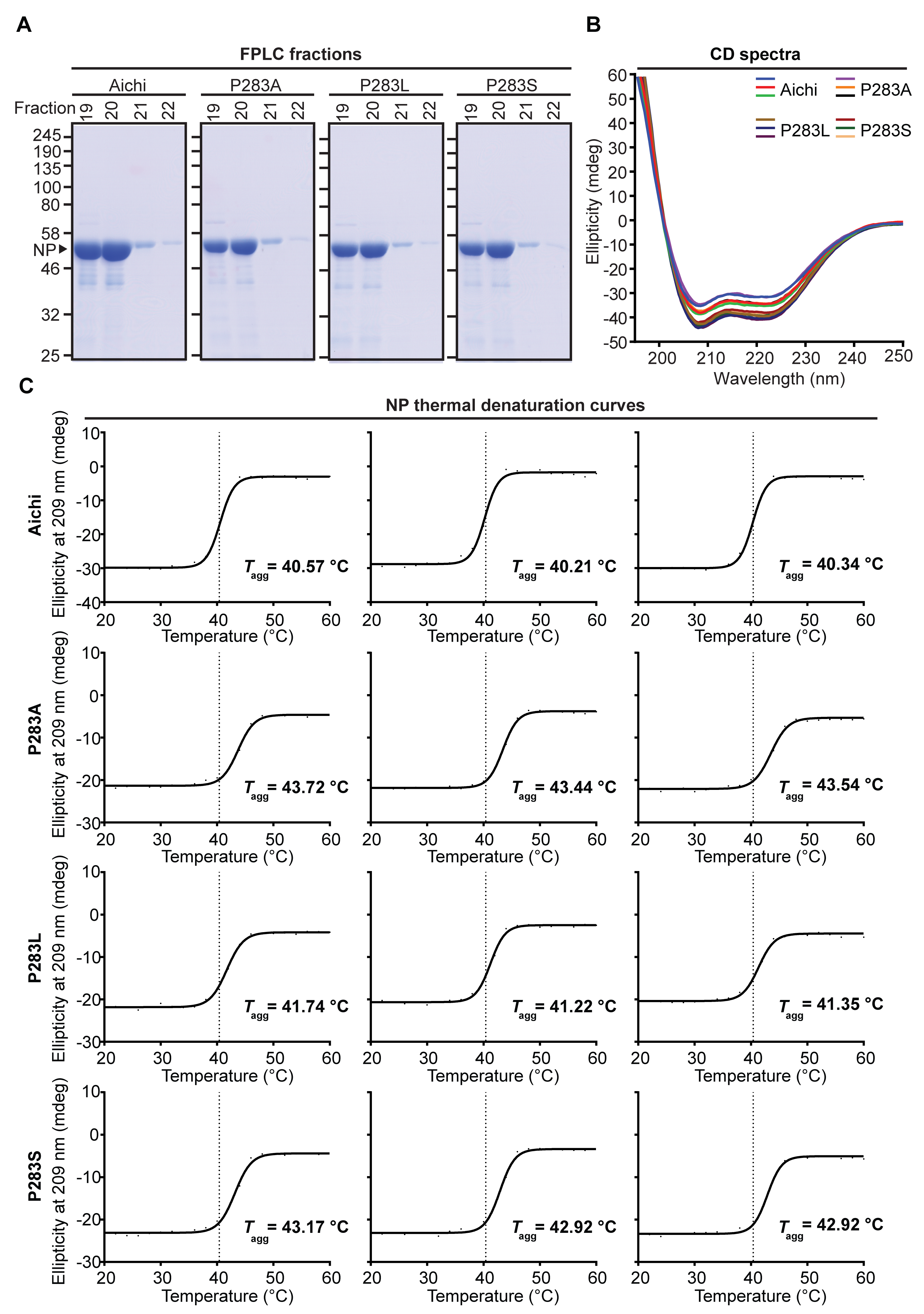

Supplement: S9 Fig — (A) Purified nucleoprotein (56 kDa) by Ni-NTA column chromatography and size exclusion chromatography. (B) Circular dichroism wavelength scans at 20°C for nucleoprotein variants. All variants exhibited similar circular dichroism spectra characteristic of an α-helical protein. Scans for each variant were performed in triplicate. (C) Thermal denaturation curves for nucleoprotein. Apparent (irreversible) melting temperatures (Tagg) were obtained from sigmoidal fits over 20–60°C. Thermal denaturation of each variant was performed in triplicate (see S7 Data). (TIF) [file pbio.3000008.s009.tif]

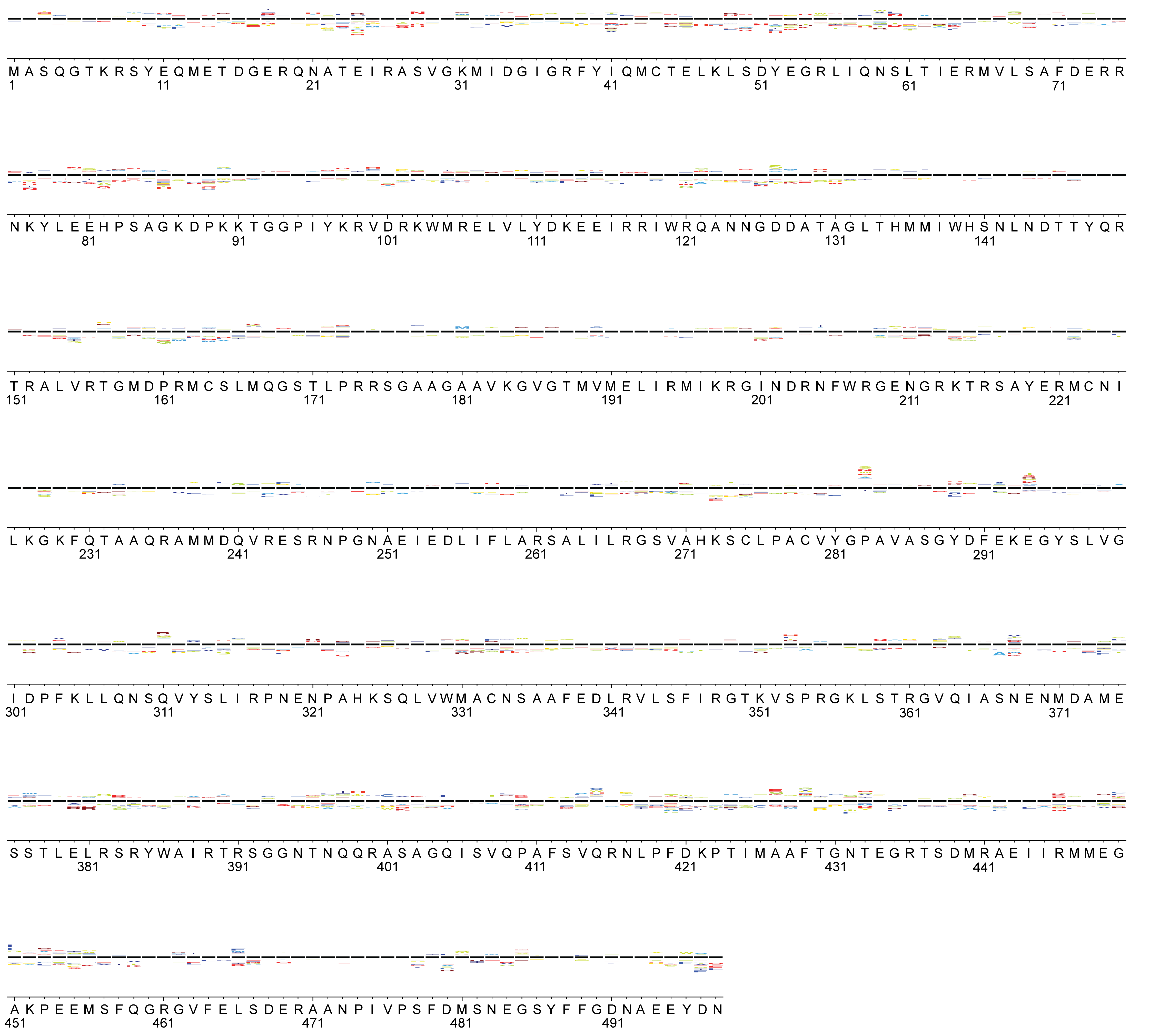

Supplement: S10 Fig — Wild-type influenza A/Aichi/2/1968 nucleoprotein sequence and residue numbers are shown below the logo plot. Deep mutational scanning was also performed in an Hsp90-inhibited environment to determine whether Hsp90 caused the fitness effects observed in the HSF1i environment; see Discussion. The size of the amino acid letters corresponds to the magnitude of the mutational differential selection, which is on the same scale for S4–S7 Figs and S10 Fig. Amino acids above the black line are more fit upon Hsp90 inhibition at 39°C compared to a basal environment at 39°C, amino acids below are less fit, and the black line represents the behavior of the wild-type amino acid in the selection condition. Differential selection values are provided in S3 and S4 Data. HSF1i, condition in which heat shock factor 1 was inhibited; Hsp90, heat shock protein 90. (TIF) [file pbio.3000008.s010.tif]

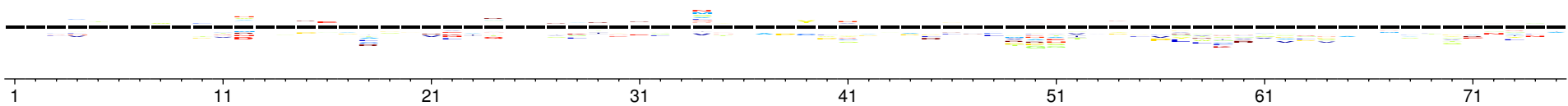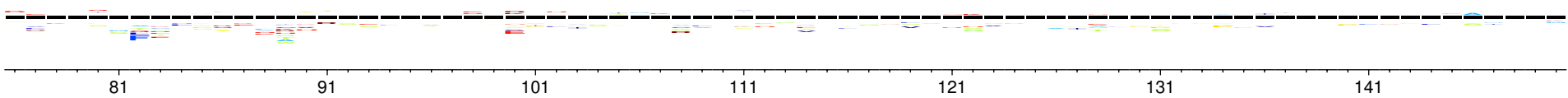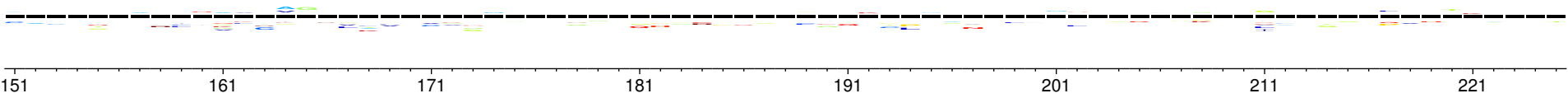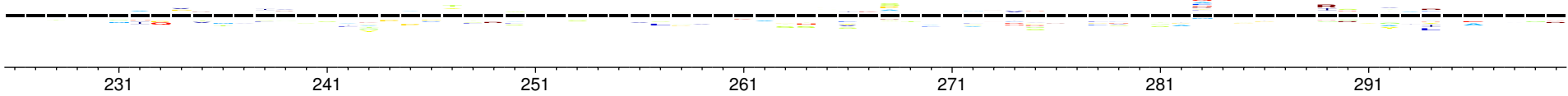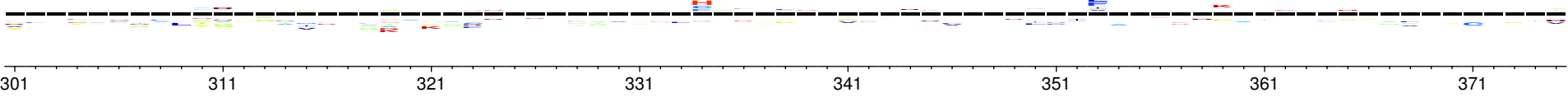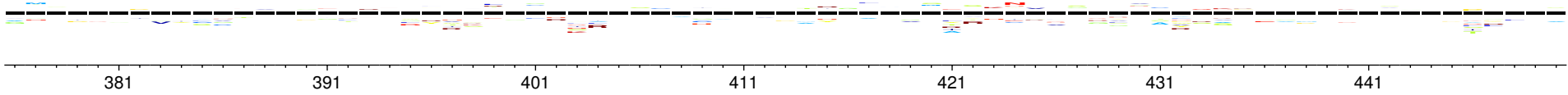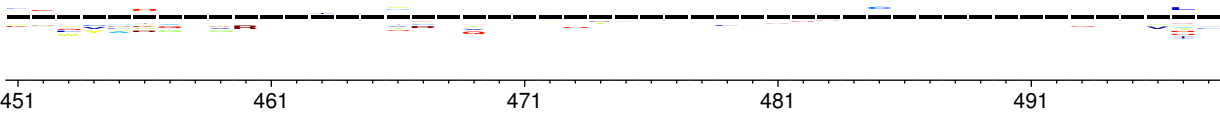

Supplement: S1 File — This zip file contains the computer code and required input files for the deep mutational scanning data analysis reported in this manuscript. (ZIP) [file pbio.3000008.s023.zip › 2018_NP_DMS-master/preferences/DMSO39vsDMSO37-rep1_diffsel_logoplot.pdf]

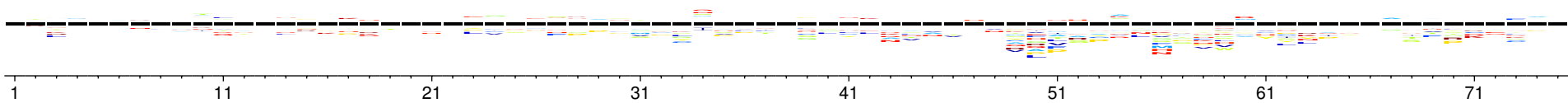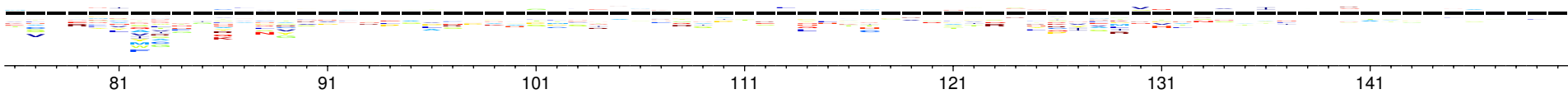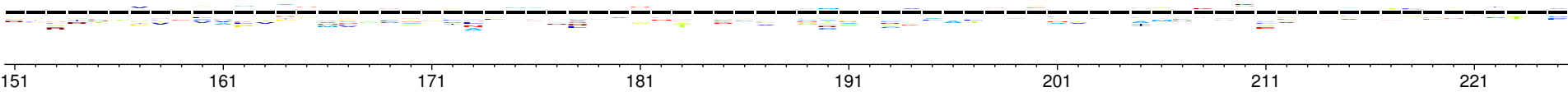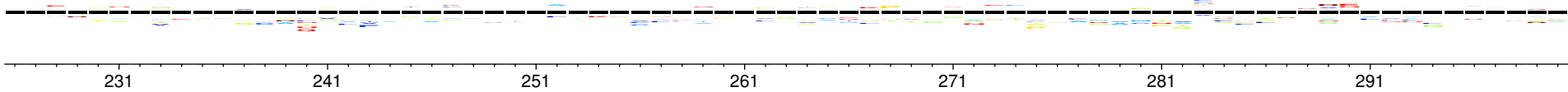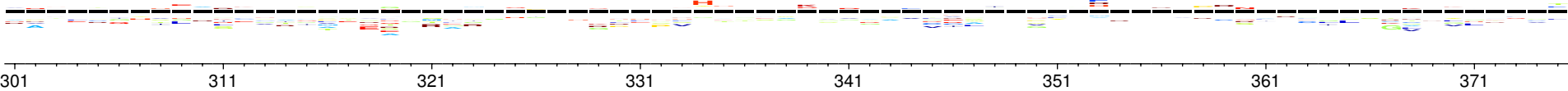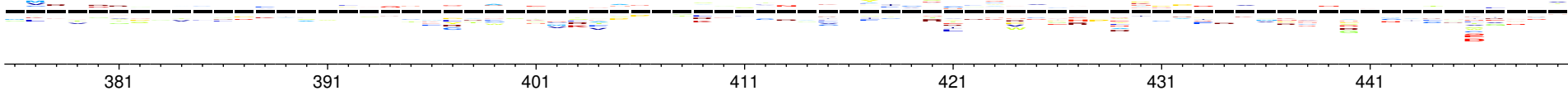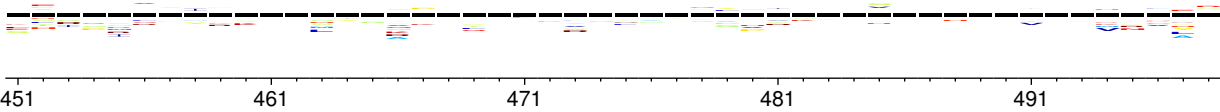

Supplement: S1 File — This zip file contains the computer code and required input files for the deep mutational scanning data analysis reported in this manuscript. (ZIP) [file pbio.3000008.s023.zip › 2018_NP_DMS-master/preferences/DMSO39vsDMSO37-rep2_diffsel_logoplot.pdf]

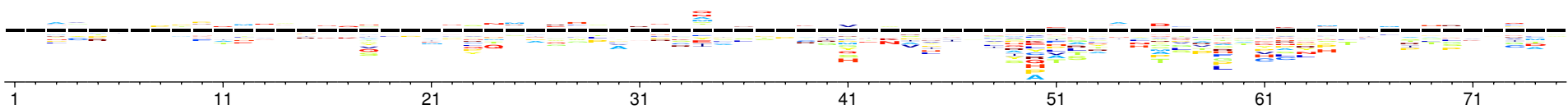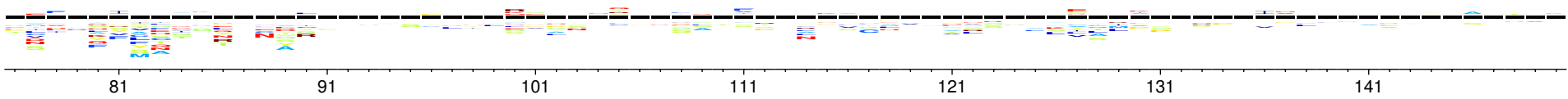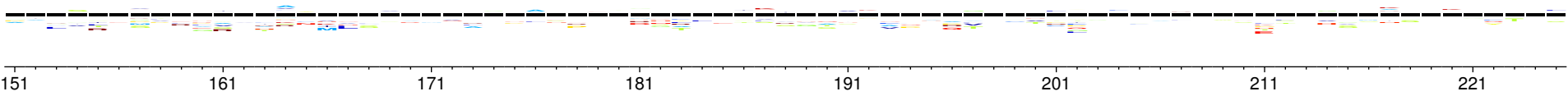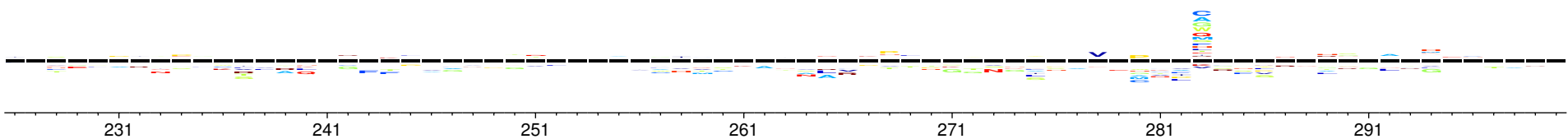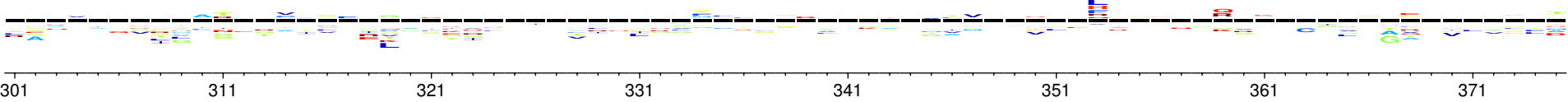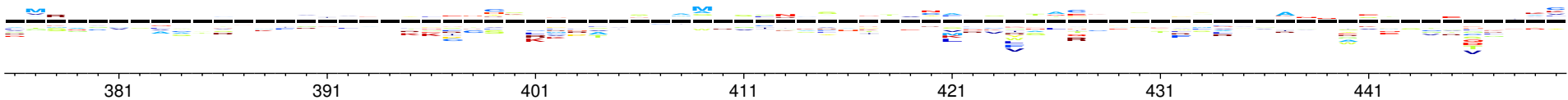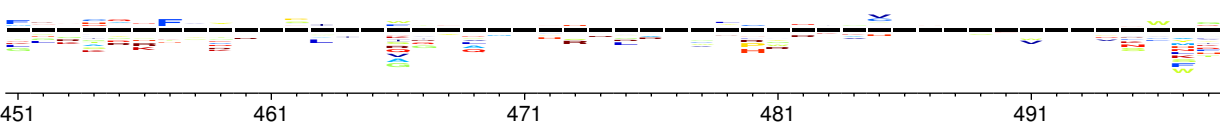

Supplement: S1 File — This zip file contains the computer code and required input files for the deep mutational scanning data analysis reported in this manuscript. (ZIP) [file pbio.3000008.s023.zip › 2018_NP_DMS-master/preferences/DMSO39vsDMSO37-rep3_diffsel_logoplot.pdf]

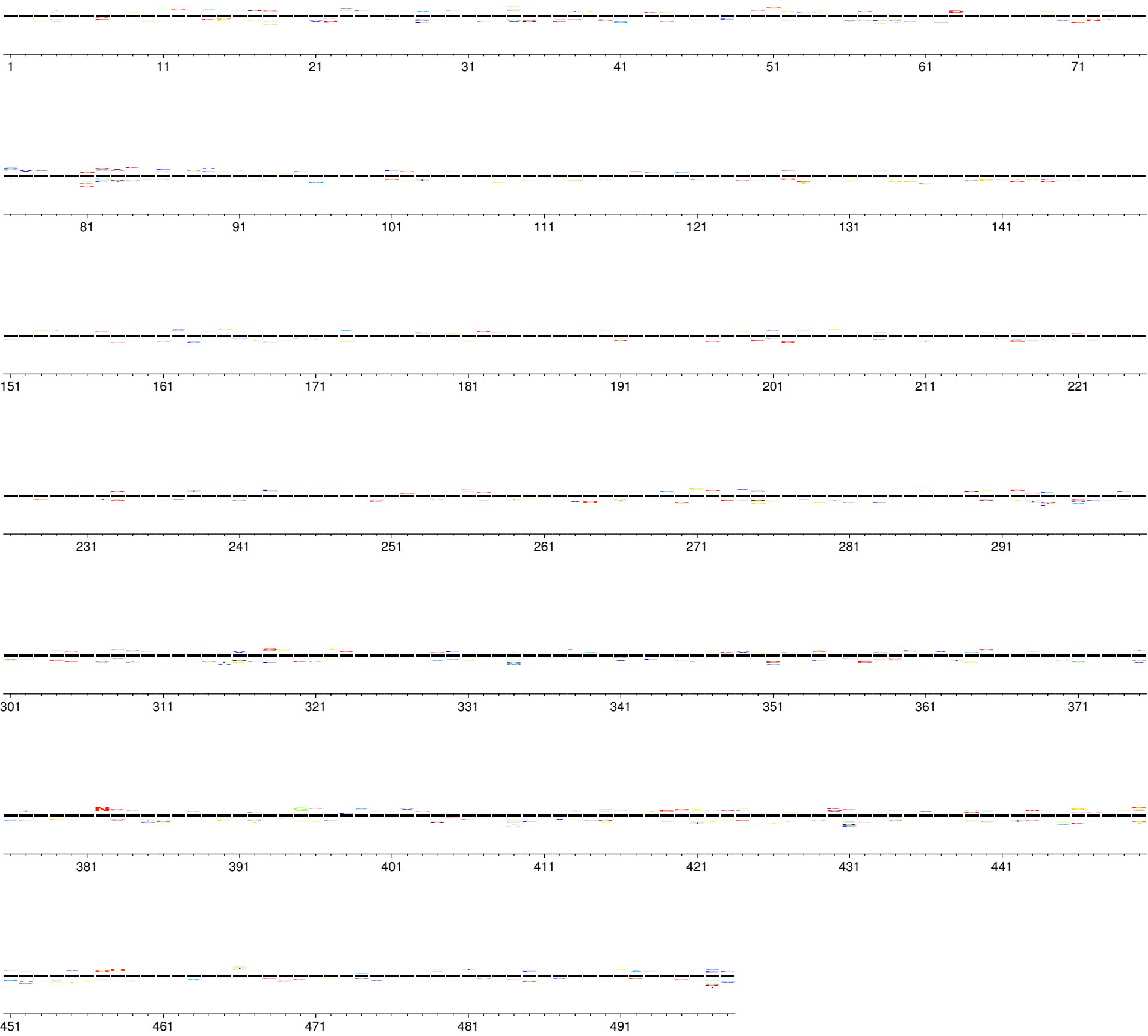

Supplement: S1 File — This zip file contains the computer code and required input files for the deep mutational scanning data analysis reported in this manuscript. (ZIP) [file pbio.3000008.s023.zip › 2018_NP_DMS-master/preferences/HSF1i37vsDMSO37-rep1_diffsel_logoplot.pdf]

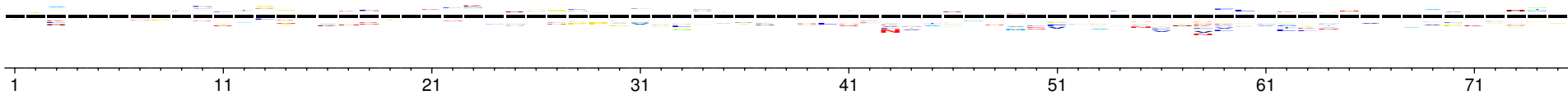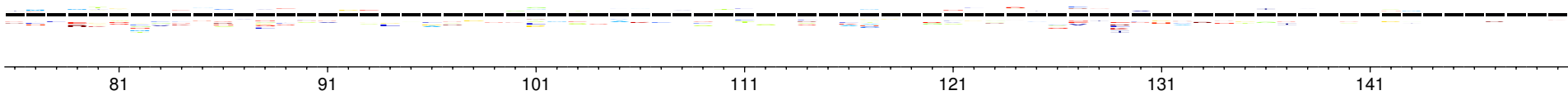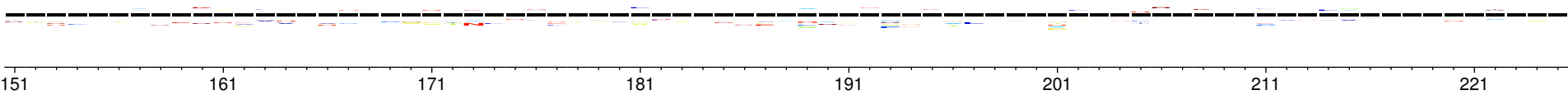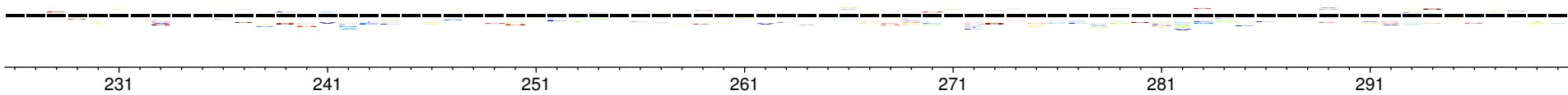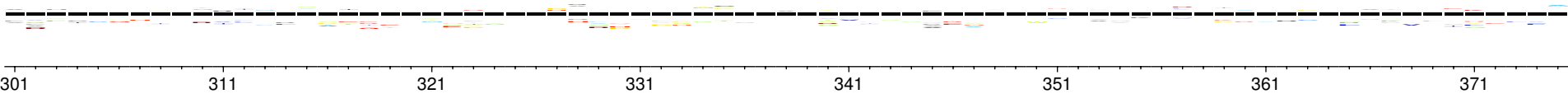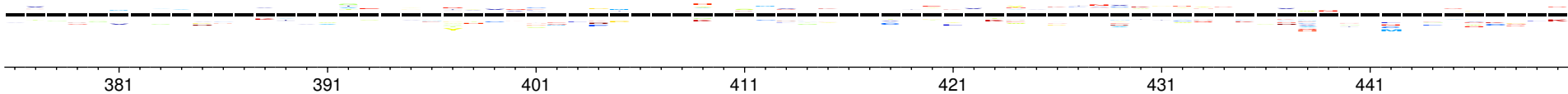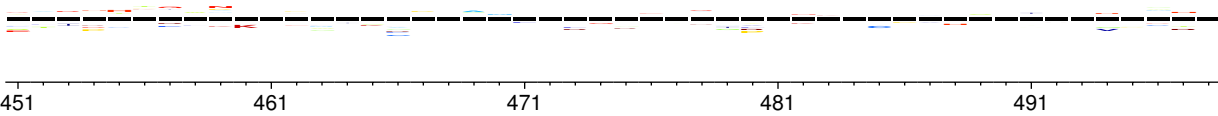

Supplement: S1 File — This zip file contains the computer code and required input files for the deep mutational scanning data analysis reported in this manuscript. (ZIP) [file pbio.3000008.s023.zip › 2018_NP_DMS-master/preferences/HSF1i37vsDMSO37-rep2_diffsel_logoplot.pdf]

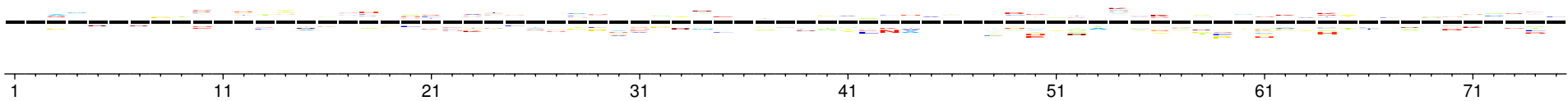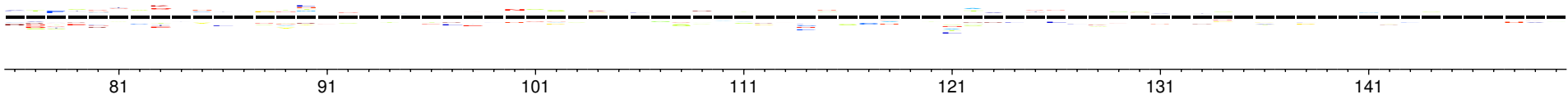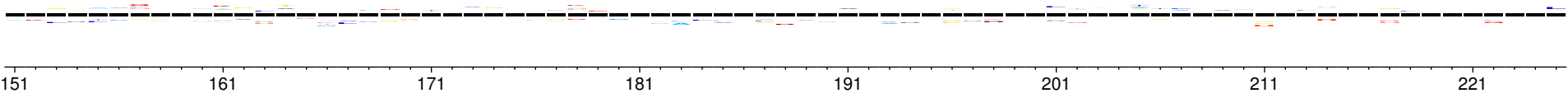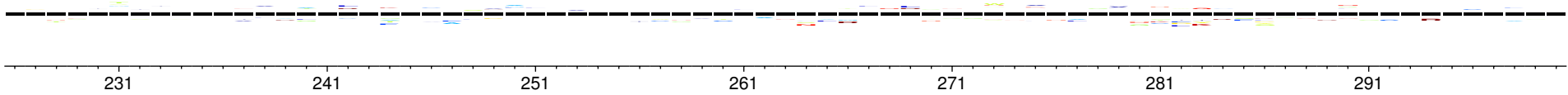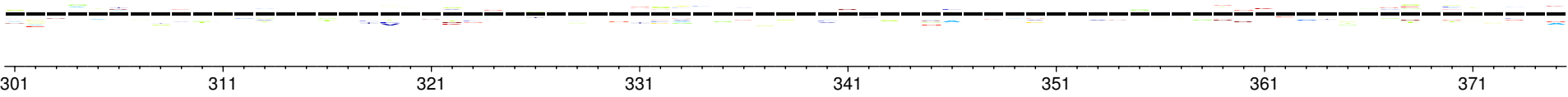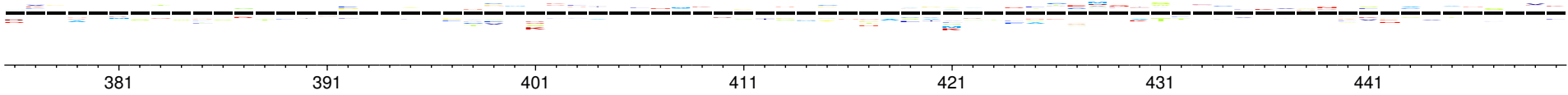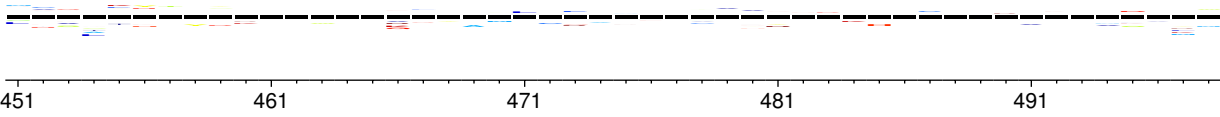

Supplement: S1 File — This zip file contains the computer code and required input files for the deep mutational scanning data analysis reported in this manuscript. (ZIP) [file pbio.3000008.s023.zip › 2018_NP_DMS-master/preferences/HSF1i37vsDMSO37-rep3_diffsel_logoplot.pdf]

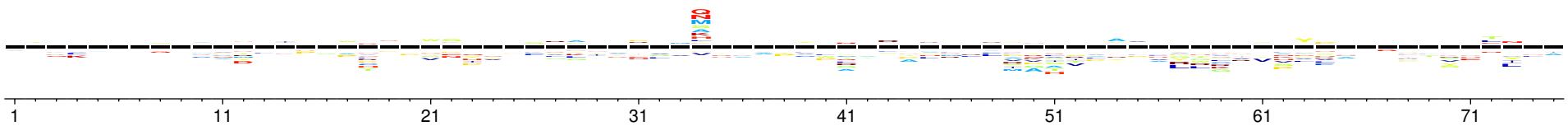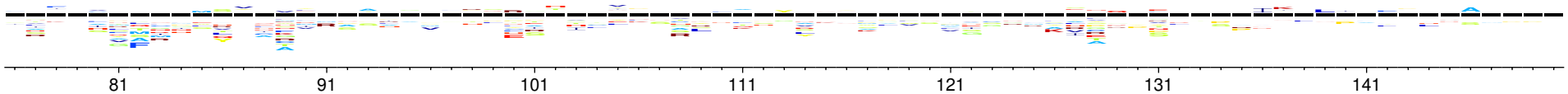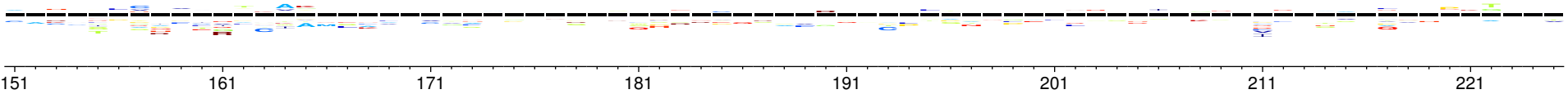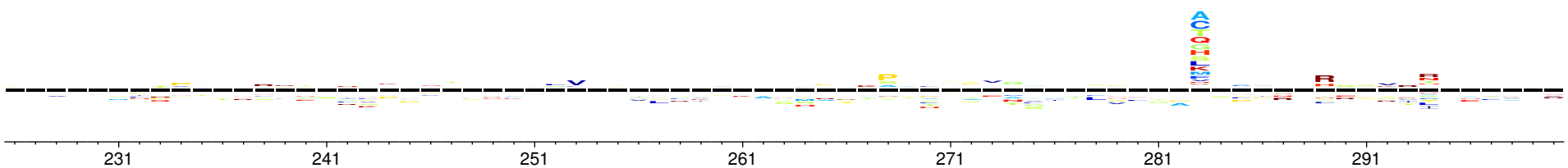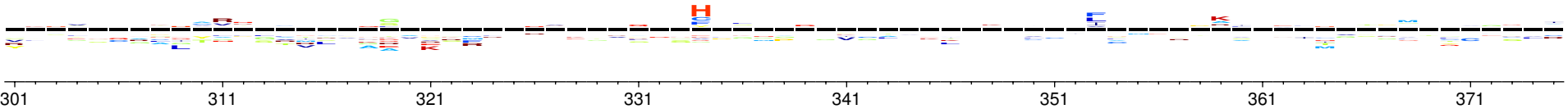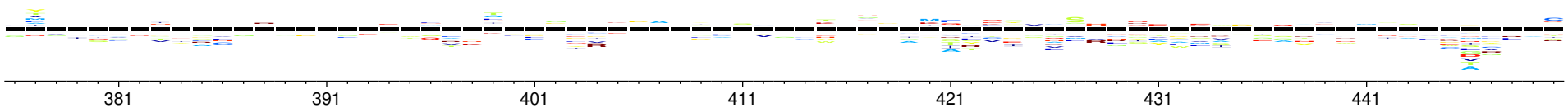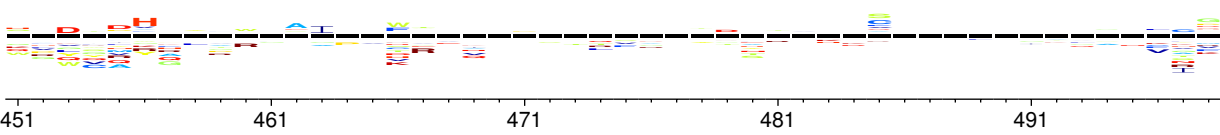

Supplement: S1 File — This zip file contains the computer code and required input files for the deep mutational scanning data analysis reported in this manuscript. (ZIP) [file pbio.3000008.s023.zip › 2018_NP_DMS-master/preferences/HSF1i39vsDMSO37-rep1_diffsel_logoplot.pdf]

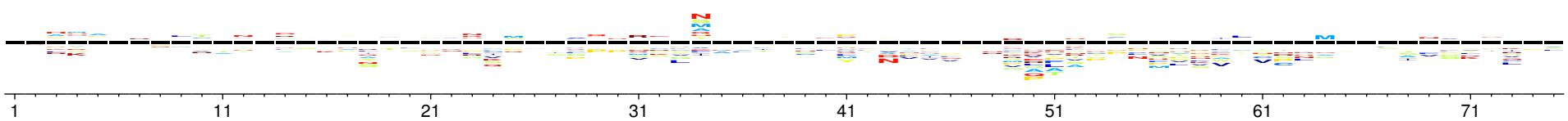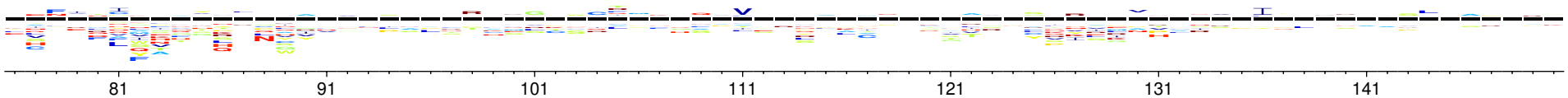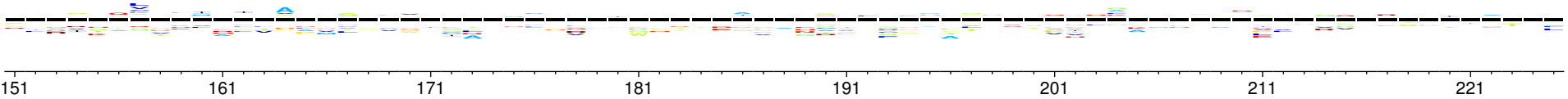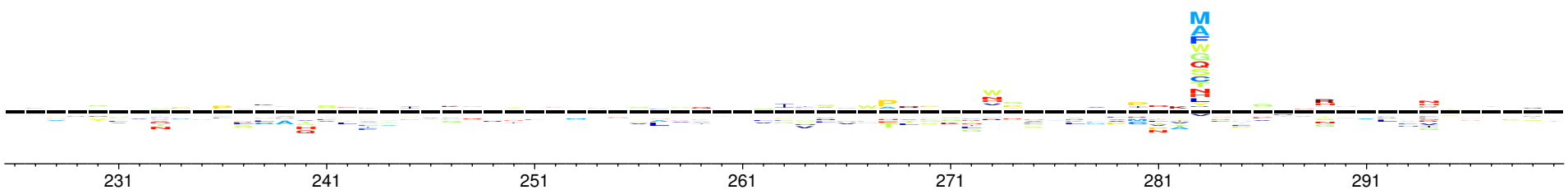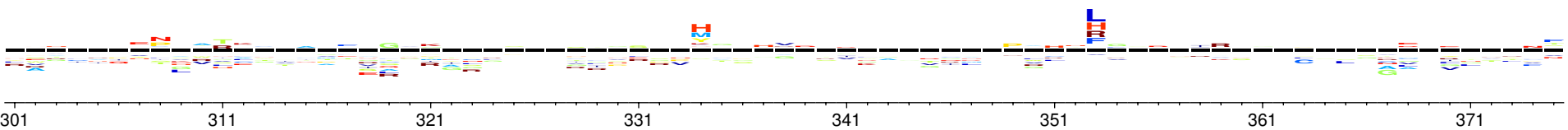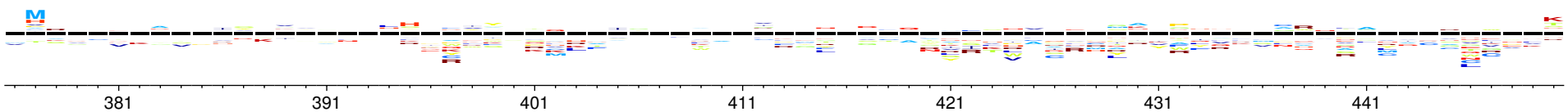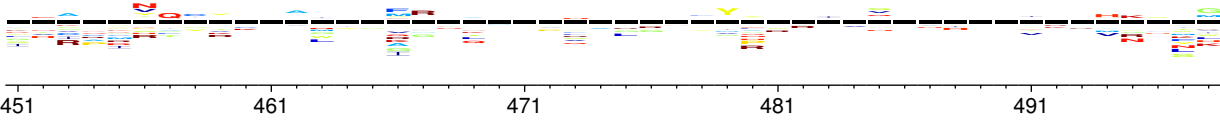

Supplement: S1 File — This zip file contains the computer code and required input files for the deep mutational scanning data analysis reported in this manuscript. (ZIP) [file pbio.3000008.s023.zip › 2018_NP_DMS-master/preferences/HSF1i39vsDMSO37-rep2_diffsel_logoplot.pdf]

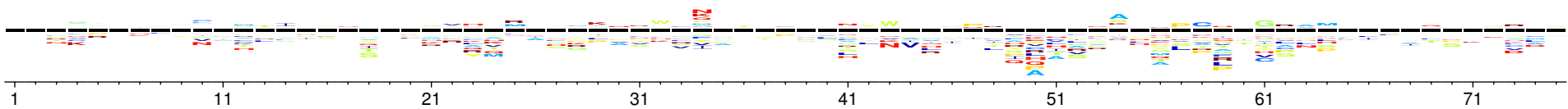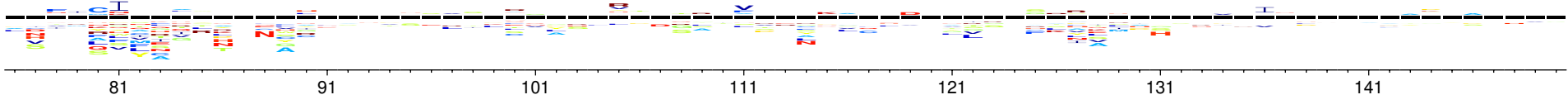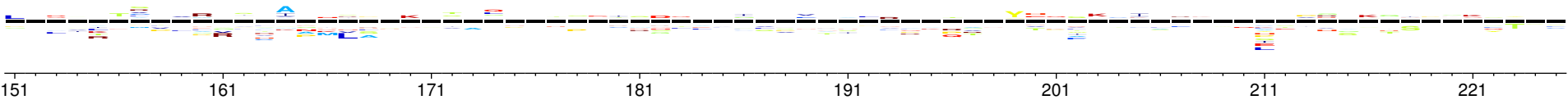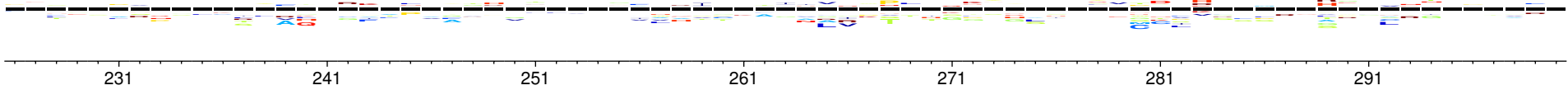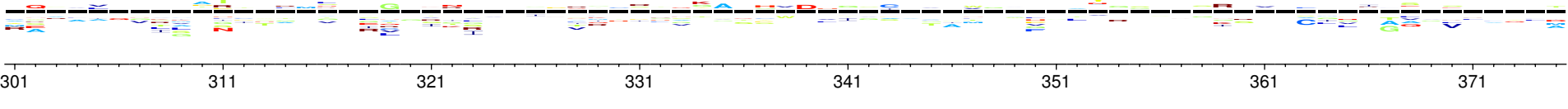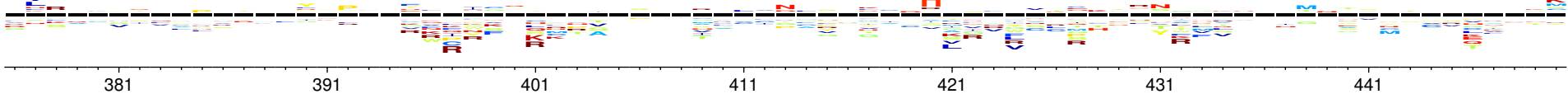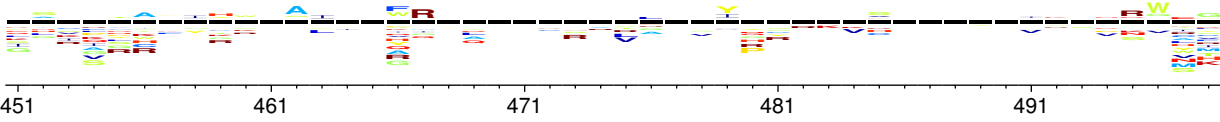

Supplement: S1 File — This zip file contains the computer code and required input files for the deep mutational scanning data analysis reported in this manuscript. (ZIP) [file pbio.3000008.s023.zip › 2018_NP_DMS-master/preferences/HSF1i39vsDMSO37-rep3_diffsel_logoplot.pdf]

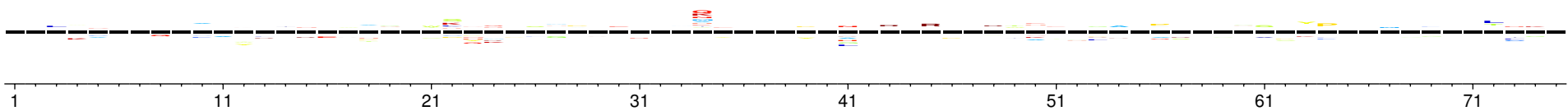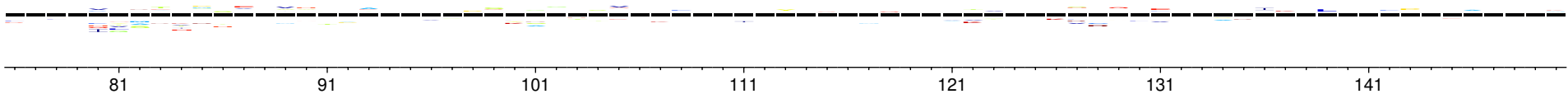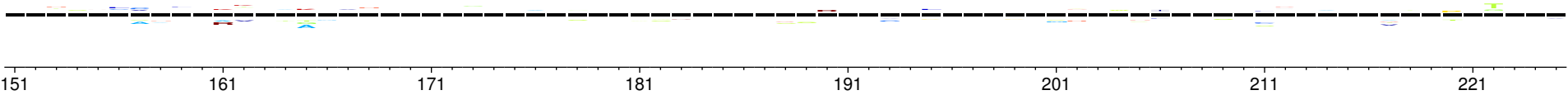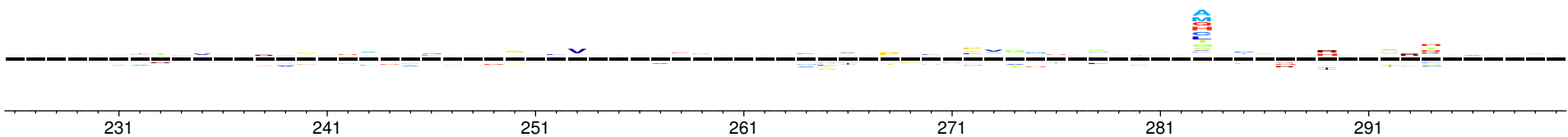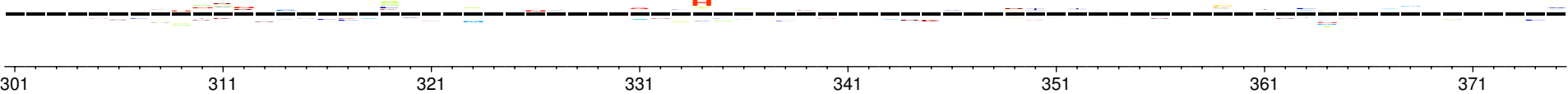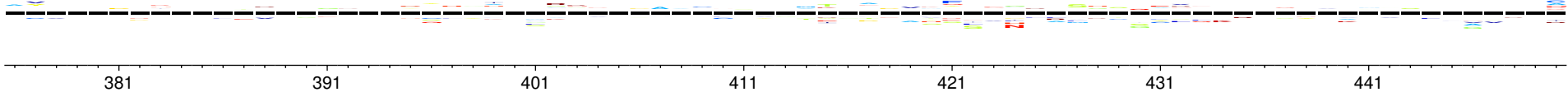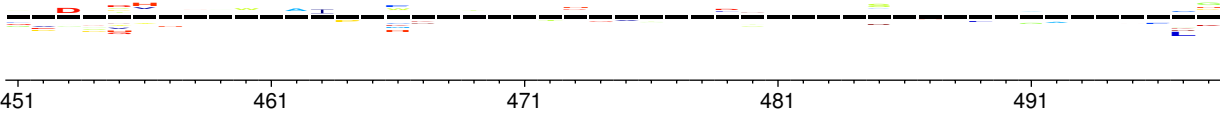

Supplement: S1 File — This zip file contains the computer code and required input files for the deep mutational scanning data analysis reported in this manuscript. (ZIP) [file pbio.3000008.s023.zip › 2018_NP_DMS-master/preferences/HSF1i39vsDMSO39-rep1_diffsel_logoplot.pdf]

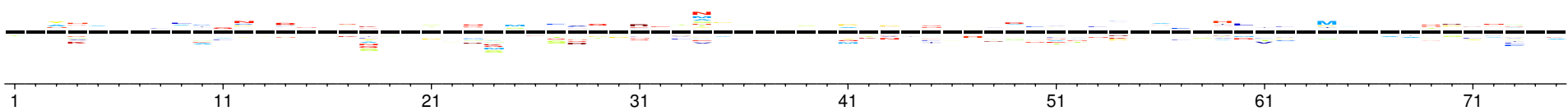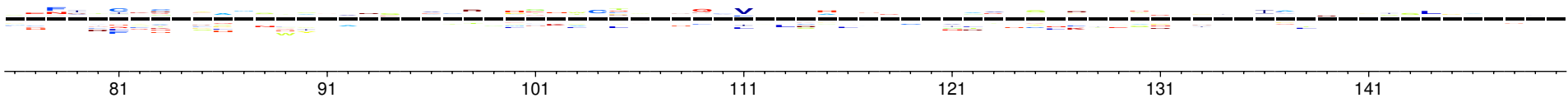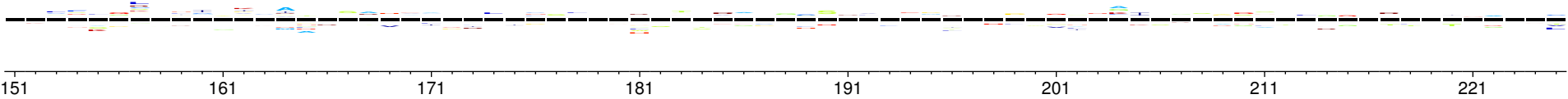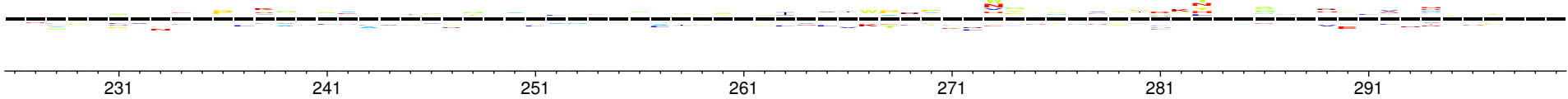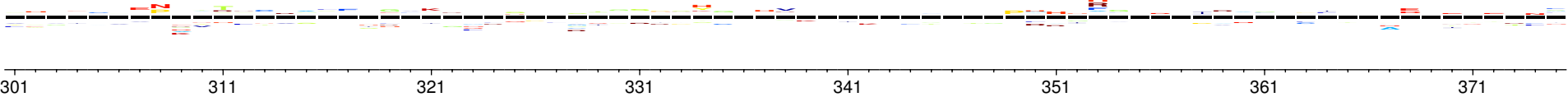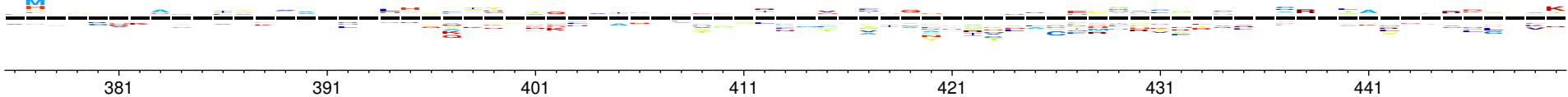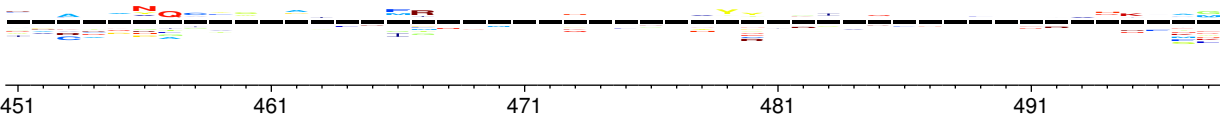

Supplement: S1 File — This zip file contains the computer code and required input files for the deep mutational scanning data analysis reported in this manuscript. (ZIP) [file pbio.3000008.s023.zip › 2018_NP_DMS-master/preferences/HSF1i39vsDMSO39-rep2_diffsel_logoplot.pdf]

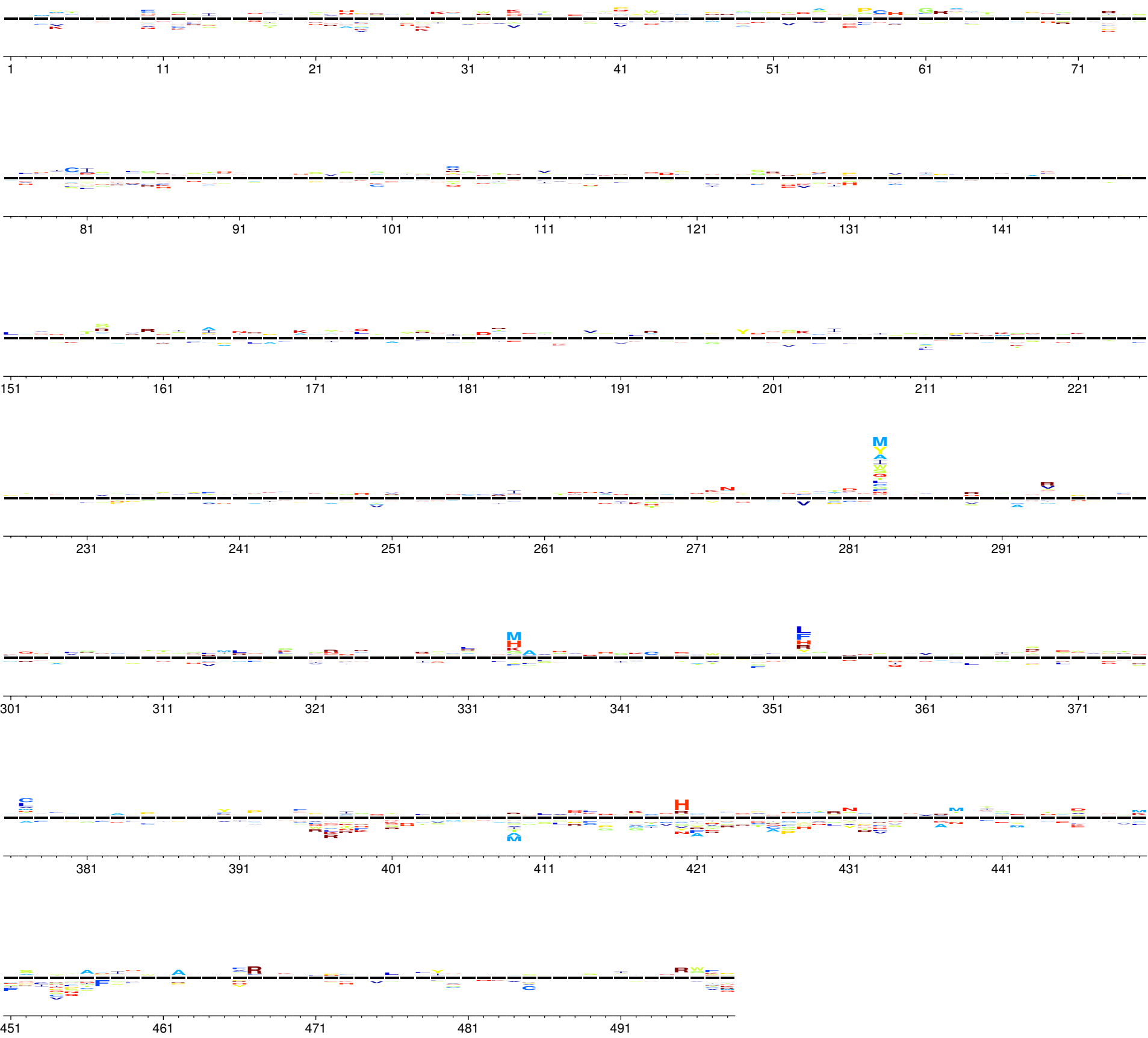

Supplement: S1 File — This zip file contains the computer code and required input files for the deep mutational scanning data analysis reported in this manuscript. (ZIP) [file pbio.3000008.s023.zip › 2018_NP_DMS-master/preferences/HSF1i39vsDMSO39-rep3_diffsel_logoplot.pdf]

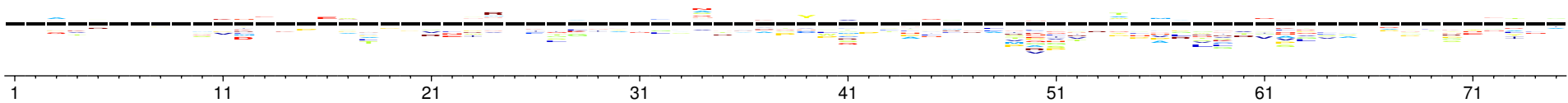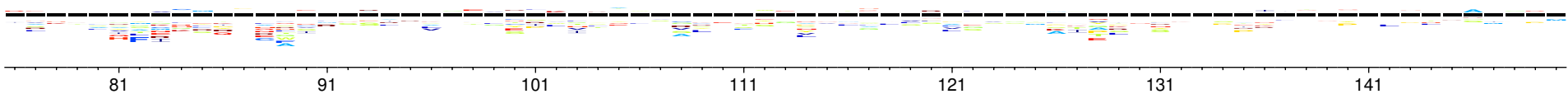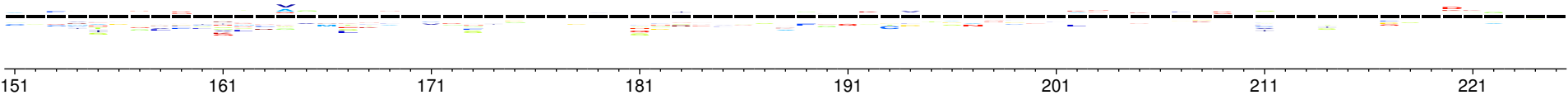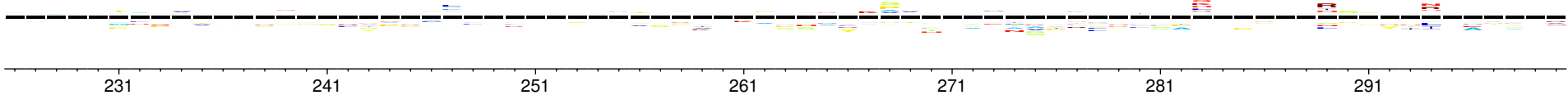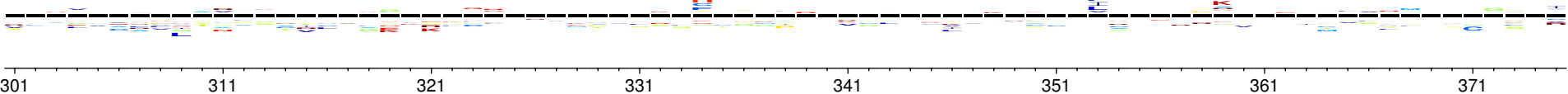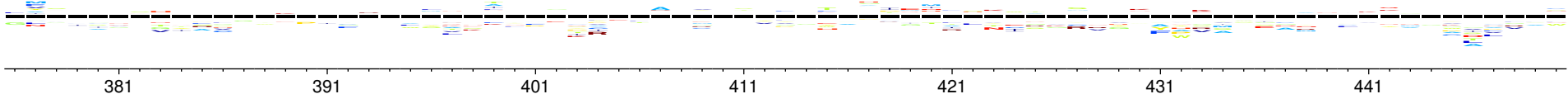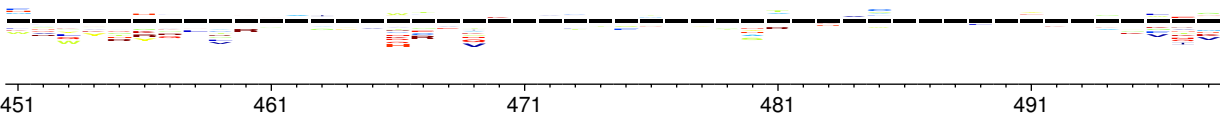

Supplement: S1 File — This zip file contains the computer code and required input files for the deep mutational scanning data analysis reported in this manuscript. (ZIP) [file pbio.3000008.s023.zip › 2018_NP_DMS-master/preferences/Hsp90i39vsDMSO37-rep1_diffsel_logoplot.pdf]

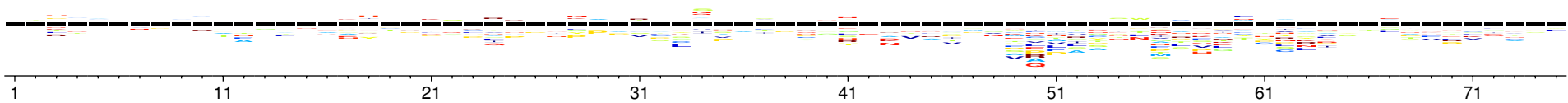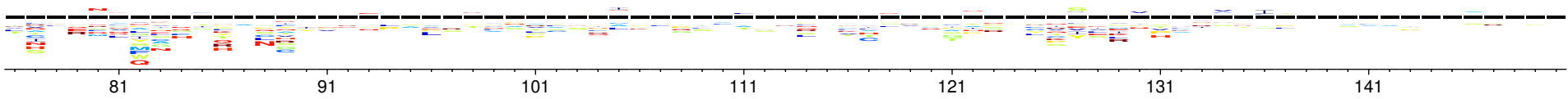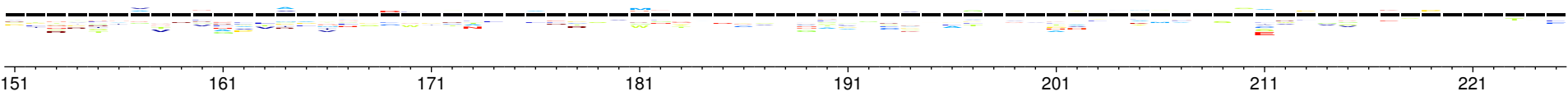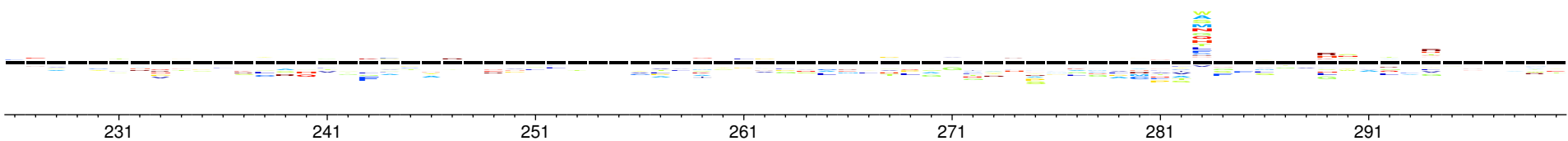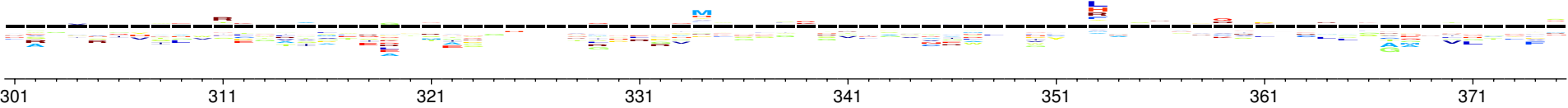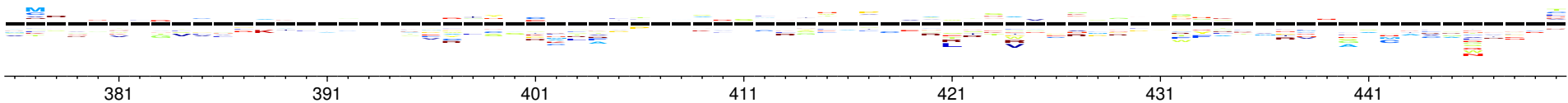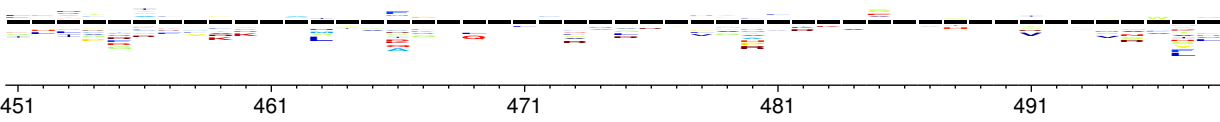

Supplement: S1 File — This zip file contains the computer code and required input files for the deep mutational scanning data analysis reported in this manuscript. (ZIP) [file pbio.3000008.s023.zip › 2018_NP_DMS-master/preferences/Hsp90i39vsDMSO37-rep2_diffsel_logoplot.pdf]

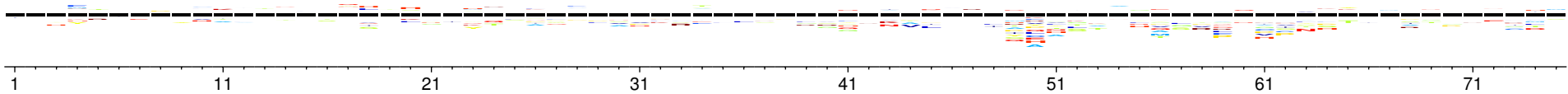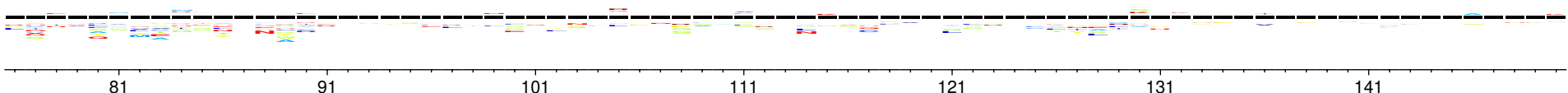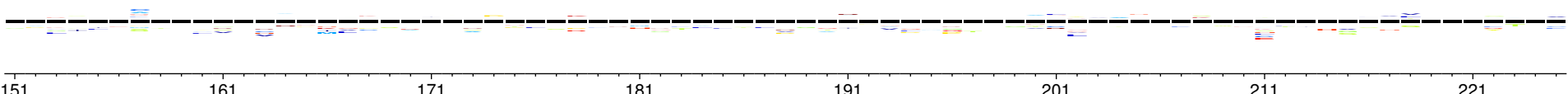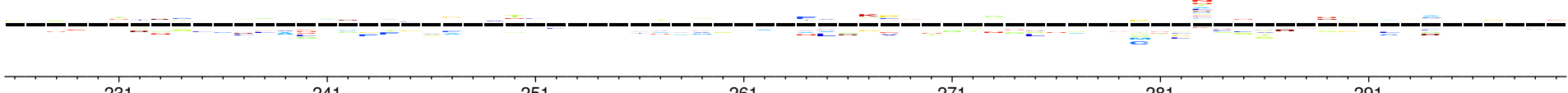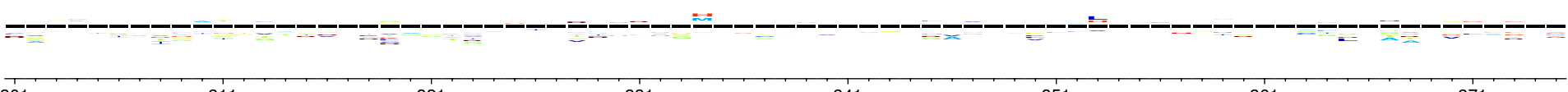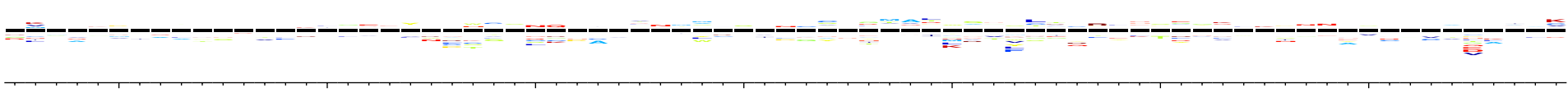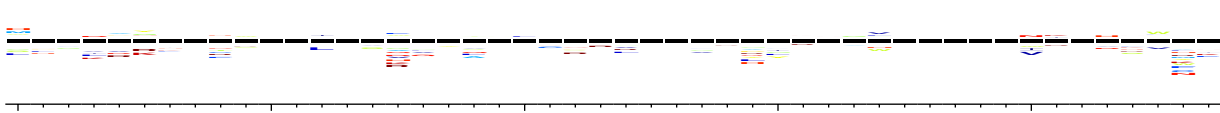

Supplement: S1 File — This zip file contains the computer code and required input files for the deep mutational scanning data analysis reported in this manuscript. (ZIP) [file pbio.3000008.s023.zip › 2018_NP_DMS-master/preferences/Hsp90i39vsDMSO37-rep3_diffsel_logoplot.pdf]

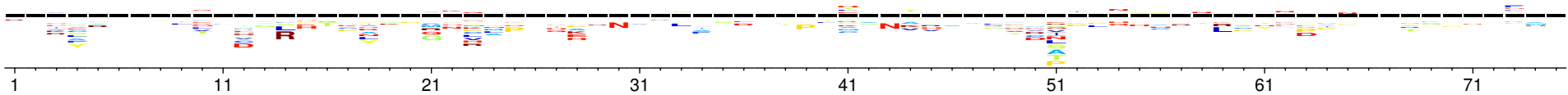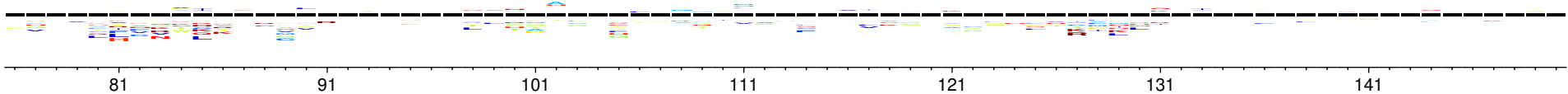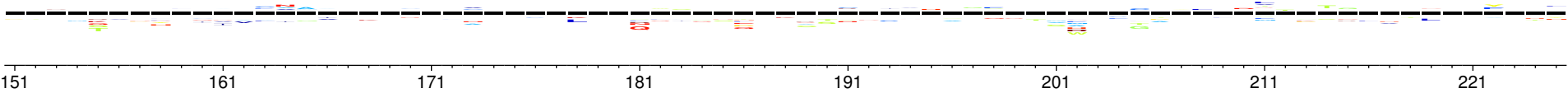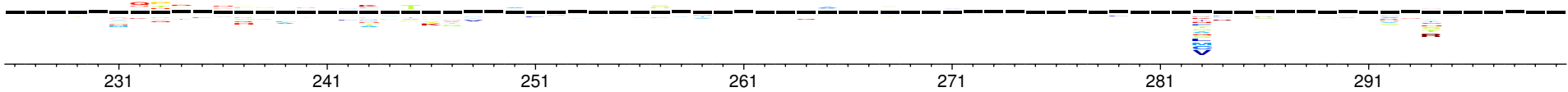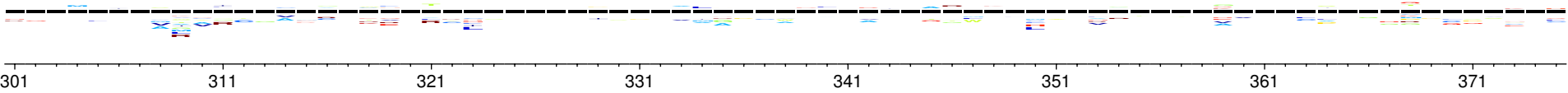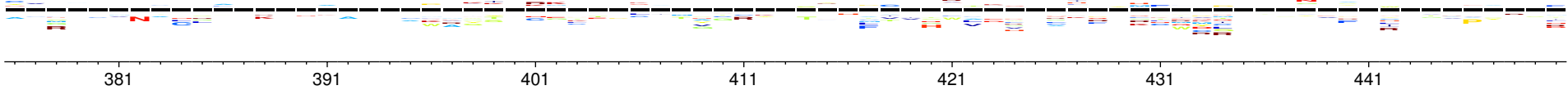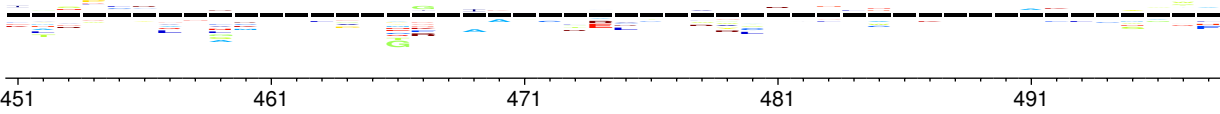

Supplement: S1 File — This zip file contains the computer code and required input files for the deep mutational scanning data analysis reported in this manuscript. (ZIP) [file pbio.3000008.s023.zip › 2018_NP_DMS-master/preferences/MxA_logoplot.pdf]

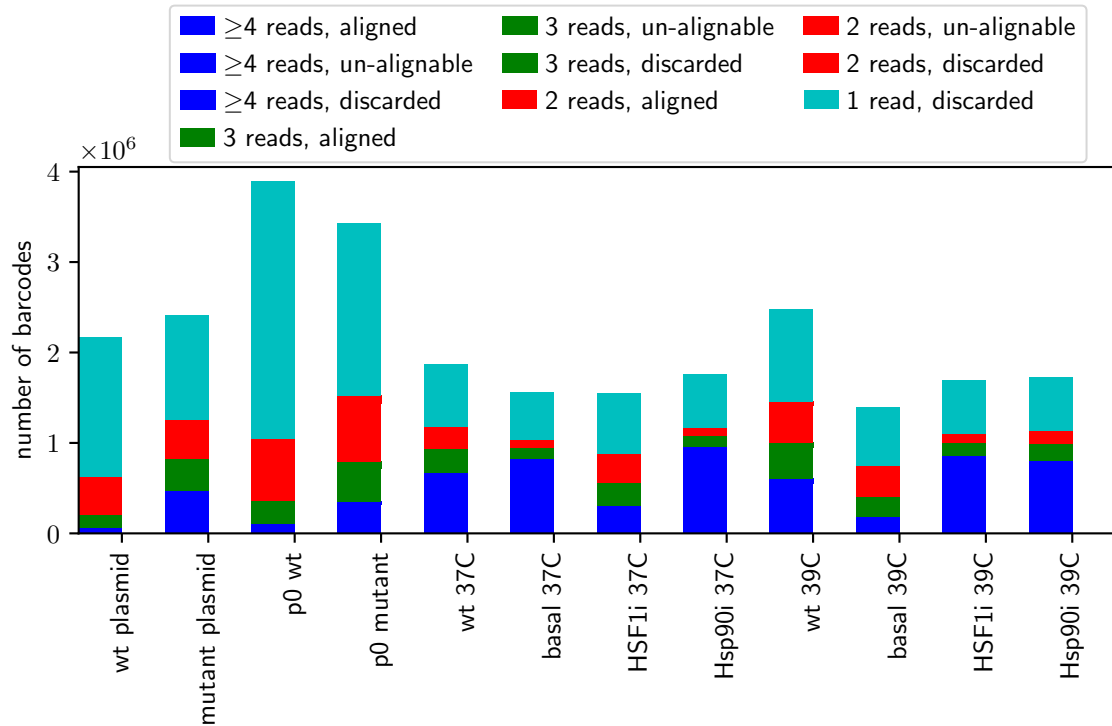

Supplement: S1 File — This zip file contains the computer code and required input files for the deep mutational scanning data analysis reported in this manuscript. (ZIP) [file pbio.3000008.s023.zip › 2018_NP_DMS-master/replicate-1/alignmentsummary_barcodes.pdf]

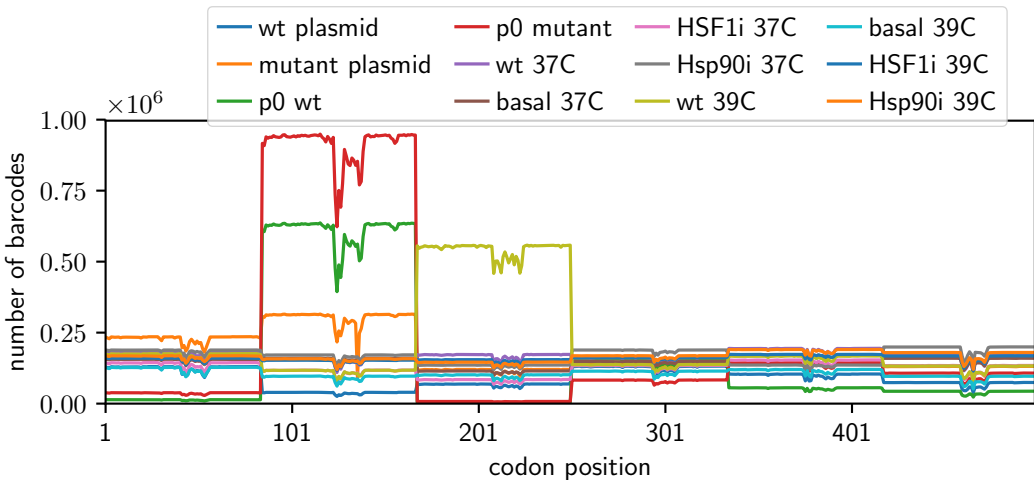

Supplement: S1 File — This zip file contains the computer code and required input files for the deep mutational scanning data analysis reported in this manuscript. (ZIP) [file pbio.3000008.s023.zip › 2018_NP_DMS-master/replicate-1/alignmentsummary_depth.pdf]

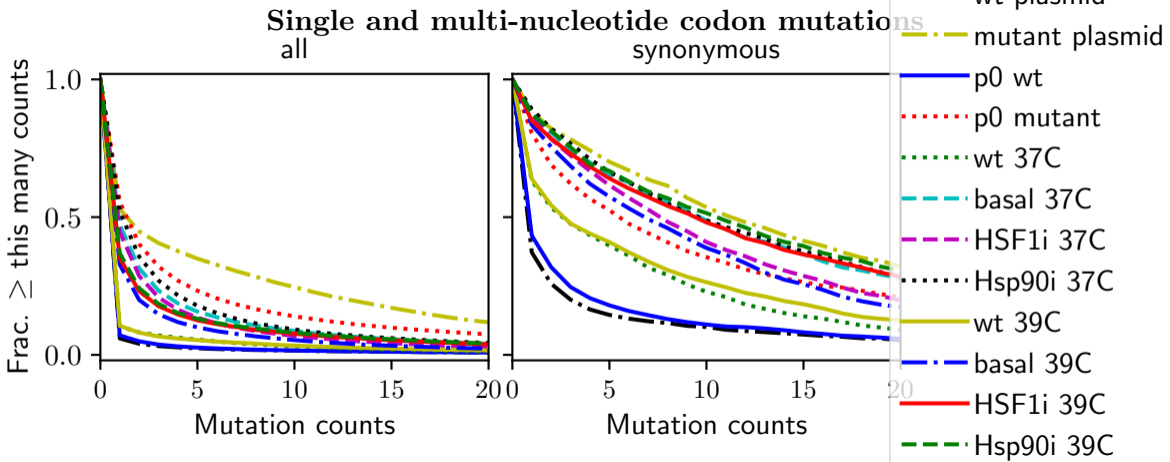

Supplement: S1 File — This zip file contains the computer code and required input files for the deep mutational scanning data analysis reported in this manuscript. (ZIP) [file pbio.3000008.s023.zip › 2018_NP_DMS-master/replicate-1/alignmentsummary_mutcounts_all.pdf]

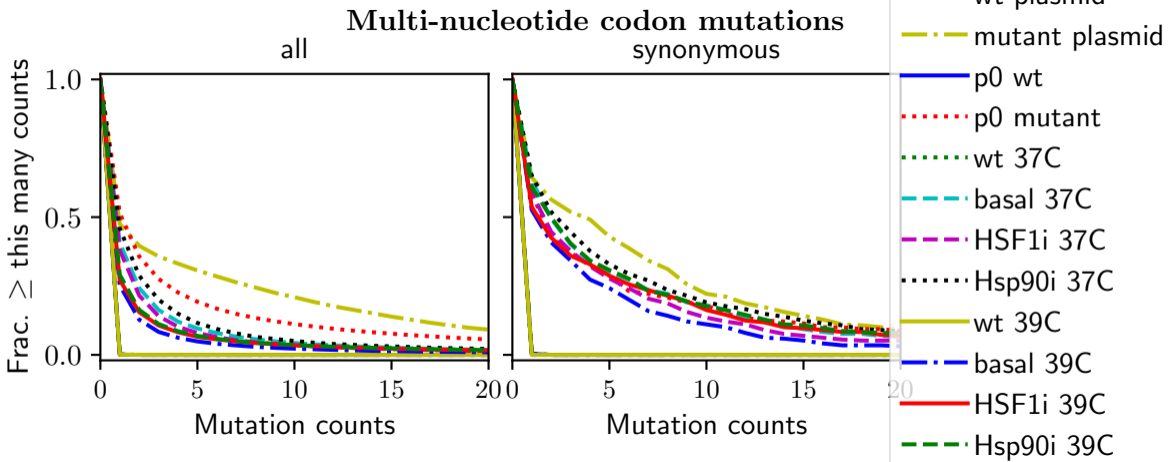

Supplement: S1 File — This zip file contains the computer code and required input files for the deep mutational scanning data analysis reported in this manuscript. (ZIP) [file pbio.3000008.s023.zip › 2018_NP_DMS-master/replicate-1/alignmentsummary_mutcounts_multi_nt.pdf]

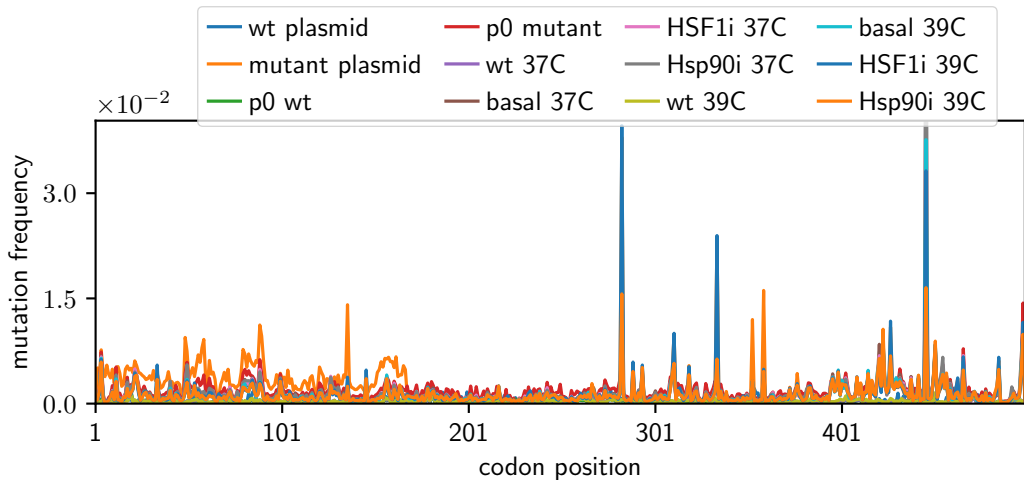

Supplement: S1 File — This zip file contains the computer code and required input files for the deep mutational scanning data analysis reported in this manuscript. (ZIP) [file pbio.3000008.s023.zip › 2018_NP_DMS-master/replicate-1/alignmentsummary_mutdepth.pdf]

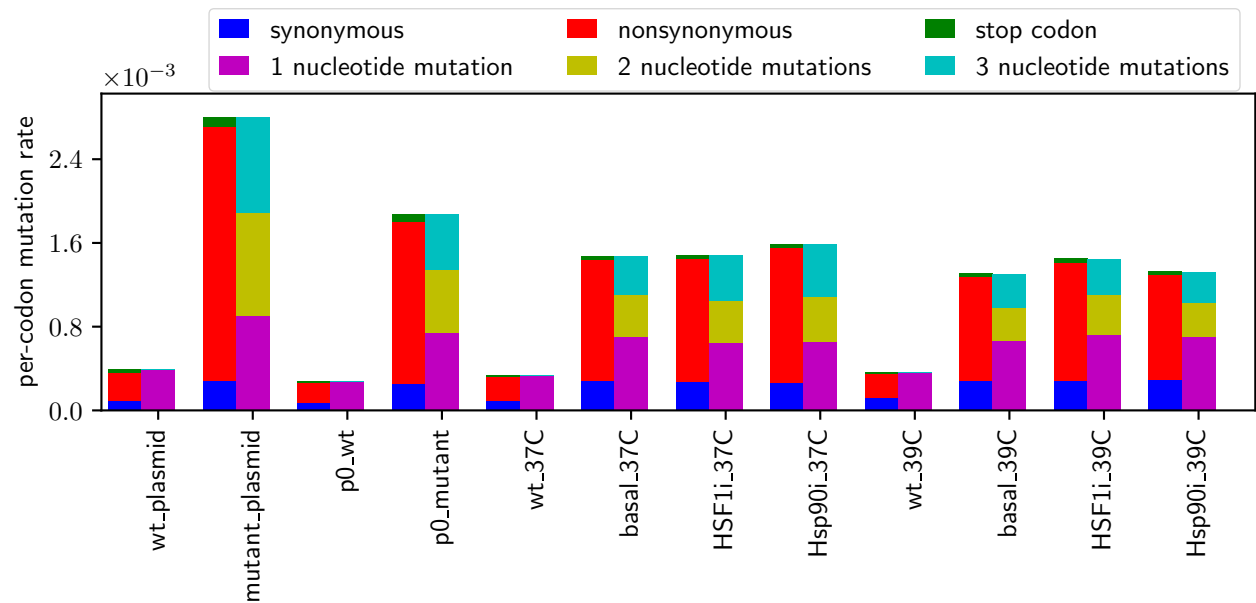

Supplement: S1 File — This zip file contains the computer code and required input files for the deep mutational scanning data analysis reported in this manuscript. (ZIP) [file pbio.3000008.s023.zip › 2018_NP_DMS-master/replicate-1/alignmentsummary_mutfreqs.pdf]

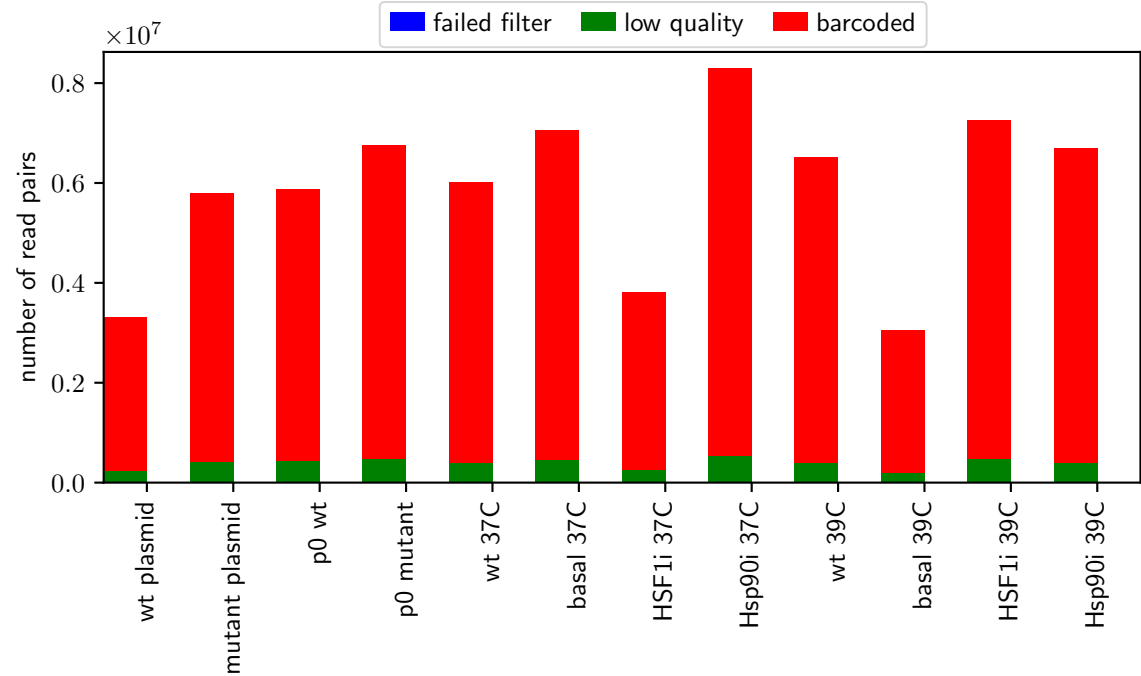

Supplement: S1 File — This zip file contains the computer code and required input files for the deep mutational scanning data analysis reported in this manuscript. (ZIP) [file pbio.3000008.s023.zip › 2018_NP_DMS-master/replicate-1/alignmentsummary_reads.pdf]

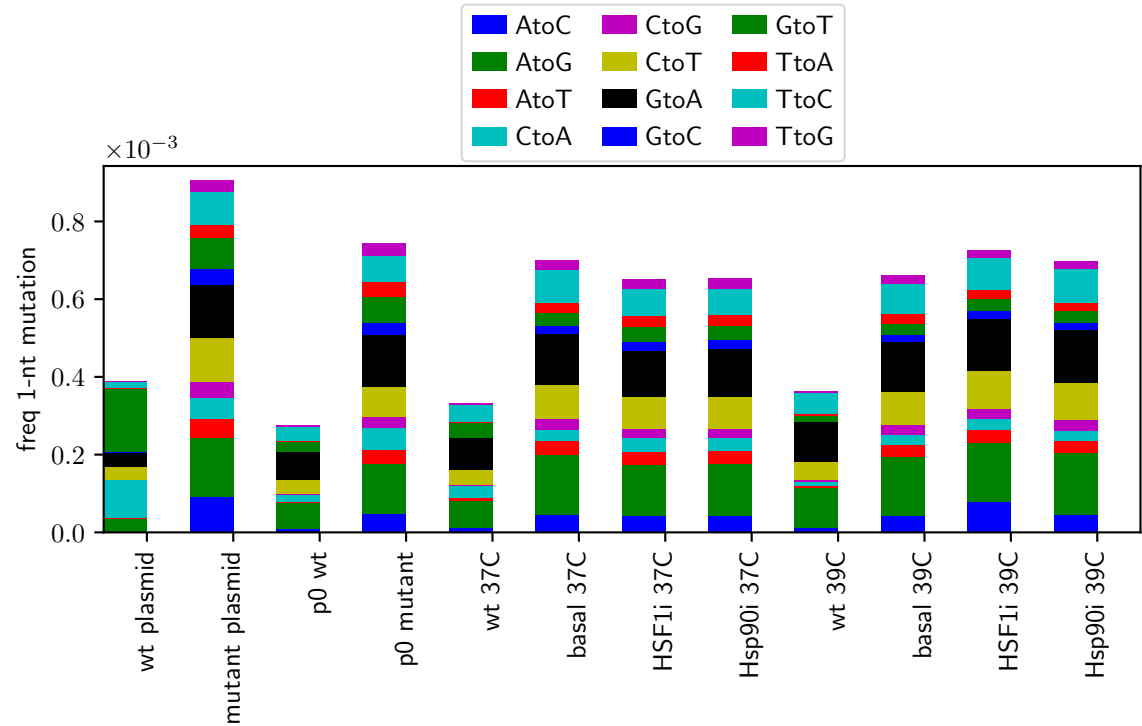

Supplement: S1 File — This zip file contains the computer code and required input files for the deep mutational scanning data analysis reported in this manuscript. (ZIP) [file pbio.3000008.s023.zip › 2018_NP_DMS-master/replicate-1/alignmentsummary_singlemuttypes.pdf]

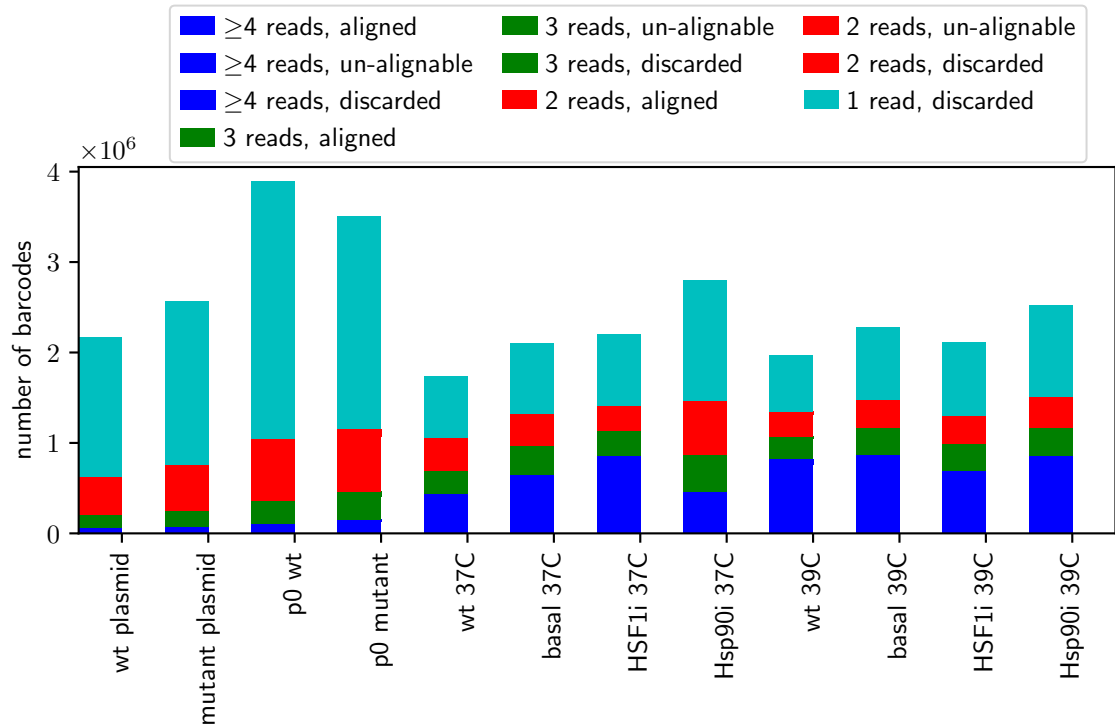

Supplement: S1 File — This zip file contains the computer code and required input files for the deep mutational scanning data analysis reported in this manuscript. (ZIP) [file pbio.3000008.s023.zip › 2018_NP_DMS-master/replicate-2/alignmentsummary_barcodes.pdf]

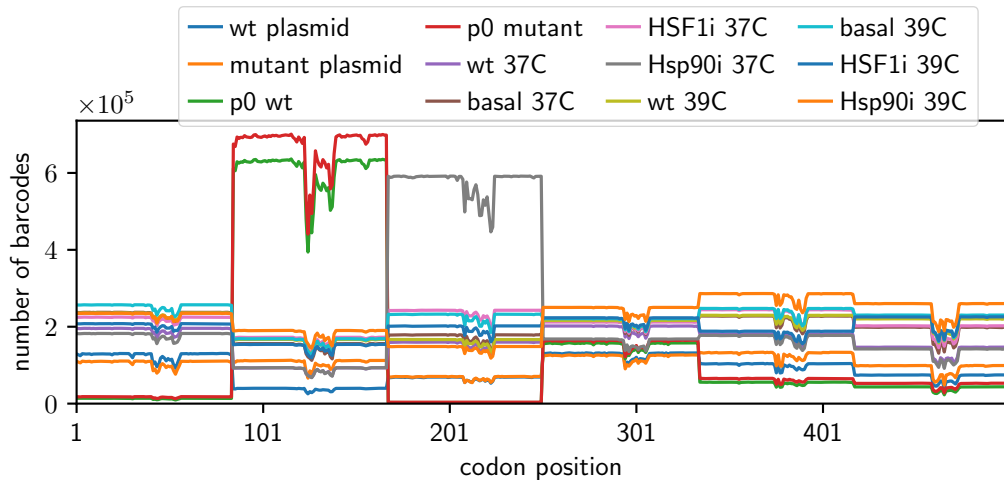

Supplement: S1 File — This zip file contains the computer code and required input files for the deep mutational scanning data analysis reported in this manuscript. (ZIP) [file pbio.3000008.s023.zip › 2018_NP_DMS-master/replicate-2/alignmentsummary_depth.pdf]

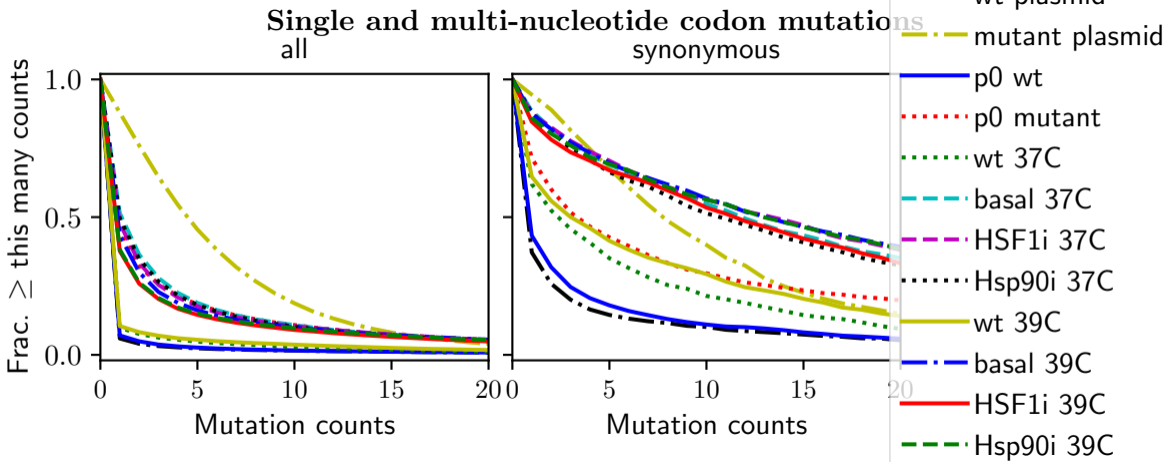

Supplement: S1 File — This zip file contains the computer code and required input files for the deep mutational scanning data analysis reported in this manuscript. (ZIP) [file pbio.3000008.s023.zip › 2018_NP_DMS-master/replicate-2/alignmentsummary_mutcounts_all.pdf]

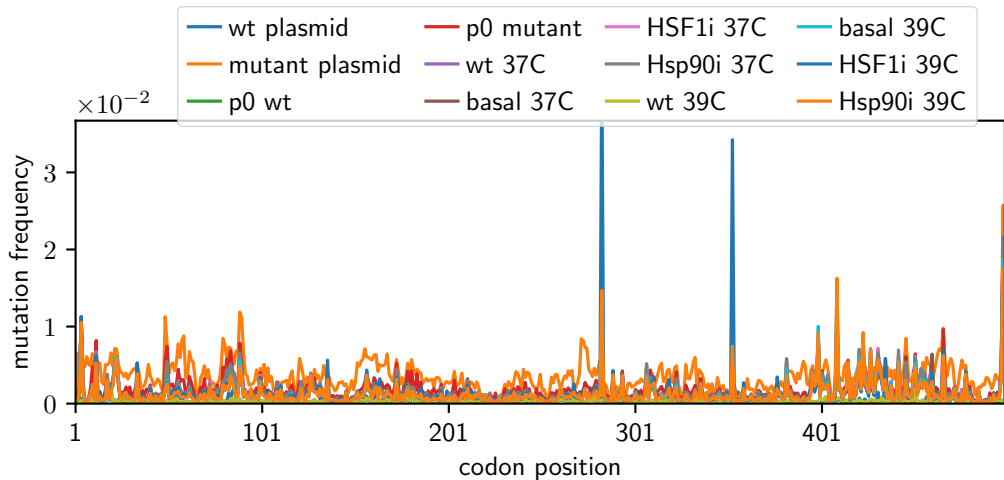

Supplement: S1 File — This zip file contains the computer code and required input files for the deep mutational scanning data analysis reported in this manuscript. (ZIP) [file pbio.3000008.s023.zip › 2018_NP_DMS-master/replicate-2/alignmentsummary_mutdepth.pdf]

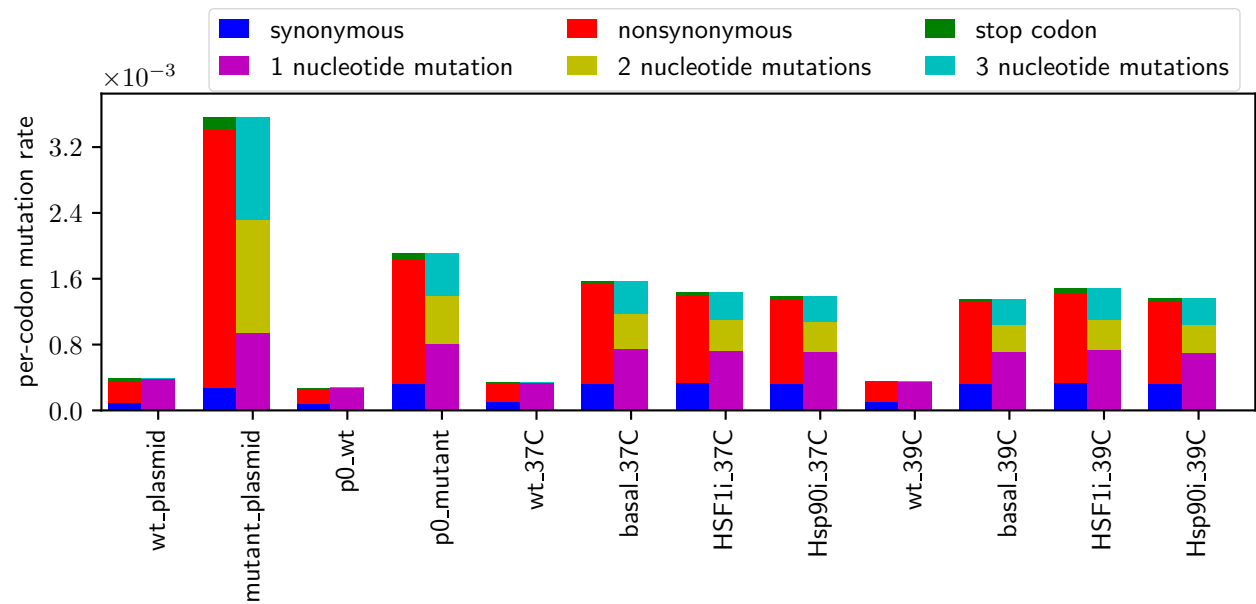

Supplement: S1 File — This zip file contains the computer code and required input files for the deep mutational scanning data analysis reported in this manuscript. (ZIP) [file pbio.3000008.s023.zip › 2018_NP_DMS-master/replicate-2/alignmentsummary_mutfreqs.pdf]

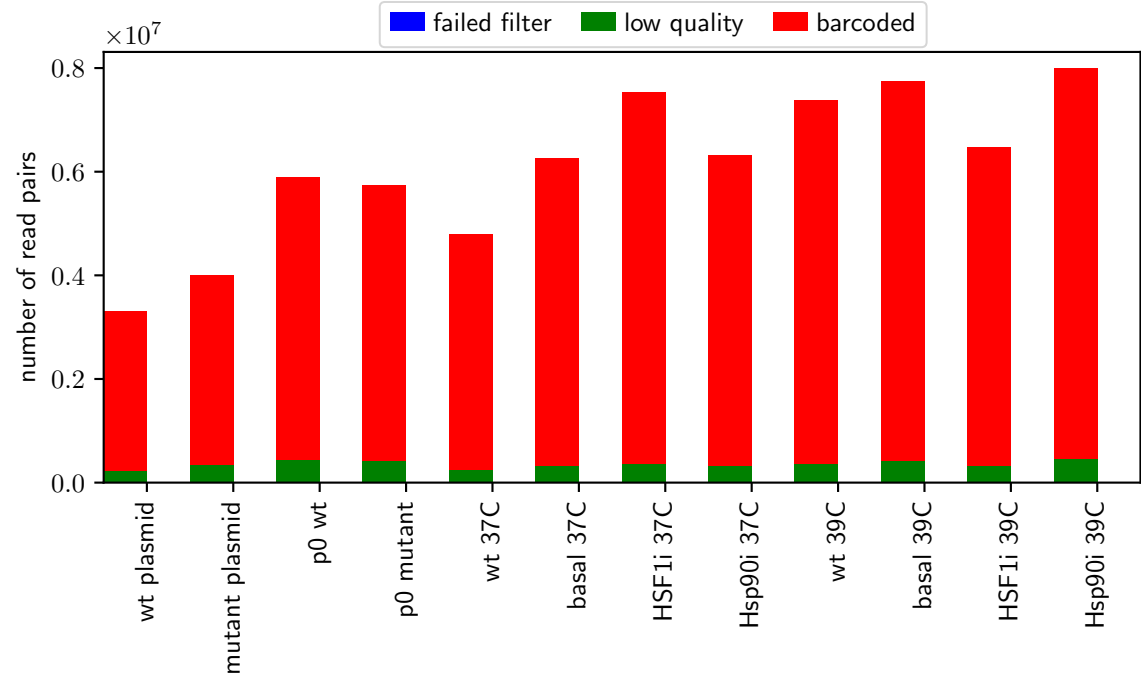

Supplement: S1 File — This zip file contains the computer code and required input files for the deep mutational scanning data analysis reported in this manuscript. (ZIP) [file pbio.3000008.s023.zip › 2018_NP_DMS-master/replicate-2/alignmentsummary_reads.pdf]

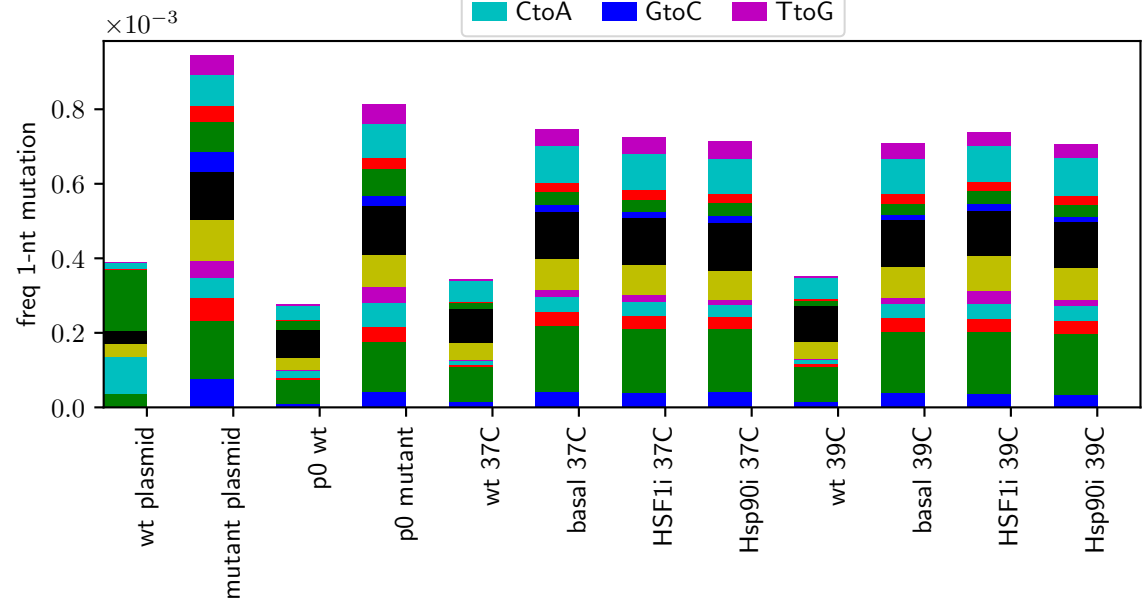

Supplement: S1 File — This zip file contains the computer code and required input files for the deep mutational scanning data analysis reported in this manuscript. (ZIP) [file pbio.3000008.s023.zip › 2018_NP_DMS-master/replicate-2/alignmentsummary_singlemuttypes.pdf]

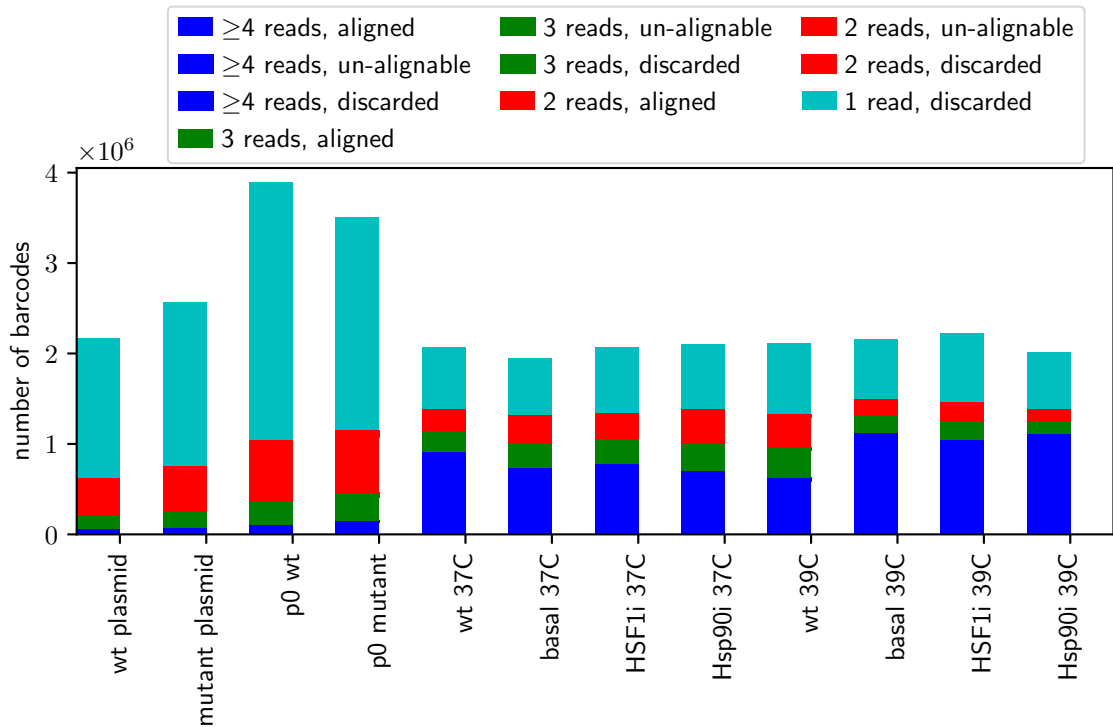

Supplement: S1 File — This zip file contains the computer code and required input files for the deep mutational scanning data analysis reported in this manuscript. (ZIP) [file pbio.3000008.s023.zip › 2018_NP_DMS-master/replicate-3/alignmentsummary_barcodes.pdf]

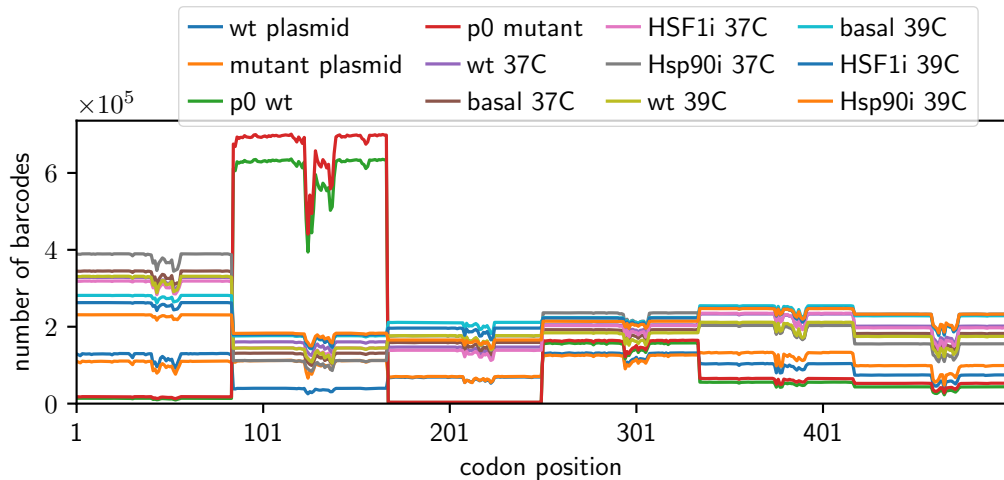

Supplement: S1 File — This zip file contains the computer code and required input files for the deep mutational scanning data analysis reported in this manuscript. (ZIP) [file pbio.3000008.s023.zip › 2018_NP_DMS-master/replicate-3/alignmentsummary_depth.pdf]

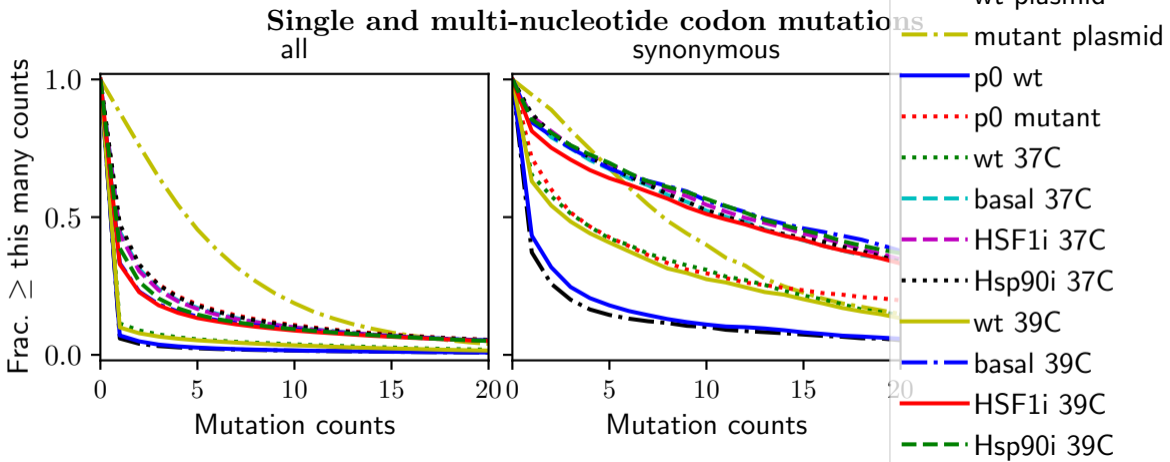

Supplement: S1 File — This zip file contains the computer code and required input files for the deep mutational scanning data analysis reported in this manuscript. (ZIP) [file pbio.3000008.s023.zip › 2018_NP_DMS-master/replicate-3/alignmentsummary_mutcounts_all.pdf]

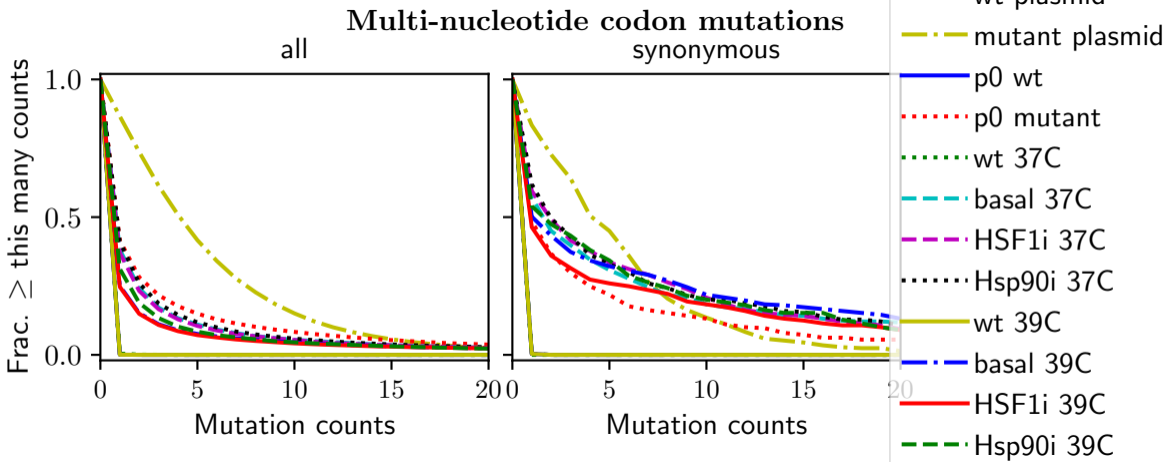

Supplement: S1 File — This zip file contains the computer code and required input files for the deep mutational scanning data analysis reported in this manuscript. (ZIP) [file pbio.3000008.s023.zip › 2018_NP_DMS-master/replicate-3/alignmentsummary_mutcounts_multi_nt.pdf]

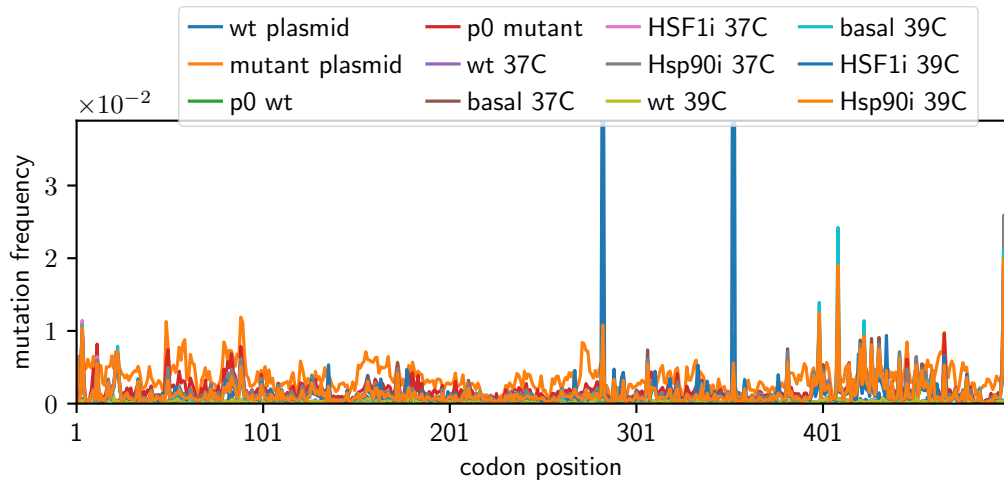

Supplement: S1 File — This zip file contains the computer code and required input files for the deep mutational scanning data analysis reported in this manuscript. (ZIP) [file pbio.3000008.s023.zip › 2018_NP_DMS-master/replicate-3/alignmentsummary_mutdepth.pdf]

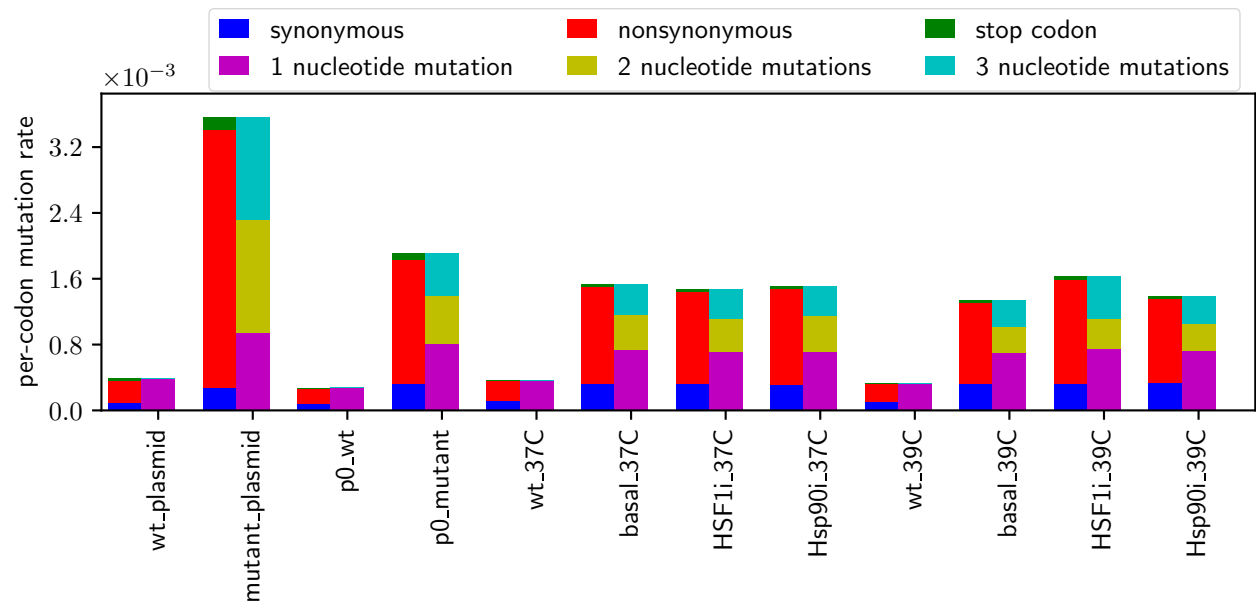

Supplement: S1 File — This zip file contains the computer code and required input files for the deep mutational scanning data analysis reported in this manuscript. (ZIP) [file pbio.3000008.s023.zip › 2018_NP_DMS-master/replicate-3/alignmentsummary_mutfreqs.pdf]

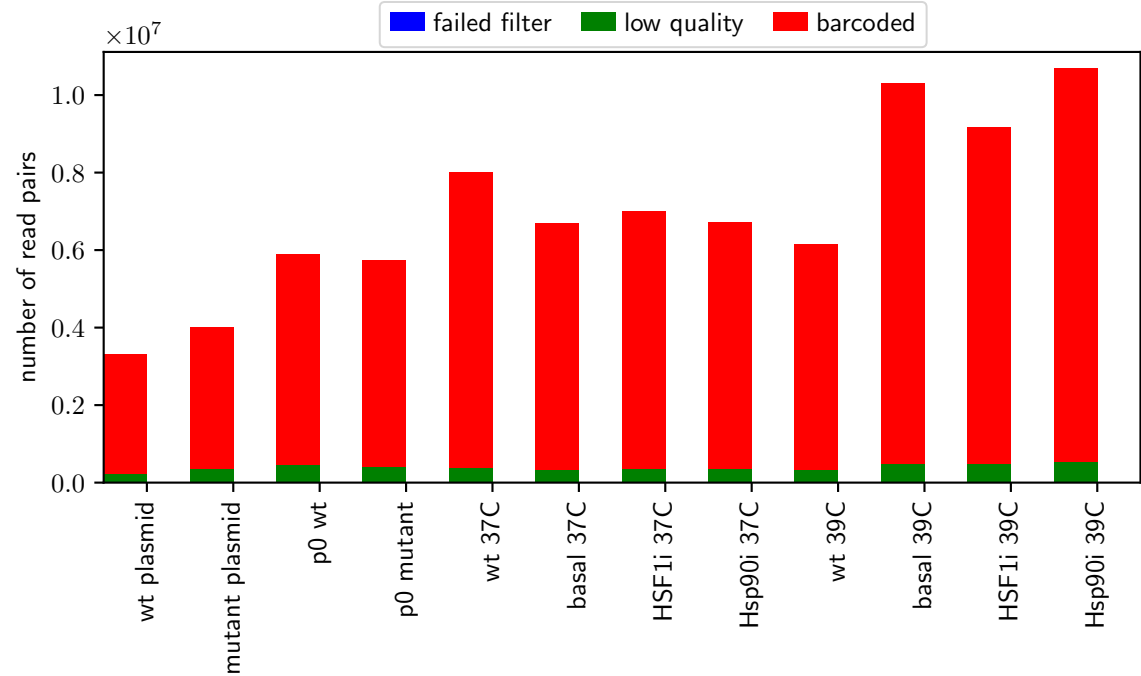

Supplement: S1 File — This zip file contains the computer code and required input files for the deep mutational scanning data analysis reported in this manuscript. (ZIP) [file pbio.3000008.s023.zip › 2018_NP_DMS-master/replicate-3/alignmentsummary_reads.pdf]

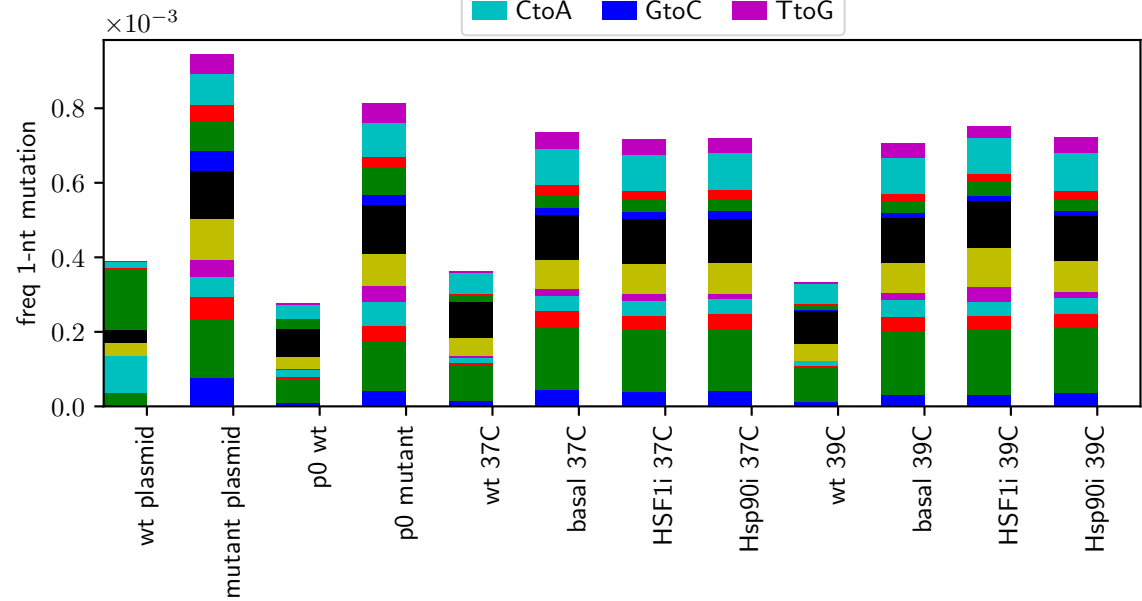

Supplement: S1 File — This zip file contains the computer code and required input files for the deep mutational scanning data analysis reported in this manuscript. (ZIP) [file pbio.3000008.s023.zip › 2018_NP_DMS-master/replicate-3/alignmentsummary_singlemuttypes.pdf]
