# Supplementary material for: Destabilized adaptive influenza variants critical for innate immune system escape are potentiated by host chaperones
Source: PLoS Biol. 2018 Sep 17;16(9):e3000008. doi: 10.1371/journal.pbio.3000008 (PMC6160216; doi:10.1371/journal.pbio.3000008)
Supplement: S1 File — This zip file contains the computer code and required input files for the deep mutational scanning data analysis reported in this manuscript. (ZIP) [file alignmentsummary_mutcounts_multi_nt.pdf › 2018_NP_DMS-master/replicate-2/alignmentsummary_mutcounts_multi_nt.pdf]

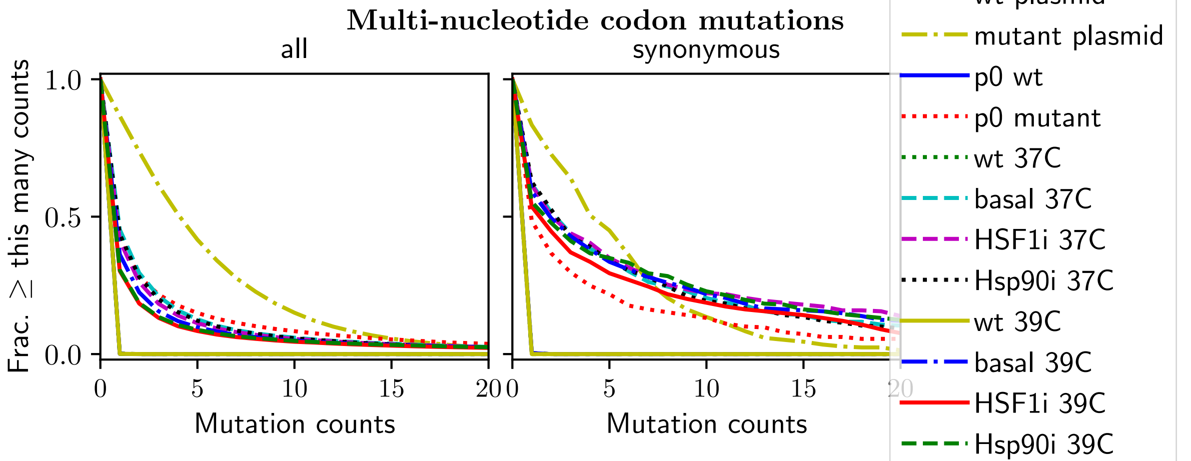
Multi-nucleotide codon mutations
all
synonymous
Frac. ≥ this many counts
1.0
0.5
0.0
0
5
10
15
20
Mutation counts
Mutation counts
wt plasmid
mutant plasmid
p0 wt
p0 mutant
wt 37C
basal 37C
HSF1i 37C
Hsp90i 37C
wt 39C
basal 39C
HSF1i 39C
Hsp90i 39C
